# Revisiting the evidence on caffeine mouth rinse: effects on exercise and cognitive performance: a meta-analytic review

**DOI:** 10.1080/15502783.2026.2638903

**Published:** 2026-03-03

**Authors:** Hengzhi Deng, Xiaohan Fan, Tianyu Song, Nasnoor Juzaily bin Mohd Nasiruddin, Abdullah Al-Hadi Ahmad Fuaad, Mohamed Nashrudin bin Naharudin

**Affiliations:** a Faculty of Sports and Exercise Science, University of Malaya, Kuala Lumpur, Malaysia; b Faculty of Science, University of Malaya, Kuala Lumpur, Malaysia

**Keywords:** Caffeine mouth rinsing, exercise performance, cognitive performance, meta-analysis, dosing strategy

## Abstract

**Background:**

Caffeine mouth rinsing (Caff-MR) may activate oropharyngeal receptors and rapidly engage central networks for motivation, attention, and pacing without systemic absorption. The only prior meta-analysis found no stable ergogenic effect, yet the evidence base has continued to expand and remains heterogeneous.

**Methods:**

Six electronic databases were searched up to 2 October 2025 for Caff-MR studies on exercise and cognitive outcomes. Study quality was assessed using modified PEDro and RoB-2. Three-level meta-analyses synthesized both outcomes. Prespecified moderators were sex, training status, habitual caffeine use, feeding state, exercise or cognitive type, rinse duration, and total oral exposure. Sensitivity analyses addressed assumed within-subject correlations, outliers, and influential cases.

**Results:**

Thirty-one studies (k = 167 effects) met inclusion. Caff-MR was associated with trivial-to-small improvements in exercise performance (k = 114; g = 0.12, *p* = 0.01). Benefits were most consistent for aerobic endurance and in the fed state; ~5-s rinses outperformed longer durations. Primary dose–response suggested a U-shape (32–133 mg window), but this pattern was not robust to outlier removal; under 5-s conditions, higher total exposure related negatively to performance. Cognitive effects were inconsistent overall (k = 53; g = 0.23, *p* = 0.07), yet after outlier removal the overall and speed-based effects reached significance, whereas accuracy remained variable. Risk of bias was predominantly “some concerns”; GRADE certainty was moderate (exercise) and very low/low (cognition).

**Conclusions:**

Caff-MR is a practical, ingestion-free strategy yielding small, context-dependent benefits, optimized by brief (~5 s) rinses and moderate exposure, particularly for aerobic endurance. Standardized, well-powered trials are needed to refine dosing, timing, and cognitive applications.

## Key points

- Caff-MR yields **trivial-to-small** exercise benefits, most reliably in **aerobic endurance** and the **fed state**.
- **Brief (~5 s) rinses** perform better than longer durations; escalating exposure does not improve outcomes.
- Primary **U-shaped** dose–response was not robust; **within 5-s rinses, higher exposure may negatively relate to exercise performance.**
- Cognitive effects are **task-dependent**; processing speed shows greater sensitivity than accuracy after outlier control.

## Graphical abstract

**Figure:**
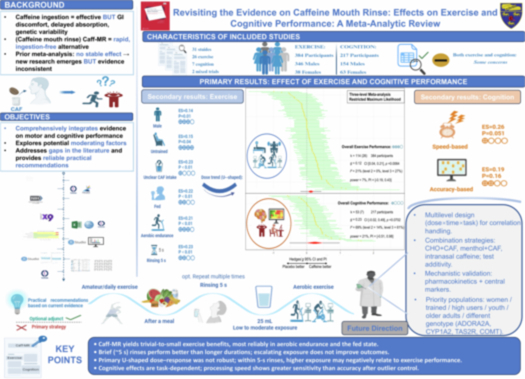

## 1. Introduction

Caffeine is among the most widely used psychoactive substances worldwide and has been extensively studied as an ergogenic aid [1,2]. Traditional oral ingestion of caffeine has been shown to improve endurance, strength, and power performance, largely through its central nervous system effects mediated by adenosine receptor antagonism [3]. However, ingestion can be accompanied by gastrointestinal discomfort, delayed absorption, and variable individual responses due to genetic and metabolic differences, such as CYP1A2 polymorphisms [4]. These limitations have prompted interest in caffeine mouth rinsing (Caff-MR), in which a solution is swilled briefly and expectorated to elicit rapid central effects without systemic uptake [5].

Existing neuroimaging studies have found that simply rinsing with caffeine without ingesting it may activate the insula, orbitofrontal cortex, and striatum, thereby enhancing central drive and cognitive control [6]. Consistent with these findings, several trials have reported improvements in repeated-sprint performance, self-paced cycling, or muscular endurance following Caff-MR [7–9]. However, other studies have failed to demonstrate benefits for time-trials, maximal strength, or running performance [5,10,11], leaving the contexts in which this strategy is effective uncertain.

Previous systematic reviews and meta-analyses have attempted to synthesise these findings, yet their conclusions remain unclear. A recent meta-analysis that included 16 studies reported a very small and nonsignificant effect of Caff-MR on exercise outcomes, emphasising the large variability in supplementation protocols and participant characteristics [12]. Another review that examined 18 studies (15 physical and 3 cognitive) observed consistent improvements in cognitive performance but found the evidence for physical outcomes to be inconsistent, with benefits more likely when rinsing was repeated during exercise or performed in a fasted state [13]. Similarly, a separate review of 11 randomised crossover trials identified only three studies that demonstrated clear ergogenic effects, whereas the majority showed no meaningful improvements [14]. Collectively, these earlier works highlighted substantial heterogeneity across trials in terms of caffeine concentration, rinse frequency, training status, and habitual caffeine intake. Importantly, although cognitive outcomes have been reported in individual studies, no meta-analysis has yet quantitatively synthesised these effects, despite their potential relevance for sports that rely on reaction time, accuracy, and decision-making.

Despite these uncertainties, interest in Caff-MR remains high, and the evidence base has expanded considerably in recent years. Some recent investigations have reported meaningful improvements in both physical [15–17] and cognitive outcomes [18,19], while others continue to find limited or context-dependent effects [20,21]. This growing but conflicting body of evidence underscores the need for an updated and more comprehensive synthesis.

Therefore, this systematic review and three-level meta-analysis aims to provide a precise and up-to-date quantification of the effects of Caff-MR on exercise and cognitive performance compared with placebo. In addition, this work seeks to identify the conditions under which these effects are most pronounced by examining key moderators such as exercise or cognitive task type, caffeine dose, sex, training status, and habitual caffeine use. By clarifying the magnitude, consistency, and contextual relevance of this intervention, the present study addresses ongoing uncertainty in the field and offers meaningful guidance for researchers, practitioners, and athletes who are considering non-ingestive strategies to enhance performance while avoiding gastrointestinal discomfort associated with caffeine ingestion.

## 2. Methods

This systematic review and meta-analysis was pre-registered on the Open Science Framework (OSF) on September 25, 2025 (Registration: osf.io/8r24c) and conducted in accordance with the PRISMA 2020 guidelines [22]. The completed PRISMA checklist is available in Electronic Supplementary Material Appendix S1.

### 2.1. Eligibility criteria

This review follows the PICOs framework: (1) Participants: healthy adults; (2) Intervention: Caff-MR; (3) Comparison: non-caffeinated placebo mouth rinse; (4) Outcomes: exercise and/or cognitive performance. Specifically, studies were included if they met all of the following criteria: (1) randomised controlled trials (RCTs) published in peer-reviewed journals; (2) investigated the effects of Caff-MR on exercise and/or cognitive performance outcomes; (3) involved healthy human participants; (4) included a non-caffeine control condition such as water or non-caloric placebo rinse; and (5) reported original experimental data in English.

Exclusion criteria were: (1) interventions involving ingestion rather than mouth rinsing; (2) co-interventions with other active ingredients (e.g. carbohydrate, menthol); (3) studies without exercise or cognitive performance-related outcomes; (4) reviews, abstracts, or non-original reports; or (5) insufficient methodological information.

### 2.2. Data sources and search strategy

A systematic search was conducted on October 2, 2025, across PubMed, Web of Science, Cochrane Library, Embase, SciELO, and SPORTDiscus. Two separate Boolean strategies were applied:

(1) "caffeine mouth rinse" OR "caffeine oral rinse" OR "caffeine mouthwash" AND "exercise" OR "performance" OR "endurance" OR "strength" OR "resistance" OR "aerobic" OR "cycling" OR "running" OR "time trial" OR "time-to-exhaustion".
(2) "caffeine mouth rinse" OR "caffeine oral rinse" OR "caffeine mouthwash" AND "cognition" OR "cognitive performance" OR attention OR memory OR "reaction time" OR "executive function" OR "mental fatigue".

No date or filter restrictions were applied.

### 2.3. Data extraction

All records were imported into Excel and EndNote 21 for de-duplication. Two independent reviewers (D.H.Z. and S.T.Y.) screened titles, abstracts, and full texts. Discrepancies were resolved by consensus. Extracted data included sample size, sex, training status, feeding condition, rinse solution and duration, and outcome measures (e.g. time to exhaustion, power output, reaction time, accuracy).

When data were not reported numerically, authors were contacted or WebPlotDigitizer (v4.8) was used for extraction [23].

### 2.4. Quality and risk of bias assessment

Methodological quality was assessed using a modified version of the Physiotherapy Evidence Database (PEDro) scale, with an additional item evaluating whether the study assessed the effectiveness of blinding to the placebo condition [24]. The total score ranged from 0 to 11, with studies categorised as excellent (10–11), good (7–9), fair (5–6), or poor (<5). Two reviewers (D.H.Z. and S.T.Y.) independently evaluated all included studies; any discrepancies were resolved through discussion, or adjudication by a third reviewer (M.N.N.) if consensus could not be reached.

In parallel, risk of bias was evaluated using the Cochrane Risk of Bias 2 (RoB 2) tool. For crossover trials, we applied the RoB 2 version specifically adapted for crossover designs, which includes an additional domain (Domain S) assessing bias arising from period effects and carryover effects. Accordingly, the following domains were evaluated: randomisation process, deviations from intended interventions, missing outcome data, outcome measurement, selection of the reported result, and period and carryover effects [25]. Assessments were performed independently by the same two reviewers, with disagreements resolved in the same manner.

### 2.5. Statistical analysis

#### 2.5.1. Effect size calculation and data synthesis

All effect size calculations adhered to the Cochrane Handbook for Systematic Reviews of Interventions (Version 6.5, 2024) [26]. Given that the majority of included studies had relatively small sample sizes, Hedges’g was selected to correct for small-sample bias when estimating standardised mean differences (SMDs) between Caff-MR and placebo conditions [27].

Because all included studies employed within-subject crossover designs, additional consideration was required for the paired-sample structure [27]. Specifically, the correlation (r) between paired measurements must be incorporated to accurately estimate standard errors and avoid inflation or underestimation. For studies reporting both pre- and post-exercise values, SMDs were derived from the mean change scores between the two conditions, and the same assumed r value was applied to the pre–post comparisons to maintain consistency and comparability across studies. For those reporting only post-intervention values, differences between Caff-MR and placebo means were used directly while maintaining the within-subject dependency.

As most trials did not report r values, a correlation of r = 0.50 was assumed for the primary analysis [28], with sensitivity analyses conducted at r = 0.20 (lower bound) and r = 0.80 (upper bound) to test robustness [29].

Effect sizes were (g) interpreted using standard thresholds: trivial (<0.2), small (0.2–0.5), medium (0.5–0.8), and large (>0.8) [30]. Detailed computational formulas and step-by-step procedures are provided in **Appendix S2**.

#### 2.5.2. Three-level meta-analysis and heterogeneity

To account for multiple outcomes nested within studies, a three-level meta-analysis was performed using the *metafor* package in R, with restricted maximum likelihood estimation (REML) [31–33]. Variance was partitioned into sampling error (level 1), within-study variance (level 2), and between-study variance (level 3) [34]. Model estimates were cross-validated using maximum likelihood (ML).

Heterogeneity was assessed using I² statistics, categorised as low (0–25%), moderate (25–50%), substantial (50–75%), or considerable (>75%) [28,35]. Prediction intervals (PIE) were calculated to provide context on expected effect distributions in future studies [36,37]. Power analyses were conducted using the *metameta* package to assess the risk of Type II error [38].

#### 2.5.3. Moderators and subgroup analysis

To explore between-study heterogeneity and derive more detailed conclusions, meta-regressions and moderator analyses were conducted for both exercise and cognitive outcomes.

For exercise performance, the following moderators were examined:

**1) Participant sex.**

**2) Training status:** Participants were classified as untrained or trained according to established participant categorisation frameworks [39]. The trained group included recreationally active, trained/developmental, well-trained/national-level, and elite/international-level athletes, while all others were categorised as untrained.

**3) Habitual caffeine intake:** Quantitative estimates of daily caffeine intake were extracted using the U.S. Department of Agriculture Food Data Central database reference values. Based on previous standards [40], participants were grouped as low (0–150 mg/day), moderate (150–300 mg/day), high (>300 mg/day) or unclear (habitual intake not reported or insufficient information to classify).

**4) Pre-exercise nutritional status:** Trials were coded into three categories according to the pre-test interval from the last meal: fed (≤4 h), fasted (>4 h), and unspecified (studies stating “maintain their regular/habitual diet” or similar without specifying an interval). When the reported range straddled the 4-h boundary (e.g. 3–5 h), studies were coded as unspecified to minimise misclassification.

The four-hour threshold for defining the postprandial (fed) state was selected based on previous experimental protocols [41,42] and is commonly used in exercise nutrition research. While the composition of the prior meal can influence gastric emptying and subsequent metabolic responses, a four-hour interval is widely considered sufficiently long for most nutrients to clear the primary absorptive phase [43]. Therefore, using this cutoff provides a practical and physiologically meaningful distinction between fed and fasted states, thereby improving comparability across studies.

**5) Exercise task type:** To better characterise the ergogenic effects of Caff-MRand improve model stability, outcomes were grouped into four physiological domains according to the predominant energy system or neuromuscular mechanism [29,44]:

*(i) Strength/Power*: Maximal efforts ≤10 s, primarily relying on the ATP–phosphocreatine system (e.g. vertical jump, medicine-ball throw, short sprint, kicking performance);

*(ii) Anaerobic Performance*: Efforts lasting >10 s and ≤60 s, predominantly glycolytic (e.g. Wingate test, repeated sprint ability, Taekwondo anaerobic intermittent kick test);

*(iii) Muscular Endurance*: Sustained submaximal contractions performed to volitional exhaustion or failure, typically involving repeated or continuous efforts targeting a specific muscle group, and generally lasting from ~30 seconds to several minutes depending on protocol (e.g. 2 × 30 s repeated jump, bench press to failure);

*(iv) Aerobic Endurance*: Continuous efforts exceeding ~2–3 minutes (typically > 10 minutes) where oxidative metabolism provides the predominant energy supply, even when significant anaerobic contributions occur in early phases (e.g. Yo-Yo intermittent recovery test, time-to-exhaustion cycling).

**6) Caffeine total exposure dose**: Following Nabuco et al., (2023) study [12], total oral exposure to caffeine during mouth rinsing was computed as:

Total dose(mg)=Concentration(%)×Volume(mL)×number of rinses×1000

This composite index reflects the overall caffeine mass theoretically available to stimulate oral receptors by integrating concentration (stimulus intensity), rinse volume (surface contact), and rinse frequency (stimulation repetitions), while minimising collinearity among these interrelated parameters.

To further address whether rinse frequency may exert effects beyond those captured by total caffeine exposure, we additionally examined the number of rinses as an independent moderator in exploratory sensitivity analyses, using both continuous and categorical specifications (single: 1; moderate: 2–9; high: ≥10 rinses).

**7) Rinse duration**: Rinsing time was analysed as a categorical moderator (5 s, 10 s, 15 s and 30 s), as it may modulate receptor activation independently of total caffeine exposure dose.

**8) Interaction term (total dose × duration):** To examine whether the ergogenic response depends on both the magnitude and duration of oral exposure, an exploratory multiplicative term between total dose and rinse duration (dose × duration) was tested in the meta-regression model.

Given the limited number of trials assessing cognitive performance (k = 7) and the heterogeneity of their outcome measures, we classified all outcomes into two measurement-based domains to enhance statistical comparability and reduce heterogeneity:

(i) Speed-Based Performance: This domain included outcomes measuring response or completion time (e.g. reaction times in hand/foot/kick tests, mirror-tracing time, Stroop/Simon task reaction times). For these measures, lower values indicate better performance. Effect sizes were therefore sign-adjusted so that a positive pooled effect consistently represents improved performance.
(ii) Accuracy-Based Performance: This domain included outcomes measuring response accuracy or error rate (e.g. accuracy percentage, error counts in Stroop/Simon tasks, mirror-tracing errors). For these measures, higher accuracy or fewer errors indicate better performance. Their effect direction already aligned with this convention and required no adjustment.

Other potential moderators (e.g. caffeine dose, rinse duration, participant sex, blinding quality) could not be examined quantitatively due to insufficient data.

Mixed-effects multilevel meta-regression models were fitted using restricted maximum likelihood (REML) estimation in the metafor package (rma.mv function), with individual effect sizes nested within studies and t-distribution–based inference. For continuous exploratory moderators, including total caffeine exposure dose, the dose × rinse duration interaction and rinse frequency, both linear and nonlinear (quadratic, cubic) specifications were examined. Model parsimony was determined by the corrected Akaike information criterion (AICc), and higher-order terms were retained only when they substantially improved model fit and were supported by sufficient data coverage [45,46].

All visualisations were generated using *ggplot2* and *orchaRd* packages [47].

#### 2.5.4. Publication bias and sensitivity analyses

Contour-enhanced funnel plots [48] and Egger’s regression tests [49] (when k ≥ 10) were used to assess publication bias [50].

Sensitivity analyses included: 1) varying assumed correlation coefficients (r): a relatively low value (r = 0.2) and a relatively high value (r = 0.8); 2) leave-one-out analyses; 3) exclusion of outliers identified via Cook’s distance and studentized residuals [51,52]; 4) exclusion of all single-blind studies to evaluate blinding effects on outcomes and 5) exclusion of studies that used rinse volumes other than 25 mL (if applicable), because deviations from this commonly used volume may alter oral contact area, perceived stimulus intensity, and receptor activation, potentially introducing additional heterogeneity. Among these, outlier exclusion was applied not only to the overall models but also across all moderator and meta-regression analyses to ensure robustness of subgroup inferences, whereas the remaining sensitivity cheques were conducted for the main pooled effects.

### 2.6. Certainty of the evidence

The certainty of evidence was evaluated using the Grading of Recommendations Assessment, Development, and Evaluation (GRADE) framework, considering risk of bias, inconsistency, indirectness, imprecision, and publication bias [53]. Ratings were categorised as high, moderate, low, or very low. All GRADE assessments were performed independently by one reviewer and verified by a second. Any disagreements were resolved through discussion until consensus was reached.

## 3. Results

### 3.1. Studies retrieved

**Figure 1.**
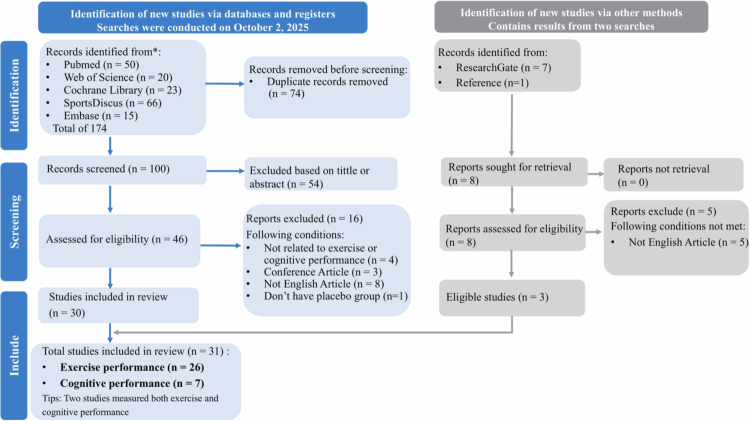
PRISMA flow diagram for included and excluded studies.

The initial search yielded 182 publications: 174 from the primary database search and 8 from other sources. After screening, a total of 31 studies met the inclusion criteria. These studies provided 167 effect size estimates (k = 167), of which 26 studies (k = 114) examined exercise performance and 7 studies (k = 53) examined cognitive performance (Figure 1). Two of these studies assessed both exercise and cognitive outcomes [19,54].

### 3.2. Characteristics of included studies

#### 3.2.1. Characteristics of exercise performance studies

Across all studies, a total of 384 participants were included (346 males, 38 females), with sample sizes ranging from 7 to 38. The majority of exercise performance studies recruited only males (*n* = 22, k = 83), four studies recruited mixed-sex samples (k = 31), and no study recruited exclusively female participants. For training level, 14 studies (k = 57) included trained participants with performance levels ranging from amateur to elite, while 12 studies (k = 57) examined untrained individuals. Furthermore, most studies did not report participants’ habitual caffeine intake (*n* = 14, k = 50). Among those that did, the majority primarily included low-caffeine consumers (*n* = 8, k = 55), whereas four studies involved participants with moderate caffeine intake (k = 8). Notably, only one study recruited a mixed sample that included high caffeine consumers (k = 1) [5].

Rinse duration ranged from 5 to 30 seconds. Most studies used 5 seconds (*n* = 8, k = 41) or 10 seconds (*n* = 15, k = 54), whereas a single study used 15 seconds (k = 6) and another used 30 seconds (k = 11). Notably, one study included two distinct rinse durations [5]. Almost all studies used 25 mL of Caff-MR, except for two that employed 50 mL [20,55]. The most common caffeine solution concentration was 1.2%, and the number of rinses varied from 1 to 22 depending on the experimental protocol.

According to the type of exercise categorised by the primary underlying energy system or neuromuscular mechanism, 14 studies investigated aerobic endurance (k = 25), 8 examined anaerobic performance (k = 28), 14 assessed strength/power (k = 32), and 5 evaluated muscular endurance (k = 29). Regarding pre-exercise nutritional status, 13 studies (k = 44) were conducted under fed conditions (≤4 hours after the last meal), 7 studies (k = 50) under fasted conditions (>4 hours), and 6 studies (k = 20) did not specify dietary status or stated it as habitual. Notably, one study directly compared the fed and fasted conditions [16].

For more details, please refer to Table 1.

**Table 1.** Summary and results of the studies reviewed assessing the effect of caffeine mouth rinsing on exercise performance.

Study; Study design	Exercise protocol	Sample；Training status	Mean age (y); Body weight (kg); Daily caffeine intake	Dosage of CAF and PLA; MR protocol (concentration: w/v); Dietary situation	Washout period	Performance outcomes	Statistical significance
Barbosa et al. [55] RDB Crossover	800 m running	7 males; Recreationally endurance-trained (running 19 ± 10 km/week)	24.6 ± 11.5; 78.2 ± 7.9; Moderate (150 mg/day);	CAF: 300 mg PLA: 300 mg microcrystalline cellulose; 50 mL: 1 × 10 s (0.6%): Pre-test; Maintain their regular diet	≥1 week	Time (s): CAF: 189.6 ± 30.4 vs PLA: 185.6 ± 30.3	Performance: No (*p* > 0.05)
Beaven et al. [7] RDB Crossover	5 × 6 s cycling sprints with 24 s rest between each	12 males; Recreationally trained (≥6 months)	32 ± 7.5; 82.2 ± 7.16; Low (≤2 doses of caffeinated beverages/day)	CAF: 300 × 5 mg + saccharin PLA: Calorie-free saccharin solution; 25 mL: 5 × 5 s (1.2%): Pre- and between each rest; 2 h postprandial	≥48 h	Peak power compared to PLA (W): Sprint 1-5: 21.43 ± 34.28, 13.13 ± 28.12, −2.42 ± 25.95, 8.30 ± 33.75, −12.86 ± 32.14 Mean power compared to PLA (W): Sprint 1: 26.93 ± 34.67, 34.93 ± 27.74, −21.87 ± 26.93, 11.20 ± 34.13, −7.47 ± 32.00	Performance: Yes Only with mean power of sprint 1 and 2 ↑ and sprint 3 ↓ (*p* < 0.05)
Boat et al. [56] RDB Crossover	Self-control exertion (S10) or non-self-control exertion (N10) 10 km cycling time trial test	15 males, Endurance recreationally trained cyclists (average training 8 ± 3 h/week)	22.4 ± 2.56; 78.7 ± 7.9; N/A	CAF: 200 mg PLA: Taste and colour matching; 25 mL: 5 × 10 s (0.16%): Pre- and every 2 km; 4 h postprandial	≥48 h	Overall time of S10 and N10 (s): CAF: 990 ± 89.08 and 986 ± 89.08 vs PLA: 996 ± 89.08 and 989 ± 92.95	Performance: No (*p* > 0.05)
Bottoms et al. [8] RDB Crossover	30 minutes self-pacing cycling	12 males; Recreationally trained cyclists	20.5 ± 0.7; 87.4 ± 18.3; N/A	CAF: 40 mg PLA: Water; 25 mL: 5 × 5 s (0.032%): Pre- and every 6 minutes; 4 h postprandial	1 week	Distance (km): CAF: 16.2 ± 2.8 vs PLA: 14.9 ± 2.6 Cadence (rev/min): CAF: 77.6 ± 13.6 vs PLA: 72.3 ± 12.5 Speed (km/h): CAF: 32.3 ± 5.9 vs PLA: 30.0 ± 5.4 Power output (W): CAF: 155.2 ± 27.5 vs PLA: 145.3 ± 23.5	Performance: Yes ↑ (*p* < 0.05)
Clarke et al. [57] RDB Crossover	1 RM bench press; 60% 1 RM bench press to exhaustion	15 males; Recreationally resistance-trained (training 3 ± 1 times/week)	21 ± 2; 77 ± 6; N/A	CAF: 300 mg + 200 mg sucralose PLA: Water + 200 mg sucralose; 25 mL: 1 × 10 s (1.2%): Pre-test; N/A	≥48 h	1 RM (kg): CAF: 87.26 ± 17.74 vs PLA: 86.16 ± 16.83 Number of repetitions (times): CAF: 22.01 ± 5.20 vs PLA: 21.92 ± 4.75 Total wight lifted (kg): CAF: 1143.90 ± 375.61 vs PLA: 1136.59 ± 326.82	Performance: No (*p* > 0.05)
Doering et al. [5] RDB Crossover	Task equal to 60 minutes cycling at 75% peak power	10 males; Well-trained male cyclists	32.9 ± 7.5; 74.7 ± 5.3; 6 high (320 ± 93 mg/day), 4 low (0 mg/day)	CAF: 280 mg + de-carbonated, non-caffeinated diet cola PLA: De-carbonated, non-caffeinated diet cola; 25 mL: 1 × 30 s + 7 × 10 s (0.14%): Pre- + every 12.5%; 1 h postprandial	1 week	Time (s): CAF: 3918 ± 243 vs PLA: 3940 ± 227 Difference of high and low caffeine consumer finish time between CAF and PLA (s): 2 ± 56 and 52 ± 25 Pre- and post-plasma caffeine concentration (μM): CAF: 1.2 ± 0.0 and 0.3 ± 0.0 vs PLA: 1.4 ± 0.0 and 0.1 ± 0.0	Performance: No (*p =* 0.23); Plasma caffeine concentration: No (*p* > 0.05)
Dolan et al. [58] RDB Crossover	Yo-Yo intermittent recovery test-Level 1	10 males; NCAA Division II competitive lacrosse players	19.9 ± 1.3; 84.9 ± 15.7; N/A	CAF: 300 mg + sucralose and nonsugar, cherry flavoured sweetener PLA: Sucralose and nonsugar, cherry flavoured sweetener; 25 mL: 1 × 10 s (1.2%): Pre-test; 10 h overnight fast	1 week	Distance (m): CAF: 1342 ± 320 vs PLA: 1397 ± 360	Performance: No (*p* > 0.05)
Farmani et al. [15] RDB Crossover	Throwing medicine ball; Sarjent’s jump test; Bruce test	18 males; Table tennis players (3 years of national league experience)	21.86 ± 2.40; 61.81 ± 10.32; N/A	CAF: Coffee, but total exposure is about (240 + 60) mg PLA: 5 g starch capsule; 25 mL: 5 × 5 s (0.24%): 4 times before power test + 1 time before Bruce test; 2 h postprandial	1 week	Throwing distance difference between CAF and PLA (m): 0.24 ± 0.83 Jump height difference between CAF and PLA (cm): CAF: 0.72 ± 4.54 VO_2max_ difference between CAF and PLA (mL/min/kg): CAF: −0.22 ± 4.22 TTE difference between CAF and PLA (minutes): 0.53 ± 0.85	Performance: Yes Ony on TTE ↑ (*p* < 0.05)
Figueiredo et al. [11] RDB Crossover	10 km running; Vertical jump	10 (8 males and 2 females); Moderate runners (5-6 times/weeks about 5-40 km)	30.1 ± 6.4; 68.8 ± 10.5; Overall moderate	CAF: 300 mg PLA: 300 mg microcrystalline cellulose; 25 mL: 1 × 10 s (1.2%): Pre-test; Maintain their regular diet	1 week	Times (s): CAF: 47.45 ± 6.34 vs PLA: 47.07 ± 5.18 Pre- and post- vertical jump relative power (W/kg): CAF: 4.4 ± 0.7 and 4.4 ± 0.8 vs PLA: 4.5 ± 0.6 and 4.5 ± 0.7	Performance: No (*p* > 0.05)
Gough et al. [10] RDB Crossover	Repeated sprint ability test	9 males; Recreational soccer players (training 12 ± 4 years)	21 ± 3; 68.0 ± 9.0; Low (about 70 mg/day)	CAF: 400 mg + sucralose PLA: sucralose; 25 mL: 1 × 10 s (1.6%): Pre-test; 2 h postprandial	≥96 h	Pre- and post-mean power (W): CAF: 217.37 ± 18.16 and 218.16 ± 25.79 vs PLA: 212.89 ± 27.37 and 222.37 ± 25.79 Pre- and post-peak power (W): CAF: 231.47 ± 22.97 and 230.59 ± 26.47 vs PLA: 217.06 ± 25 and 226.47 ± 25.29	Performance: No (*p* > 0.05)
Karayiğit et al. [59] RDB Crossover	30 s Wingate test	10 males; Healthy, physically active (join sport activities 8-10 h/week)	20.50 ± 1.58; 76.55 ± 5.38; N/A	CAF: 3000 mg + sodium saccharin PLA: Water + sodium saccharin 25 mL: 6 × 10 s (2%):1 pre- + every 60 s during warm-up + 1 times before power test; 10 h overnight fast	3–5 days	Mean power (W): CAF: 641.57 ± 77.53 vs PLA: 648.31 ± 74.16 Peak power (W): CAF: 944.94 ± 121.35 vs PLA: 968.54 ± 117.98	Performance: No (*p* > 0.05)
Karayigit, Ali et al. [54] RDB Crossover	1 RM squat and bench press; 3 × 40% 1RM squat and bench press to exhaustion	27 (13 males, 14 females); Healthy, resistance-trained team sport athletes (training 3 times/week)	Males: 24 ± 3; 84 ± 8; Females: 21 ± 1; 68 ± 6; Low (<25 mg/day)	CAF: 11000 mg + sucralose PLA: Water + sucralose; 25 mL: 22 × 10 s (2%):8 pre- + 1 × 2 times before 1 RM test + 6 × 2 times before endurance test; 12 h overnight fast	48–96 h	Male and female 1 RM bench press (kg): CAF: 102.35 ± 11.85 and 66.30 ± 5.3 vs PLA: 104.37 ± 11.85 and 65.80 ± 4.28 Male and female 1 RM squat (kg): CAF: 159.19 ± 14.37 and 98.46 ± 7.91 vs PLA: 156.31 ± 14.38 and 97.74 ± 8.26 Male and female 40% 1 RM bench press repetitions of set 1-3 (times): CAF: 33.21 ± 5.60, 24.76 ± 3.57, 15.48 ± 2.73 and 22.86 ± 2.62, 16.55 ± 2.62, 10.71 ± 2.62 vs PLA: 33.93 ± 4.4, 23.45 ± 2.86, 16.79 ± 2.85 and 21.90 ± 2.74, 16.90 ± 1.91, 12.14 ± 2.38 Male and female 40% 1 RM squat repetitions of set 1−3 (times): CAF: 42.76 ± 6.03, 31.03 ± 7.59, 19.83 ± 6.20 and 36.03 ± 6.38, 28.28 ± 6.55 and 15.86 ± 5.17 vs PLA: 41.03 ± 6.04, 31.55 ± 5.86, 20.52 ± 5.86 and 34.14 ± 6.03, 27.59 ± 5.69, 17.07 ± 3.96	Performance: No (*p* > 0.05)
Karayigit, Koz et al. [9] RDB Crossover	1 RM bench press; 3 × 60% 1RM bench press to exhaustion	14 males; Healthy, non-smoker resistance-trained (training 3 ± 1 years)	23 ± 2; 83 ± 4; Low (<12 mg/day)	CAF: (250 + 2 × 3 × 250/(500 + 2 × 3 × 500)/(750 + 2 × 3 × 750) mg + sucralose PLA: Water + sucralose; 25 mL: 7 × 5 s (1/2/3%): 1 time before 1 RM test + 3 × 2 times before endurance test; 10 h overnight fast	48 h	1 RM bench press (kg): 1%, 2%, 3% CAF: 96.31 ± 5.28, 97.39 ± 5.60, 97.49 ± 6.58 vs PLA: 97.92 ± 5.61 60% 1 RM bench press repetitions of set 1 (times): 1%, 2%, 3% CAF: 23.03 ± 4.55, 24.44 ± 4.85, 25.76 ± 5.05 vs PLA: 23.74 ± 5.35 60% 1 RM bench press repetitions of set 2 (times): 1%, 2%, 3% CAF: 18.99 ± 4.24, 19.49 ± 4.85, 20.40 ± 4.15 vs PLA: 19.60 ± 3.63 60% 1 RM bench press repetitions of set 3 (times): 1%, 2%, 3% CAF: 16.36 ± 5.05, 16.67 ± 4.74, 16.77 ± 5.25 vs PLA: 16.06 ± 3.64	Performance: Yes Only with 3% CAF vs PLA on repetitions ↑ (*p* < 0.05)
Karuk et al. [60] RSB Crossover	2 × 30 s vertical jump, with 5 minutes rest between each	8 males; Highly trained healthy athletes (national team members, training 6 h/day and 6 day/week for 9 years)	22.3 ± 4.2; 72.9 ± 8.8; Overall moderate	CAF: 300 mg PLA: saccharin, non-caloric sweetener; 25 mL: 1 × 10 s (1.2%): Pre-test; overnight fast	≥48 h	Maximal, mean, minimal jump height change from the first to the second test under CAF relative to PLA: −0.80 ± 4.73, −4.6 ± 8.0, −7.0 ± 12.9 Relative peak power change from the first to the second test under CAF relative to PLA: −1.6 ± 3.6	Performance: No (*p* > 0.05)
Kizzi et al. [61] RSB Crossover	5 × 6 s cycling sprints, with 24 s rest between each	8 males; Recreationally active	23 ± 2; 84 ± 4; N/A	CAF: 3000 mg PLA: Very strong sugar-free orange squash; 25 mL: 6 × 10 s (2%): 2 pre-and 1 between each sprint; 1 h postprandial	1 week	Peak power (W): CAF: 643 ± 79 vs PLA: 573 ± 79 Mean power (W): CAF: 589 ± 80 vs PLA: 519 ± 82	Performance: Yes ↑ (*p* < 0.05)
Marinho et al. [62] RDB Crossover	30 s Wingate test	10 males; Healthy	24.8 ± 3.7; 71.0 ± 7.8; N/A	CAF: 300 mg + calorie-free mint flavour PLA: Calorie-free mint flavour; 25 mL: 1 × 10 s (1.2%): Pre- test; 2 h postprandial	≥48 h	Peak power (W/kg): CAF: 15.05 ± 0.68 vs PLA: 14.99 ± 0.74 Mean power (W/kg): CAF: 12.28 ± 0.50 vs PLA: 11.96 ± 0.40	Performance: No (*p* > 0.05)
Marinho et al. [63] RDB Crossover	30 minutes constant load cycling to fatigue and then 10 km cycling time trial test	10 males; Healthy, physically active (having moderate-vigorous intensity exercise ≥ 150 minutes/week)	24.7 ± 3.6; 75 ± 9.4; Low (75.44 ± 51.51 mg/day)	CAF: 1200 mg + cellulose capsules PLA: cellulose capsules + magnesium sulphate solution; 25 mL: 4 × 10 s (1.2%): Pre- and middle of constant cycling and 10 km cycling; 2 h postprandial	3–7 days	Time (s): CAF: 1363 ± 345 vs PLA: 1321 ± 320 Mean power (W): CAF: 145 ± 12 vs PLA: 169 ± 29	Performance: No (*p* > 0.05)
Melo et al. [64] RSB Crossover	80% respiratory compensation point cycling until task failure	12 males; Healthy, physically active (with some cycling experience)	22.0 ± 2.8; 68.2 ± 12.2; N/A	CAF: 300 × (?) mg + non-caloric mint essence PLA: Non-caloric mint essence; 25 mL: (?) × 10 s (1.2%): pre- and every 15 minutes until exhaustion; 2 h postprandial	72–96 h	Exhaustion time (minutes): CAF: 91 ± 22 vs PLA: 76 ± 19	Performance: Yes ↑ (*p* < 0.05)
Miraftabi et al. [16] RDB Crossover	20 m sprit; Countermovement jump; Yo-Yo interval recovery running Level 1	13 males; Trained soccer players (training 5.15 ± 1.06 years)	18.1 ± 0.9; 60.1 ± 8.4; N/A	CAF: 6000 mg + non-caloric mint essence PLA: microcrystalline cellulose + sugar-free flavouring saccharin; 25 mL: 8 × 15 s (3%): pre-test, with 30 s interval between each rinse; 2 h postprandial or 9-10 h overnight fast	1 week	Fast and fed sprint time (s): CAF: 3.3 ± 0.5 and 3.45 ± 0.2 vs PLA: 3.4 ± 0.1 and 3.5 ± 0.1 Fast and fed jump height (cm): CAF: 38.95 ± 5.62 and 38.43 ± 5.53 vs PLA: 38.05 ± 4.79 and 38.08 ± 6.63 Fast and fed Yo-Yo test distance (m): CAF: 2155 ± 484 and 2098 ± 679 vs PLA: 1933 ± 549 and 1864 ± 535	Performance: Yes Only with CAF vs PLA on Yo-Yo test ↑ (*p* < 0.05)
Nabuco et al. (2021) RDB Crossover	75% peak power cycling to failure	10 males; Trained cyclists (cycling ≥ 200 km/week)	32 ± 3; 72.8 ± 5.3 Over moderate (232 ± 120 mg/day)	CAF: (?) × 85 mg PLA: Water; 25 mL: (?) × 5 s (0.34%): Pre-and every 5 minutes; Maintain their regular diet	1 week	Time (s): CAF: 2004 ± 767 vs PLA: 1688 ± 618	Performance: No (*p* > 0.05)
Pak et al. (2020) RDB Crossover	6 × Taekwondo anaerobic intermittent kick tests	27 (18 males, 9 females); Well-trained Taekwondo athletes	Male: 18 ± 4; 61.44 ± 11.45; Female: 16 ± 3; 54.22 ± 7.75; N/A	CAF: 6 mg/kg + artificial saccharine PLA: Water + artificial saccharine; 25 mL: 1 × 10 s (?%): Pre- test; ≥12 h Ramadan fast	N/A	Pre-, during (4 weeks) and post- Ramadan successful kicks (times): CAF: 40.4 ± 6.9, 36.6 ± 6.05, 39.0 ± 7.5, 39.3 ± 6.8, 40.0 ± 6.6 and 40.4 ± 6.1 vs PLA: 40.1 ± 6.9, 33.9 ± 5.8, 36.3 ± 7.0, 37.8 ± 6.7, 38.9 ± 6.6 and 40.1 ± 6.4 Pre-, during (4 weeks) and post- Ramadan total kicks (times): CAF: 52.1 ± 6.2, 49.7 ± 6.2, 50.2 ± 6.3, 50.1 ± 6.3, 51.1 ± 5.7 and 52.2 ± 6.1 vs PLA: 52.1 ± 6.2, 49.7 ± 6.6, 49.7 ± 6.1, 50.4 ± 6.2, 51.1 ± 6.0 and 52.3 ± 6.1	Yes Only with successful kicks times of first 3 weeks (*p* < 0.05)
Pataky et al. (2016) RDB Crossover	3 km cycling time trial	38 (25 males, 13 females); Recreationally trained cyclists (training ≥ 1 time/week)	21 ± 1; 70 ± 11; Over low (70 mg/day)	CAF: 600 mg + saccharine PLA: 2 × 6 g saccharine; 25 mL: 2 × 5 s (1.14%): before and 30 s at end of warm-up; 2 h postprandial	3–7 days	Mean power output percent difference between CAF and PLA (W): −0.17 ± 8.32	Performance: No (*p* > 0.05)
Şahin et al. (2024) RDB Crossover	10 × 6 s repeated cycling sprints	16 males; Karate athletes (training ≥ 4 time/week, ≥ 45 minutes/time)	21.6 ± 3.39; 77.9 ± 16.4; N/A	CAF: 300 mg + non-caloric sweetener PLA: Water + non-caloric sweetener; 25 mL: 1 × 10 s (1.2%): Pre-test; ≥3 h postprandial	≥48 h	Relative peak power and mean power (W/kg): CAF: 7.53 ± 0.88 and 5.90 ± 0.72 vs PLA: 7.45 ± 0.84 and 5.81 ± 0.70	Performance: No (*p* > 0.05)
Sinclair & Bottoms (2015) RSB Crossover	30 minutes arm crank time trial	12 males; Healthy	21.54 ± 1.28; 73.69 ± 5.40; N/A	CAF: 32 mg PLA: Water; 25 mL: 4 × 5 s (0.032%): every 6 minutes; 4 h postprandial	N/A	Distance (km): CAF: 15.43 ± 3.27 vs PLA: 13.15 ± 3.36 Ending power (W): CAF: 196.62 ± 70.95 vs PLA: 172.97 ± 58.79 Ending cadence (RPM): CAF: 97.85 ± 36 vs PLA: 86.46 ± 29.23 Ending RPE: CAF: 16.17 ± 2.30 vs PLA: 16.09 ± 1.87	Performance: Yes ↑ (*p* < 0.05)
Taheri Karami et al. (2023) RDB Crossover	Futsal intermittent endurance test; Sarjent’s jump test	24 males; Elite young futsal players	19.09 ± 1.57; 70.83 ± 11.46; N/A	CAF: Coffee, but total exposure is about (300 + 60)/(625 + 125) mg PLA: Water; 25 mL: 6 × 5 s (0.24/0.5%): 1 time before jump test + 1 time before and 3 during the endurance test + 1 before the last jump test; 2 h postprandial	1 week	Distance of endurance test (m): 0.24% and 0.5% CAF: 1494.4 ± 220 and 1677.7 ± 206.1 vs PLA: 1439.8 ± 236.3 Baseline, baseline_CAF and last jump height (cm): 0.24% CAF: 45.2 ± 4.2, 47.4 ± 3.3 and 49.3 ± 4.1 vs 0.5% CAF: 45.1 ± 3.6, 48.5 ± 3.2 and 51.1 ± 3.6 vs PLA: 44.7 ± 4.1, 46.4 ± 4.2 and 48.3 ± 4.1	Performance: Yes Only with 0.24% and 0.5% CAF vs PLA, and 0.5% CAF vs 0.24% CAF on distance ↑; 0.5% CAF vs 0.24% CAF or PLA on last jump height ↑ (*p* < 0.05)
Tallis et al. (2024) RDB Crossover	Countermovement jump; Drop jump; Isometric mid-thigh pull; 2 × 70% 1 RM chest press, shoulder press, deadlift and squat to failure	27 males; College football players	20 ± 2; 96.6 ± 18.2; Overall moderate: 188 ± 88 mg/day	CAF: 3 mg/kg + 20 mL water + 30 mL sugar-free orange drink PLA: 20 mL water + 30 mL sugar-free orange drink + sucralose; 50 mL: 1 × 30 s (?): Pre- test; Maintain their regular diet	3–5 days	Countermovement jump height (cm): CAF: 32.3 ± 6.9 vs PLA: 31.3 ± 5.6 Drop jump height (cm): CAF: 19.6 ± 6.7 vs PLA: 17.8 ± 7.3 Peak force of mid-thigh pull (W): CAF: 29.1 ± 4.8 vs PLA: 27.8 ± 4.8 Repetitions of chest press, shoulder press, deadlift and squat of set 1 (times): CAF: 15 ± 3, 12 ± 3, 14 ± 3 and 12 ± 4 vs PLA: 15 ± 3, 11 ± 3, 13 ± 3 and 12 ± 4 Repetitions of chest press, shoulder press, deadlift and squat of set 2 (times): CAF: 12 ± 2, 10 ± 3, 11 ± 4 and 10 ± 3 vs PLA: 12 ± 2, 9 ± 3, 11 ± 3 and 10 ± 4	Performance: No (*p* > 0.05)

**Notes**: ***CAF***: Caffeine; ***cm:*** Centimeters; ***h***: Hours; ***km***: Kilometre; ***kg:*** Kilogram; ***m*****:** Metre; ***mg*****:** Milligram**;*****mL:*** Millilitres; ***ms:*** Millisecond***; N/A:*** Not Available or Not Applicable; ***PLA:*** Placebo; ***RDB:*** Randomised double-blind trial; ***rev/min***: Revolutions per minute; ***RSB:*** Randomised single-blind trial; ***s:*** Seconds; ***μM***: micromolar; ***W***: Watt; ***w/v***: Weight per volume; ***y***: years old; ***↑***: Significantly increase; ***↓***: Significantly decrease; ***?***: Unclear/unfixed data.

#### 3.2.2. Characteristics of cognitive performance studies

Across all studies, a total of 217 participants were included (154 males and 63 females), with individual sample sizes ranging from 10 to 65. Among the seven cognitive studies, four recruited only male participants (k = 2), while three involved mixed-sex samples (k = 19), of which one reported pooled result without sex-specific analyses (k = 5). Regarding training status, only two studies (k = 10) involved trained participants, whereas five (k = 43) examined untrained individuals. In terms of habitual caffeine consumption, four studies recruited participants with moderate intake (k = 36), two with low intake (k = 13), and one study mentioned caffeine use without specifying the amount (k = 4).

In the seven cognitive studies, rinse duration was either 10 or 20 seconds. All used 25 mL of mouth rinse, and the number of rinses ranged from one to eight depending on the experimental protocol.

In terms of cognitive task type, seven studies involved speed-based performance (k = 32), and five examined accuracy-based performance (k = 21). With respect to pre-exercise nutritional status, three studies tested participants in the fed state (k = 35), two in the fasted state (k = 14), and one did not specify dietary status (k = 4).

For more details, please refer to Table 2.

**Table 2.** Summary and results of the studies reviewed assessing the effect of caffeine mouth rinsing on cognitive performance.

Study; Study design	Exercise protocol	Sample；Training status	Mean age (y); Body weight (kg); Daily caffeine intake	Dosage of CAF and PLA; MR protocol (concentration: w/v); Dietary situation	Washout period	Performance outcomes	Statistical significance
Balcı et al. [18] RDB Crossover	Victorias Stroop test (Part D and W for reaction time, Part C for response inhibition)	30 males; Healthy, recreationally active	22.7 ± 3.3; 75.6 ± 12.8; Moderate (150-300 mg/day);	CAF: 60/150/300 mg + sugar-free orange flavour PLA: Water + sugar-free orange flavour; 25 mL: 1 × 20 s (0.24/0.6/1.2%): Between the pre- and post- test; 2 h postprandial	1 week	Pre- and post- Part D reaction time (s): Male: 0.24% CAF: 58.95 ± 19.36 and 58.23 ± 15.91 vs 0.6% CAF: 63.53 ± 21.22 and 59.15 ± 19.00 vs 1.2% CAF: 62.93 ± 19.07 and 57.01 ± 16.74 vs PLA: 61.95 ± 19.57 and 61.58 ± 15.93 Pre- and post- Part W reaction time (s): Male: 0.24% CAF: 58.56 ± 17.54 and 57.82 ± 14.61 vs 0.6% CAF: 59.98 ± 14.20 and 58.28 ± 14.61 vs 1.2% CAF: 59.60 ± 16.08 and 55.87 ± 15.93 vs PLA: 58.90 ± 13.03 and 62.23 ± 15.19 Pre- and post- Part C reaction time (s): Male: 0.24% CAF: 63.70 ± 19.28 and 62.83 ± 15.21 vs 0.6% CAF: 66.69 ± 19.14 and 62.70 ± 19.46 vs 1.2% CAF: 67.31 ± 17.32 and 60.65 ± 16.37 vs PLA: 62.29 ± 17.44 and 69.57 ± 21.06 Pre- and post- Part W error rate (%): Male: 0.24% CAF: 6.43 ± 5.50 and 5.70 ± 5.50 vs 0.6% CAF: 7.00 ± 6.21 and 5.63 ± 5.76 vs 1.2% CAF: 5.70 ± 4.23 and 5.10 ± 4.09 vs PLA: 6.00 ± 5.80 and 6.00 ± 5.07 Pre- and post- Part C error rate (%): Male: 0.24% CAF: 6.80 ± 6.06 and 7.20 ± 7.56 vs 0.6% CAF: 7.67 ± 6.64 and 6.53 ± 6.50 vs 1.2% CAF: 5.90 ± 5.07 and 4.97 ± 4.00 vs PLA: 6.73 ± 5.30 and 7.03 ± 5.95 Pre- and post- Part D error rate (s): Male: 0.24% CAF: 7.77 ± 6.58 and 8.53 ± 8.48 vs 0.6% CAF: 8.77 ± 8.80 and 7.10 ± 7.11 vs 1.2% CAF: 7.83 ± 6.06 and 6.73 ± 5.10 vs PLA: 6.30 ± 5.23 and 8.03 ± 6.91	Performance: Yes Only with pre- vs post-test of 1.2% CAF on Part D reaction time ↓; Pre- vs post- test of 0.6% CAF on Part D and C error rate ↓; (*p* < 0.05)
De Pauw et al. [6] RDB Crossover	Stroop task	10 males; Healthy	27 ± 3; 73.8 ± 7.6; Overall moderate (about two doses of caffeinated beverages/day)	CAF: 300 mg + sodium salt of saccharin PLA: Unflavoured artificial saliva + sodium salt of saccharin; 25 mL: 1 × 20 s (1.2%): Pre- test; Fast (visit the laboratory with an empty belly)	N/A	Pre- and post- of congruent reaction time (ms): CAF: 630.3 ± 119.9 and 605.1 ± 85.2 vs PLA: 599.8 ± 56.5 and 597.8 ± 66.7 Pre- and post- of incongruent reaction time (ms): CAF: 715.2 ± 125.9 and 674.0 ± 103.6 vs PLA: 651.1 ± 67.4 and 659.2 ± 80.1 Pre- and post- of simple reaction time (ms): CAF: 595.0 ± 50.8 and 606.2 ± 74.4 vs PLA: 590.5 ± 68.3 and 623.6 ± 84.2 Pre- and post- of congruent accuracy rate (%): CAF: 95.0 ± 5.3 and 95.3 ± 3.8 vs PLA: 94.8 ± 5.9 and 94.2 ± 5.2 Pre- and post- of incongruent accuracy rate (%): CAF: 93.6 ± 5.6 and 95.0 ± 3.4 vs PLA: 95.8 ± 5.0 and 95.0 ± 4.9 Pre- and post- of simple accuracy rate (%): CAF: 95.9 ± 3.8 and 95.9 ± 3.8 vs PLA: 94.6 ± 3.8 and 95.6 ± 3.5	Performance: No (*p* > 0.05)
Karayigit, Ali, et al. [54] RDB Crossover	Modified arrow flanker task	27 (13 males, 14 females); Healthy, resistance-trained team sport athletes (training 3 times/week)	Males: 24 ± 3; 84 ± 8; Females: 21 ± 1; 68 ± 6; Low (< 25 mg/day)	CAF: 4000 mg + sucralose PLA: Water + sucralose; 25 mL: 8 × 10 s (2%): 8 times during the pre- and post-test; 12 h overnight fast	48-96 h	Pre- and post- congruent accuracy (%): Male: CAF: 95.60 ± 1.8 and 96.78 ± 2.3 vs PLA: 96.86 ± 1.9 and 96.72 ± 2.4; Female: CAF: 96.37 ± 1.2 and 95.49 ± 1.9 vs PLA: 95.90 ± 1.5 and 96.13 ± 2.2 Pre- and post- incongruent accuracy (%): Male: CAF: 92.49 ± 2.4 and 93.20 ± 2.3 vs PLA: 93.82 ± 1.8 and 93.54 ± 2.6; Female: 94.52 ± 3.3 and 94.82 ± 2.7 vs PLA: 94.63 ± 2.4 and 93.99 ± 2.1 Pre- and post- congruent reaction time (ms): Male: CAF: 460.08 ± 54.1 and 465.95 ± 42.0 vs PLA: 463.86 ± 38.8 and 461.91 ± 42.4; Female: 524.31 ± 30.5 and 512.93 ± 39.8 vs PLA: 531.67 ± 48.6 and 529.10 ± 41.3 Pre- and post- incongruent reaction time (ms): Male: CAF: 485.92 ± 40.8 and 489.03 ± 38.6 vs PLA: 504.32 ± 55.9 and 509.20 ± 51.8l; Female: 535.70 ± 48.8 and 528.31 ± 40.9 vs PLA: 538.43 ± 43.3 and 536.80 ± 41.2	Performance: No (*p* > 0.05)
Pomportes et al. [65] RSB Crossover	Duration-production task and Simon task during 40 minutes 60% peak power cycling	24 (16 males, 8 females); Physically active (training 3-8 h/week)	Males: 24 ± 6; 79 ± 11; Females: 30 ± 10; 57 ± 4; Low (< 200 mg/day)	CAF: 201 mg + orange sugar-free syrup PLA: Water + orange sugar-free syrup; 25 mL: 3 × 20 s (0.268%): 1 time before and 2 times during the cycling; 3 h postprandial	≥72 h	Overall variance of duration production task (ms): CAF: 180.7 ± 39.98 vs PLA: 195.0 ± 60.80 Overall rection time of congruent and incongruent Simon task (ms): CAF: 334.4 ± 25.20 and 359.1 ± 24.10 vs PLA: 331.4 ± 25.20 and 361.1 ± 27.93 Overall error rate of congruent and incongruent Simon task (%): CAF: 4.5 ± 2.74 and 9.8 ± 4.38 vs PLA: 4.5 ± 2.19 and 9.1 ± 3.83	Performance: Yes Only with variance of duration production ↓ (*p* < 0.05)
Şahin et al. [19] RDB Crossover	Kick reaction time; Hand reaction time	16 males; karate athletes (training ≥ 4 time/week, ≥ 45 minutes/time)	21.6 ± 3.39; 77.9 ± 16.4; N/A	CAF: 300 mg + non-caloric sweetener PLA: Water + non-caloric sweetener; 25 mL: 1 × 10 s (1.2%): Pre-test; ≥ 3 h postprandial	≥48 h	Kicking reaction time of set 1 and 2 (ms): CAF: 475.1 ± 76.5 and 422.0 ± 67.4 vs PLA: 435.4 ± 63.4 and 429.6 ± 55.5 Hand reaction time of set 1 and 2 (ms): CAF: 465.6 ± 80.5 and 424.1 ± 45.3 vs PLA: 448.5 ± 55.0 and 434.5 ± 43.9	Performance: Yes Only with v Kicking reaction time of set 1 ↓ (*p* < 0.05)
Toktaş et al. [21] RSB Crossover	Stroop colour-word test; Mirror-tracing test	65 (24 males and 41 females); Healthy, recreationally active	Male: 29.91 ± 12.06; 74.41 ± 10.83; Female: 22.89 ± 3.94; 56.75 ± 8.81 Overall moderate (50–300 mg/day)	CAF: Coffee, but total exposure is about 32.5 mg PLA: Water; 25 mL: 1 × 10 s (0.13%): Between pre- and post- test; 2 h postprandial	≥1 week	Pre- and post- Stroop test reaction time of male (s): CAF: 24.92 ± 14.20 and 21.00 ± 6.02 vs PLA: 23.00 ± 7.83 and 21.92 ± 5.39 Pre- and post- Stroop test reaction time of female (s): CAF: 21.02 ± 6.49 and 20.93 ± 6.22 vs PLA: 19.90 ± 6.83 and 19.76 ± 6.34 Pre- and post- mirror-tracing test reaction time of male (s): CAF: 46.87 ± 26.96 and 36.79 ± 21.72 vs PLA: 53.29 ± 36.51 and 40.67 ± 21.40 Pre- and post- mirror-tracing test reaction time of female (s): CAF: 34.12 ± 19.92 and 28.49 ± 12.33 vs PLA: 32.17 ± 24.09 and 29.56 ± 20.92 Pre- and post- mirror-tracing test error number of male (times): CAF: 12.83 ± 20.16 and 10.04 ± 21.08 vs PLA: 9.88 ± 19.41 and 5.88 ± 9.11 Pre- and post- mirror-tracing test error number of female (times): CAF: 6.20 ± 10.33 and 4.49 ± 6.33 vs PLA: 6.41 ± 9.48 and 6.54 ± 10.81	Performance: No (*p* > 0.05)
Virdinli et al. [66] RDB Crossover	Hand reaction time test; Foot reaction time test	45 males; Healthy trained volleyball or football athletes (training 9 ± 3 years and 14 ± 3 h/week)	18 ± 3; N/A; Overall moderate (163 mg/day)	CAF: 300/450/600 mg PLA: Water; 25 mL: 1 × 10 s (1.2/1.8/2.4%): Pre- test; ≥ 2 h postprandial	≥3 days	Hand reaction time (ms): 1.2% CAF: 393.57 ± 52.14 vs 1.8% CAF: 411.43 ± 54.28 vs 2.4% CAF: 362.86 ± 35.71 vs PLA: 462.86 ± 82.85 Foot reaction time (ms): 1.2% CAF: 364.93 ± 56.71 vs 1.8% CAF: 379.10 ± 49.26 vs 2.4% CAF: 317.16 ± 38.81 vs PLA: 403.73 ± 63.43	Performance: Yes All reaction time of CAF groups vs PLA ↓, and 2.4% CAF < 1.2/1.8% CAF (*p* < 0.05)

**Notes**: ***CAF***: Caffeine; ***h***: Hours; ***kg:*** Kilogram; ***mg*****:** Milligram**;*****mL:*** Millilitres; ***ms:*** Millisecond***; N/A:*** Not Available or Not Applicable; ***PLA:*** Placebo; ***RDB:*** Randomised double-blind trial; ***RSB:*** Randomised single-blind trial; ***s:*** Seconds; ***w/v***: Weight per volume; ***y***: years old; ***↓***: Significant decrease.

### 3.3. Primary analysis

**Figure 2.**
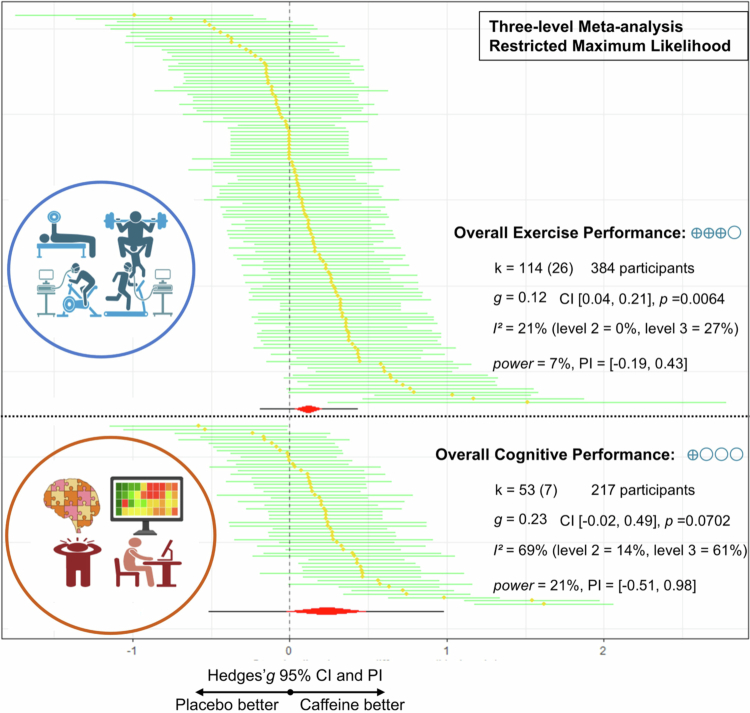
Primary pooled effect sizes for caffeine mouth rinse on overall exercise and cognitive performance. **Notes:** ***K***, the total number of effects included in the pooled effect size; ***Hedge's g***, the effect size indicators used in the pooled; ***95%CI***, 95% confidence interval; ***95%PIE***, prediction Interval; ***P-value***, statistically significant *P* values ​​for pooled results; ***I***^**2**^, quantitative indicators of heterogeneity; ***Power***, statistical power for pooled effect size; ***Blue circles***, Grade, grading of recommendations assessment, development, and evaluation, a system for evaluating the quality of evidence and strength of recommendations.

Our meta-analysis showed that Caff-MR may be associated with trivial improvements in general exercise performance outcomes (k = 114, g = 0.12, 95% CI [0.04, 0.21], I^2^ = 21% [low], PIE [−0.19, 0.43], *p* = 0.006, Moderate GRADE) (Figure 2; see also Appendix S3 for a traditional forest plot with study labels and weights).

For general cognitive performance, the primary meta-analysis did not show consistent benefits of Caff-MR (k = 53, g = 0.23, 95% CI [−0.02, 0.49], I^2^ = 69% [substantial], PIE [−0.51, 0.98], *p* = 0.07, Very low GRADE) (Figure 2; see also Appendix S3 for a traditional forest plot with study labels and weights).

Variance decomposition from the three-level models indicated that sampling error (level 1) accounted for 73% of the total variance in exercise performance and 25% in cognitive performance (Figure 2). Following Hunter & Schmidt (1990) recommendation [67], when the proportion of total variance attributable to sampling error is <75%, meaningful between-study heterogeneity is likely present. Accordingly, we proceeded to moderator analyses.

### 3.4. Moderator analysis

#### 3.4.1. Moderator analysis of exercise performance

Moderator analyses indicated that in male participants, Caff-MR may be associated with trivial improvements in exercise performance (k = 91, g = 0.14, 95% CI [0.04, 0.23], I² = 28% [moderate], PIE [−0.19, 0.47], *p* = 0.01; GRADE: Moderate), whereas no consistent enhancement was found in the female subgroup (k = 8, g = 0.11, 95% CI [−0.15, 0.37], *p* = 0.42; GRADE: Very Low). The between-group difference by sex was not statistically significant (*p* = 0.76, I² = 21%).

Similarly, Caff-MR produced a trivial improvement in untrained participants (k = 57; g = 0.15; 95% CI [0.01, 0.28]; I² = 31% [moderate]; PIE [−0.21, 0.51]; *p* = 0.04; GRADE: High) but showed no consistent ergogenic effect in trained participants (k = 8, g = 0.11, 95% CI [−0.15, 0.37], *p* = 0.42; GRADE: Very Low). The difference between subgroups was not significant (*p* = 0.62; overall I² = 21%). Across strata of habitual caffeine intake (high, medium, and low), Caff-MR did not yield a consistent ergogenic effect. By contrast, participants with unclear intake exhibited a clear and consistent performance benefit (k = 50; g = 0.23; 95% CI [0.13, 0.32]; I² = 0% [low]; 95% PIE [0.01, 0.44]; *p* < 0.01; GRADE: Very low), with the effect size significantly greater than that observed in the low- and medium-intake strata (*p* < 0.05).

Caff-MR was associated with a small improvement in exercise performance under fed conditions (k = 44; g = 0.22; 95% CI [0.12, 0.32]; I² = 48% [moderate]; 95% PIE [−0.01, 0.44]; *p* < 0.01; GRADE: Low). In contrast, under fasting conditions the effect was near zero and imprecise (k = 50; g = 0.01; 95% CI [−0.10, 0.12]; *p* = 0.84; GRADE: Low). The fed-state effect was significantly greater than the fasting-state effect (*p* < 0.05).

Across exercise types, Caff-MR demonstrated a consistent ergogenic effect on aerobic endurance performance (k = 25; g = 0.21; 95% CI [0.01, 0.35]; I² = 41% [moderate]; 95% PIE [−0.12, 0.54]; *p* < 0.01; GRADE: Moderate). By contrast, effects on anaerobic performance, muscular endurance, and strength/power were inconsistent and not statistically significant (all *p* > 0.05), and no between-group differences were detected (interaction *p* > 0.05).

Interestingly, mouth rinsing for 10, 15, or 30 seconds did not yield a consistent ergogenic effect, whereas the shortest duration of 5 seconds did (k = 41; g = 0.23; 95% CI [0.09, 0.36]; I² = 39% [moderate]; 95% PIE [−0.08, 0.53]; *p* < 0.01; GRADE: Low). Furthermore, the 5-second effect size was significantly greater than that observed with 10 seconds (*p* < 0.05).

**Figure 3.**
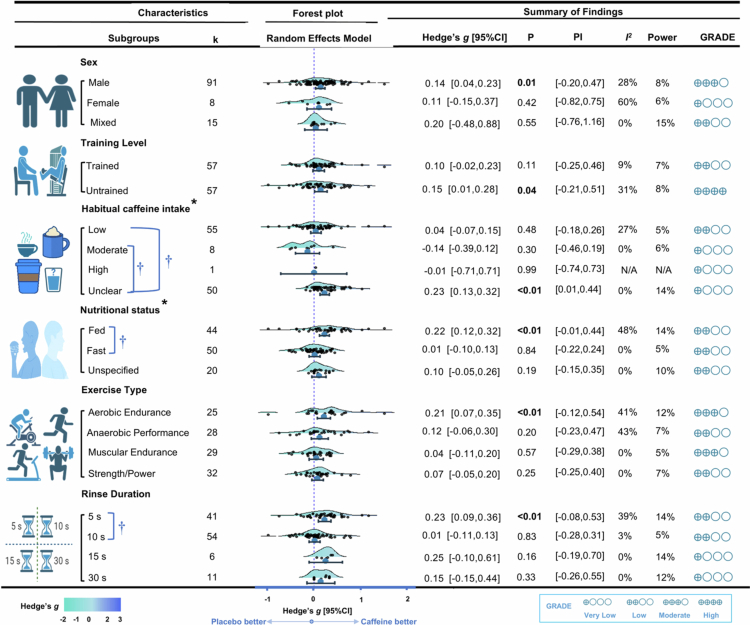
Moderator analysis for exercise primary results. **Notes:** ***K***, the total number of effects included in the pooled effect size; ***Hedge's g***, the effect size indicators used in the pooled; ***95%CI***, 95% confidence interval; ***95%PIE***, prediction Interval; ***P-value***, statistically significant *P* values ​​for pooled results; ***I***^**2**^, quantitative indicators of heterogeneity; ***Power***, statistical power for pooled effect size; ***GRADE,*** grading of recommendations assessment, development, and evaluation, a system for evaluating the quality of evidence and strength of recommendations; *, represents significant differences between groups; †, represents significant difference between the two categories within the group.

For more information on exercise performance subgroup results, please refer to Figure 3.

After harmonisation, five studies [20,54,64,68,69] were excluded from the dose–response analysis due to incomplete or inconsistent dosing information (for example, doses reported only in mg·kg⁻¹, missing concentration data, or inconsistent rinse counts relative to the target exposure window). Given the wide range of total exposures, we performed a meta-regression on log₁₀-transformed total caffeine exposure and found no linear association with performance (*β* = −0.02, *p* = 0.79) (Appendix S4).

However, a quadratic model indicated a significant U-shaped relationship (β₁ = −1.34, *p* = 0.02; β₂ = 0.24, *p* = 0.03). Based on this model, the earliest point at which a stable ergogenic effect emerged was at log₁₀(dose) = 1.05, corresponding to the lowest included total exposure of 32 mg, after which the effect size diminished with increasing dose and reached a nadir at the turning point log₁₀(dose) = 2.81 (≈646 mg). The dose window supporting a stable ergogenic effect was approximately 32–133 mg (Appendix S5). A cubic specification provided a poorer fit and yielded no significant terms (Appendix S6).

Exploratory analyses further examined rinse frequency as an independent moderator. When modelled categorically, moderate rinse frequencies (2–9 rinses) were associated with a small improvement in exercise performance (k = 51, g = 0.17, 95% CI [0.06, 0.28], *p* < 0.01), and this pattern persisted after exclusion of outliers (k = 39, g = 0.25, 95% CI [0.13, 0.36], *p* < 0.01). In contrast, single-rinse and high-frequency protocols did not show statistically significant benefits (*p* > 0.05). When rinse frequency was treated as a continuous variable, no significant linear association with performance was observed. However, quadratic meta-regression identified a statistically significant inverted U-shaped association (β₁ = 0.04, *p* = 0.01; β₂ = −0.01, *p* = 0.01), suggesting that ergogenic effects may be more detectable at intermediate rinse frequencies, with attenuated effects at lower and higher frequencies. For more details, please refer to Appendix S7.

Finally, in the exploratory linear model relating rinse time **×** total exposure, rinse time was treated as a categorical factor (5 s vs 10 s) and total caffeine exposure as a continuous predictor, with dose centred at 32 mg so that the intercept reflects the minimum exposure. There was no significant linear association between dose and performance within either the 5-second or 10-second conditions, and no evidence that the dose–response differed between these conditions (Appendix S8). The 30-second condition was excluded due to unclear total exposure, and the 15-second condition could not be examined in the interaction model because it appeared in only one study with insufficient variation in exposure [16].

#### 3.4.2. Moderator analysis of cognitive performance

Moderator analyses indicated that Caff-MR did not consistently enhance performance on either speed-based cognitive tasks (k = 32; g = 0.26; 95% CI [−0.01, 0.51]; *p* = 0.051; GRADE: Very low) or accuracy-based tasks (k = 21; g = 0.19; 95% CI [−0.08, 0.47]; *p* = 0.16; GRADE: Low). No significant between-task differences were detected (*p* = 0.43; I² = 67%). For more information on cognitive performance subgroup results, please refer to Appendix S9.

### 3.5. Risk of bias and quality of methods

Across exercise outcomes, no study was rated overall “low risk”. Several studies were judged as “high risk” [8,15,70], with the remainder classified as having “some concerns.” At the domain level, risk of bias was generally low for missing outcome data and outcome measurement, whereas concerns were more common for aspects related to randomisation, reporting practices, and deviations from intended interventions. Assessments of bias arising from period and carryover effects were mixed, with many studies rated as having some concerns and the remainder as low risk. Overall, the exercise evidence base reflects a predominantly moderate risk-of-bias profile, with high-risk judgments primarily attributable to insufficient reporting of random sequence generation and allocation concealment, unclear or absent pre-registration and specification of primary outcomes, and limited detail to verify adherence to the intended mouth-rinse intervention (Appendix S10).

For cognitive performance, similarly, no study achieved an overall “low risk” rating. One study was assessed as “high risk” [21], with the remainder judged as having “some concerns.” Across domains, most studies showed low risk for missing outcome data, while concerns were more frequent for reporting practices, randomisation-related procedures, and deviations from intended interventions. Bias arising from period and carryover effects was generally low, with some concerns noted in a minority of studies. Taken together, the cognitive evidence indicates a predominantly moderate risk-of-bias landscape with isolated high-risk assessments (Appendix S10).

To improve methodological quality, future studies should adopt transparent and well-documented randomisation procedures, pre-register study protocols with clearly defined primary outcomes, and report intervention fidelity in detail, particularly with respect to mouth-rinse volume, duration, and washout control. Adherence to established reporting guidelines for randomised crossover trials may further reduce risk-of-bias concerns and strengthen internal validity.

Funnel plots, together with Egger’s regression, indicated no significant publication bias for exercise performance (*p* = 0.10), whereas cognitive performance showed significant asymmetry (*p* < 0.01) (Appendix S11). Median statistical power was low in both domains (7.3% for exercise; 26.4% for cognition), and the R-index suggested poor replicability (3.3% and 26.3%, respectively). These diagnostic results suggest the need for cautious interpretation of pooled effects (Appendix S12).

In the moderator analyses, Egger’s regression was applied to all subgroups with at least 10 studies (k ≥ 10). For exercise outcomes, significant funnel-plot asymmetry was detected for subgroups with unclear habitual caffeine intake, for both fed and fasted pre-test nutritional states, and for the 5-second mouth-rinsing duration (all *p* < 0.05), whereas no evidence of publication bias was observed in the remaining subgroups. For cognitive outcomes, the speed-based performance subgroup showed evidence of asymmetry (*p* < 0.05). Further details are provided in Appendix S11.

For exercise outcomes, the mean modified PEDro score was 7.3, consistent with good methodological quality. For cognitive outcomes, the mean score was 6.6, indicating fair methodological quality. More information, please refer to Appendix S13. In addition, the certainty of evidence for each outcome was evaluated using the GRADE approach, with details provided in Appendix S14.

### 3.6. Sensitivity analysis

#### 3.6.1. Sensitivity analysis for primary effect

For exercise performance, results were generally robust across sensitivity analyses. In the between-study (level-3) leave-one-out analysis, exclusion of the Taheri Karami et al. (2023) study [17] yielded a modest shift in the pooled estimate that was borderline non-significant (g = 0.11, *p* = 0.052) and eliminated detectable heterogeneity (I² = 0%). All other sensitivity cheques did not materially affect the direction or magnitude of the pooled effect (Appendix S15 and S16).

For cognitive performance, assuming r = 0.8 and after excluding statistical outliers, Caff-MR showed a significant ergogenic effect (*p* < 0.05) (Appendix S15). In the level-3 leave-one-out analysis, exclusion of the Virdinli et al. study [66] reduced heterogeneity from substantial to 0% (Appendix S16).

#### 3.6.2. Sensitivity analysis for moderator effect

Within the three-level framework, outlier diagnostics substantially altered several moderator findings. After removing influential cases, several exercise-based subgroups were excluded due to insufficient data: the female subgroup (sex), the medium- and high-intake strata (habitual caffeine intake), and the 15-s rinsing-duration stratum. Additionally, three subgroups that were non-significant in the primary analysis became statistically significant: the trained subgroup (g = 0.13; k = 51; *p* = 0.01), the unspecified feeding-state subgroup (g = 0.12; k = 19; *p* = 0.02), and the strength/power subgroup (g = 0.13; k = 29; *p* = 0.01) (Appendix S17). The linear dose–response relationship remained non-significant (Appendix S4), while the previously observed quadratic, non-linear dose–response was no longer evident (Appendix S6). By contrast, the dose × time analysis indicated that a stable negative linear association emerged between total exposure and performance under the 5-s condition (*β* = −0.18; k = 63; *p* = 0.02) (Appendix S8).

For cognitive outcomes, after excluding outliers, Caff-MR showed a robust ergogenic effect on speed-based tasks (Appendix S17).

## 4. Discussion

In contrast to previous meta-analytic conclusions of a nonsignificant overall effect [12], the present synthesis demonstrates that Caff-MR can provide small but measurable benefits for exercise performance, with more context-dependent effects on cognitive outcomes. These results indicate that the effects of Caff-MR are not uniform, but vary according to how central perception and motivation interact with task demands and dosing features. The following sections therefore examine how participant characteristics, exercise and cognitive task type, nutritional status, and rinsing strategy influence the detectability and practical relevance of Caff-MR effects.

### 4.1. Exercise outcomes

#### 4.1.1. Participant characteristics

Across sex, our primary models suggested a trivial, directionally positive effect in men, whereas women showed no consistent benefit. The sex-by-subgroup contrast was not significant. The field lacks female-specific evidence. No study enroled an exclusively female cohort, and the available estimates for women come from subgroup reports within mixed-sex samples with very low statistical power [54]. Furthermore, they did not report on menstrual cycle control or hormonal contraception, which have been shown to potentially influence increases in perceptual and performance endpoints [3,71]. Mechanistically, Caff-MR is expected to act through oropharyngeal chemosensation and rapid engagement of reward and motor networks such as the insula, orbitofrontal cortex, and striatum, processes that are unlikely to be sex-limited [6,72]. Taken together, the absence of a consistent effect in women most likely reflects limited statistical power and design limitations rather than a true lack of responsiveness.

Untrained participants showed more consistent trivial gains, whereas effects in trained participants were of similar magnitude but less stable. After outlier removal, the trained subgroup effect became statistically robust. Two factors likely account for the initial instability. Biologically, trained athletes begin closer to performance ceilings and display lower within-person variability [3], which may limit the headroom for the motivation-related central effects hypothesised for Caff-MR to translate into measurable improvements. Methodologically, studies in this area are generally small, and those that recruit trained cohorts are smaller still [5,58,60]. Small samples increase the leverage of individual studies on pooled estimates and make signals appear unstable even in the absence of publication bias [26]. Beyond mouth rinse, findings from the broader caffeine literature also suggest that training status seldom acts as a reliable moderator for resistance-type outcomes [73–75]. This pattern supports a cautious interpretation that the apparent trained–untrained differences in our dataset are more sensitive to sampling and design features than to large, systematic physiological moderation by training status.

Regarding habitual caffeine intake, effects diverged by stratum. Both moderate and high consumers showed slightly negative estimates. However, evidence is sparse for moderate and high consumers. Only one dataset classified as high intake came from a subgroup report with 6 participants [5], so any inference is unstable. Interestingly, studies with unclear intake showed a clear positive effect with a prediction interval that did not include zero. Because a mouth rinse does not depend on systemic absorption from current evidence, classic pharmacological tolerance cannot fully explain this pattern. Instead, two explanations are more plausible. First, habitual use and expectancy may blunt central drive even without systemic exposure. Parts of the ingestion literature report attenuated acute benefits in heavy users, consistent with habituation of adenosinergic and motivational pathways [76,77]. Second, the “unclear” category likely reflects reporting and selection artifacts. Poor dietary reporting often co-occurs with other methodological limitations, and funnel-plot asymmetry was observed in related subgroups, both of which may inflate apparent effects [3,12]. Notably, for low-intake participants, the pooled effect clustered around zero which is positive but imprecise. While this is currently difficult to explain at the physiological level, it should not be interpreted as evidence of no effect, especially given that most studies only reported the mean of habitual intake within the group, a practice that can lead to significant misclassification errors [3,76].

Taken together, the apparent moderating patterns across sex, training status, and habitual caffeine use should be interpreted cautiously. Given that the overall effects of Caff-MR are small and subgroup estimates are inconsistent, differences in detectability rather than true biological moderation are a more parsimonious explanation. In this context, practical factors such as baseline performance level, expectancy-related central drive, and study design characteristics may disproportionately influence whether small effects are observed. These patterns therefore warrant confirmation in targeted, adequately powered studies.

#### 4.1.2. Pre-exercise nutritional status

The popularity of mouth rinse is largely due to its ability to bypass the gastrointestinal tract, thereby avoiding the delayed absorption and gastrointestinal discomfort that may accompany ingestion, while activating the central nervous system [3,5]. In practice, however, most athletes are not truly fasted before performance; they typically arrive fed to maximise substrate availability [43]. Distinguishing fed from fasted testing is therefore crucial for interpreting real-world relevance.

Consistent with this context, our subgroup analysis showed that fed-state testing was associated with a small improvement, whereas the fasted-state effect was near zero and imprecise, and the fed effect was significantly greater than the fasted effect.

A plausible mechanism is that, in the fed state, brief oropharyngeal stimulation may enhance central drive via insular, orbitofrontal, and striatal pathways at a time when metabolic substrates are readily available. Under these conditions, sufficient carbohydrate availability and habitual feeding patterns may allow small centrally mediated cues to translate into more stable regulation of pacing and perceived effort during sustained exercise [6,72]. In contrast, under fasting conditions, substrate availability may be reduced and perceived exertion tends to rise, particularly in individuals who are not accustomed to fasted exercise. These factors can increase interoceptive strain and variability in effort regulation, thereby masking subtle centrally mediated benefits of Caff-MR [43,78].

After outlier exclusion, the unspecified feeding-state subgroup showed a stable ergogenic signal. This likely reflects incomplete reporting in studies that instructed participants to maintain their habitual diet. Many such studies probably tested participants within 4 hours of a meal but without precise documentation of meal timing. Consequently, participants’ physiological state may have been closer to the fed condition rather than true fasting, which could increase the likelihood of detecting performance benefits.

However, the role of nutritional status remains unclear. One recent randomised trial reported similar improvements in a running task irrespective of feeding status [16], whereas our pooled analysis showed that fed testing yielded larger effects than fasted testing on average. These divergent findings suggest that nutritional status may interact with task characteristics and protocol details to influence effect detectability. Therefore, targeted studies are needed to clarify these relationships.

#### 4.1.3. Exercise type

Our analysis indicates that Caff-MR is most reliable for aerobic endurance, with a small but consistent benefit, whereas effects for anaerobic performance, muscular endurance, and strength/power were inconsistent overall. This divergent pattern can be explained by the differing role of central versus peripheral factors across exercise modalities.

During prolonged aerobic tasks (typically > 3 min), performance is largely governed by pacing strategies, attentional focus, and effort perception, which are amenable to central modulation [79–81]. Caff-MR operates through brief oropharyngeal stimulation of brain networks regulating motivation and perceived exertion [72], with neurophysiological evidence showing rapid activation of insular and orbitofrontal cortices [6,78]. Importantly, most aerobic endurance protocols in this review applied repeated mouth-rinse exposures during exercise, with only three studies relying on a single rinse [11,55,58]. Under such conditions, transient centrally mediated signals may be periodically refreshed, providing sufficient opportunity to influence pacing decisions in real time. Over the course of prolonged exercise, even small reductions in perceived effort may therefore accumulate into measurable performance gains.

Conversely, maximal strength and power efforts (around 5-10 s) depend almost exclusively on immediate ATP-phosphocreatine availability and explosive motor unit recruitment [82], leaving minimal opportunity for cognitive-perceptual modulation to enhance peak output [1,83]. Notably, after outlier exclusion, we detected a stable trivial effect (g = 0.11) in strength/power outcomes, suggesting a small yet reliable central contribution (e.g. enhanced neural drive or reduced pre-motor inhibition) that becomes apparent when methodological noise is controlled.

Anaerobic performance (10–60 s) represents a transitional zone where glycolytic capacity and effort regulation both contribute [83]. In brief single-bout efforts (<30 s), peripheral metabolic constraints (pH decline, phosphocreatine depletion) likely dominate, limiting Caff-MR's central influence. In repeated-bout protocols, central cues may modestly aid effort maintenance across sets, though our evidence suggests this effect remains small and variable. Similarly, muscular endurance tasks showed equivocal results, as performance is primarily constrained by local muscle fatigue and metabolite accumulation rather than central pacing [84,85].

Collectively, these findings support a task-dependency model wherein Caff-MR efficacy is maximal when performance relies on sustained effort regulation (aerobic endurance) and minimal when outputs are peripherally dominated (strength/power, short anaerobic efforts), extending prior observations with quantitative evidence across modalities [12,14].

#### 4.1.4. Dosing strategy

Our synthesis indicates that shorter rinse durations (~5 s) are more likely to yield reproducible, trivial-to-small ergogenic effects than 10–30 s, though the limited number of studies at 15 and 30 s warrants cautious interpretation. Increasing total oral exposure does not produce a linear improvement. In primary models, we observed a non-linear pattern in which benefits emerged at relatively low exposure and attenuated as exposure increased. However, this pattern lost robustness after outlier handling. Moreover, within the 5 s stratum, higher total exposure was negatively associated with performance, arguing against a simple “more or longer is better” assumption."

Mechanistically, a short oropharyngeal chemosensory pulse may sufficiently activate bitter and trigeminal receptors to transiently recruit insula–orbitofrontal–striatal networks implicated in motivation and effort regulation, providing rapid centrally mediated facilitation independent of systemic pharmacokinetics [6,72]. On the contrary, prolonged or excessive bitter or chemesthetic stimulation risks perceptual adaptation or crossing aversive thresholds, which can blunt motivational gain and offset benefits [43]. In addition, timing the rinse immediately before key pacing or force-production phases may strengthen perception action coupling, whereas spreading stimulation across longer windows may dilute attentional engagement and reduce the functional impact of the cue [86].

Within this framework, rinse frequency becomes relevant because it determines how often this transient signal is refreshed during exercise. In our exploratory moderator analyses, intermediate rinse frequencies were more consistently associated with detectable ergogenic effects, whereas both single rinse and very high frequency protocols showed less reliable benefits. This interpretation is consistent with the aerobic endurance subgroup, which predominantly used repeated rinses and showed a stable benefit overall, while the three endurance studies that relied on a single rinse did not report clear performance improvements [11,55,58]. At the other extreme, very frequent rinsing may interrupt pacing continuity or attentional focus and introduce task-level interference that counteracts potential central facilitation. Taken together, these findings suggest that the performance impact of Caff-MR is shaped not only by stimulus intensity and duration, but also by how often brief chemosensory pulses are delivered across the exercise bout.

Two included studies that explicitly examined dose response reported greater benefits with higher doses [9,17]. These findings can be interpreted within the same context-dependent framework rather than as evidence for a uniformly positive dose response. Tallis et al. used a 25 mL coffee-based rinse [17], which may have produced a different sensory profile than a pure caffeine solution, so palatability, caffeine preference, or interindividual variability in taste perception or adenosine-related sensitivity could have contributed beyond concentration alone. Karayigit et al. observed benefits only at an extreme concentration of 3 percent for muscular endurance [9]. When expressed as total oral exposure, it corresponds to the ascending limb of the upper range of our U-shaped dose–response curve, beyond the turning point (≈646 mg). Although pooled estimates did not show a stable effect in this high exposure region, the Karayigit et al. result suggests that very high exposures may confer benefits in specific contexts [9], potentially shaped by task demands, timing of repeated exposures, sensory habituation, or participant characteristics. Notably, both studies involved multiple rinses, further supporting the view that apparent dose effects may reflect protocol structure as much as total caffeine exposure.

Collectively, these data support a conservative strategy for Caff-MR: favour ~5-s rinses delivering low-to-moderate total oral exposure, avoid escalating dose or duration in pursuit of larger effects, and when repeated exposures are used, prioritise brief pulses aligned with decision-relevant phases rather than prolonged or highly disruptive rinsing schedules.

### 4.2 Cognitive outcomes

In the primary analysis, Caff-MR did not yield a stable effect on overall cognition or on either subgroup (speed- or accuracy-based). After removing outliers, the overall cognitive effect became significant, and the speed-based subgroup also reached significance, suggesting that Caff-MR may preferentially facilitate processing speed rather than accuracy.

This pattern is mechanistically plausible. Brief oropharyngeal chemosensory stimulation can rapidly engage insula, orbitofrontal, and anterior cingulate networks, leading to transient increases in cortical arousal and attentional control [6,72]. Such effects are well suited to shortening response latency, whereas accuracy typically depends on more sustained executive control and inhibitory processes that may be less responsive to brief sensory stimulation [87]. Consistent with this interpretation, mouth-rinse studies and the broader caffeine literature more often report faster responses or preserved processing speed under cognitive load or fatigue, while effects on accuracy tend to be smaller and more variable [54,88,89]. Together, the evidence supports context-dependent cognitive benefits with the greatest sensitivity in speed-based tasks, while underscoring the need for standardised, adequately powered designs to confirm durability and boundary conditions of these effects.

### 4.3 Future directions

Building on the present synthesis, several priorities should guide future work.

First, dosing strategy requires purpose-built, adequately powered, preregistered trials that orthogonally vary total oral exposure and rinse duration, with standardised reporting of concentration, volume, frequency, and sensory characteristics. Trials should pre-specify correlation handling for crossover designs and include blinding cheques to reduce expectancy confounds.

Second, future studies should directly test how exercise type affects Caff-MR outcomes. Trials that systematically vary exercise type alongside dosing parameters are needed to clarify when centrally mediated effects translate into meaningful performance gains, particularly in endurance tasks where pacing and effort perception are critical. Cognitive outcomes should be assessed using separate reaction time and accuracy metrics to better isolate domain-specific sensitivity.

Third, combined mouth-rinse strategies warrant further investigation. Existing evidence suggests that carbohydrate–caffeine [90,91] and menthol–caffeine [92] formulations may produce additive or synergistic effects through complementary sensory pathways. Future studies should evaluate these and other multi-ingredient combinations using tightly controlled designs that standardise sensory profiles, verify taste-matching procedures, and incorporate affective measures to determine whether perceptual and emotional pathways contribute to performance outcomes.

Fourth, pre-exercise nutritional status requires systematic investigation. Although our analyses suggest more stable effects under fed conditions, direct comparisons between fed and fasted testing remain scarce and yield inconsistent findings [16]. Future trials should explicitly control and report preprandial nutritional status to determine whether feeding state represents a true physiological moderator and to identify the contextual factors governing these interactions.

Fifth, because Caff-MR bypasses gastrointestinal transit and aims for rapid central engagement, intranasal caffeine merits exploratory investigation as a mechanistically adjacent route. Existing reviews highlight the theoretical potential of caffeinated nasal sprays to stimulate cranial nerve pathways and permit mucosal absorption [93]. However, evidence remains limited, as the only two studies have not demonstrated meaningful improvements in exercise or cognitive performance, nor detectable increases in systemic caffeine concentrations [94,95]. Moreover, apart from electrophysiological findings, neuroimaging data and direct assessments linking central activation to functional performance outcomes are lacking. Accordingly, early-phase studies should prioritise safety, pharmacokinetics, central nervous system markers, and head-to-head comparisons with mouth rinsing, followed by pragmatic performance trials and combination protocols to test for additive effects.

Sixth, chemical and formulation constraints should be made explicit. Caffeine’s water solubility at room temperature is approximately 20 mg·mL⁻¹, which equates to a practical ceiling near 2% in a 25 mL rinse [96,97]. One included study reportedly used 3% without full disclosure of preparation methods [9], which introduces potential bias and reproducibility risks. Future reports should detail solvent systems, temperature, pH, and stabilisers, and should verify concentration analytically to ensure methodological transparency and cross-study comparability.

Seventh, to date, only one study has measured post–mouth-rinse blood caffeine and reported no meaningful increase [5]. Future trials should incorporate standardised pharmacokinetic sampling (pre-exercise baseline and serial draws within 5–30 min), alongside central readouts (e.g. EEG/ERP or fNIRS), to further confirm that observed effects are centrally mediated rather than systemic and to refine dose–timing recommendations.

Finally, several populations warrant targeted investigation due to specific physiological and practical factors that may modify Caff-MR responses. Future trials should recruit and prospectively stratify women with controlled menstrual-cycle phase or hormonal contraceptive use, as sex hormones may influence central sensitivity, perceptual responses, and variability in performance outcomes. Highly trained athletes represent another priority group because they operate closer to performance ceilings, where small centrally mediated effects may be harder to detect yet practically meaningful. Habitual high-caffeine users also merit focused study, given potential differences in expectancy, tolerance-related neural adaptation, and receptor responsiveness that may modify responses to non-ingestive caffeine strategies. In addition, teenagers or older adults remain largely unexplored populations in whom caffeine metabolism, sensory processing, and central responsiveness may differ from young adults. In parallel, genotype should be a prespecified moderator. The only genotyped study to date classified runners by CYP1A2 and found no performance differences with a 1.2% rinse, but the sample was small and predominantly composed of C-allele carriers, limiting inference [11]. Future work should therefore include ADORA2A, CYP1A2, TAS2R, and exploratory COMT variants to test whether genetic susceptibility shapes perceptual salience and central effects independent of systemic exposure.

### 4.4 Strengths and limitations

This review provides the most comprehensive and methodologically rigorous synthesis of Caff-MR effects to date. We searched six databases, applied duplicate screening and extraction, and used a three-level meta-analytic framework that accounts for multiple outcomes within studies and avoids double counting. We quantified prediction intervals, conducted robust sensitivity analyses including outlier diagnostics and correlation assumptions for crossover designs, and evaluated evidence certainty via GRADE, which improves the transparency and decision utility of our conclusions.

Several limitations warrant consideration. First, despite the three-level approach, heterogeneity remained for multiple outcomes, reflecting variation in participants, dosing protocols, sensory properties, timing, and tasks. Second, the female evidence base is sparse, and most trained cohorts were small, which limits precision and generalisability. Third, age was not systematically restricted or analysed in our synthesis, which may obscure age-related differences in sensory perception, expectancy, or central responsiveness. Fourth, dose-reporting was inconsistent across primary studies. Several trials lacked complete concentration or rinse-frequency data, which constrained our dose–response modelling and required exclusions after harmonisation. Fifth, the estimates for 15 s and 30 s rinse durations rely on very few studies, so inferences for these durations are not robust. Sixth, analyses treating rinse frequency as an independent moderator were exploratory and should be interpreted cautiously. The categorical cut points were data driven and may not map cleanly onto practical protocols, and rinse frequency is tightly coupled to task design, duration, and exercise modality, which reduces interpretability as a stand-alone dose parameter. Finally, cognitive outcomes exhibited funnel-plot asymmetry and low statistical power, raising the possibility of small-study effects. These limitations highlight the need for caution in interpreting our findings and underscore the importance of high-quality, standardised research in this area.

### 4.5 Practical implications

Caff-MR may be considered a situational ergogenic aid rather than a universal performance strategy. Based on the present findings, several practical implications can be drawn for athletes, coaches, and sport scientists:

First, Caff-MR appears most relevant for aerobic endurance tasks, where pacing and effort regulation play a central role. Practitioners may consider its use during prolonged or self-paced endurance exercise, particularly when athletes are tested or compete in a fed state, which better reflects real-world practice.

Second, brief rinsing durations and low-to-moderate total exposure appear sufficient. Exploratory dose mapping suggests that very short rinses of approximately 5 seconds can elicit comparable benefits to longer protocols, while higher total exposure is unlikely to provide additional advantage. From a practical standpoint, this favours simple, time-efficient protocols that minimise disruption to exercise rhythm.

Third, the ergogenic benefit of Caff-MR is modest and context-dependent. Athletes and coaches should view Caff-MR as an optional adjunct rather than a primary ergogenic strategy. It may be particularly relevant as an alternative when conventional caffeine ingestion is impractical or poorly tolerated due to gastrointestinal discomfort or individual sensitivity.

Finally, applications for cognitive enhancement should be approached cautiously. Although some evidence suggests sensitivity of processing speed to Caff-MR, effects on cognitive accuracy and broader executive outcomes remain inconsistent. Sport scientists integrating Caff-MR into cognitive or dual-task training should therefore temper expectations and consider it exploratory rather than established practice.

Taken together, these findings support the selective and context-aware use of Caff-MR within endurance-focused training or competition settings, while highlighting the need for individualised decision-making and further protocol refinement.

## 5. Conclusion

Although the effect is small, current evidence indicates that Caff-MR provides modest but reliable performance benefits, predominantly in aerobic endurance tasks. Effects appear optimised with brief (~5 s) rinses and low-to-moderate total exposure, while cognitive findings are mixed but show greater sensitivity for processing speed after outlier removal. Given heterogeneity, limited female/trained-athlete data, and methodological variability, high-quality, well-powered trials with standardised dosing and detailed reporting are needed to confirm reliability and refine practical protocols.

## 6. Perspective

This meta-analysis offers the most comprehensive evaluation to date of Caff-MRas a rapid, ingestion-free ergogenic strategy. Our findings highlight small but meaningful benefits, particularly in aerobic endurance and processing speed, and show that these effects can be achieved with brief (~5 s) rinses and moderate total exposure, without the need for systemic absorption. Building on prior work in Caff-MR [12–14], this study reinforces the relevance of central, oropharyngeal mechanisms and positions Caff-MR as a practical option for athletes seeking performance support when ingestion is impractical or undesirable. At the same time, the results underscore the need for standardised protocols, better reporting, and sex- and training-specific trials to refine dosing, timing, and combined rinse strategies within sport and exercise science.

## Supplementary Material

Supplementary Material JISSN_258198509_R2_Electronic_Supplementary_Material

## Data Availability

All data analysed in this study were obtained from previously published studies, which are cited in the manuscript. No new data were generated for this study.
