# Supplementary material for: Revisiting the evidence on caffeine mouth rinse: effects on exercise and cognitive performance: a meta-analytic review
Source: J Int Soc Sports Nutr. 2026 Mar 3;23(1):2638903. doi: 10.1080/15502783.2026.2638903 (PMC12958389; doi:10.1080/15502783.2026.2638903)
Supplement: Supplementary Material — JISSN_258198509_R2_Electronic_Supplementary_Material [file RSSN_A_2638903_SM5378.docx]

**Electronic Supplementary Material Contents**

| **Number** | **Material** | **Page** |
| --- | --- | --- |
| 1 | Electronic Supplementary Material Appendix S1 (PRISMA 2020 checklist) | P3-6 |
| 2 | Electronic Supplementary Material Appendix S2 (Table. Summary of effect size calculation procedures) | P7-8 |
| 3 | Electronic Supplementary Material Appendix S3 (Summary forest plot of aggregated study effects: Exercise and Cognition) | P9-10 |
| 4 | Electronic Supplementary Material Appendix S4 (Simple linear regression analysis of caffeine mouth rinse total exposure dose) | P11 |
| 5 | Electronic Supplementary Material Appendix S5 (Quadratic meta regression analysis of caffeine mouth rinse total exposure dose) | P12 |
| 6 | Electronic Supplementary Material Appendix S6 (Total Exposure Dose Meta-Regression Analysis: Original Data and After Excluding Outliers) | P13-14 |
| 7 | Electronic Supplementary Material Appendix S7 (Exploratory Analysis of Rinse Frequency/Times: Original Data and After Excluding Outliers) | P15-16 |
| 8 | Electronic Supplementary Material Appendix S8 (Exploratory linear regression analysis of total exposure dose and rinsing time) | P17 |
| 9 | Electronic Supplementary Material Appendix S9 (Moderator analysis for cognitive primary results) | P18 |
| 10 | Electronic Supplementary Material Appendix S10 (ROB2 Assessment Tool for Risk of Bias: Exercise and Cognition) | P19-20 |
| 11 | Electronic Supplementary Material Appendix S11 (Funnel Plot: Exercise and Cognition) | P21-24 |
| 12 | Electronic Supplementary Material Appendix S12 (Power Visualization: Exercise and Cognition) | P25-28 |
| 13 | Electronic Supplementary Material Appendix S13 (PEDro Assessment: Exercise and Cognition) | P29-31 |
| 14 | Electronic Supplementary Material Appendix S14 (GRADE Assessment: Exercise and Cognition) | P32-35 |
| 15 | Electronic Supplementary Material Appendix S15 (Sensitivity Analysis for Primary results: Exercise and Cognition) | P36-37 |
| 16 | Electronic Supplementary Material Appendix S16 (A Sensitivity Analysis based on level 2 and level 3 Leave-one-out: Exercise and Cognition) | P38-41 |
| 17 | Electronic Supplementary Material Appendix S17 (Moderator Analysis After Excluding Outliers: Exercise and Cognitive performance) | P42-43 |

**Electronic Supplementary Material Appendix S1**

| **Section and Topic** | **Item #** | **Checklist item** | **Location where item is reported (on page)** |
| --- | --- | --- | --- |
| **TITLE : Revisiting the Evidence on Caffeine Mouth Rinse: Effects on Exercise and Cognitive Performance: A Meta-Analytic Review** | | |  |
| Title | 1 | Identify the report as a systematic review. | P1 |
| **ABSTRACT** | | |  |
| Abstract | 2 | See the PRISMA 2020 for Abstracts checklist. | P4-6 |
| **INTRODUCTION** | | |  |
| Rationale | 3 | Describe the rationale for the review in the context of existing knowledge. | P8-9 |
| Objectives | 4 | Provide an explicit statement of the objective(s) or question(s) the review addresses. | P9 |
| **METHODS** | | |  |
| Eligibility criteria | 5 | Specify the inclusion and exclusion criteria for the review and how studies were grouped for the syntheses. | P10 |
| Information sources | 6 | Specify all databases, registers, websites, organisations, reference lists and other sources searched or consulted to identify studies. Specify the date when each source was last searched or consulted. | P10 |
| Search strategy | 7 | Present the full search strategies for all databases, registers and websites, including any filters and limits used. | P10 |
| Selection process | 8 | Specify the methods used to decide whether a study met the inclusion criteria of the review, including how many reviewers screened each record and each report retrieved, whether they worked independently, and if applicable, details of automation tools used in the process. | P10-11 |
| Data collection process | 9 | Specify the methods used to collect data from reports, including how many reviewers collected data from each report, whether they worked independently, any processes for obtaining or confirming data from study investigators, and if applicable, details of automation tools used in the process. | P10-11 |
| Data items | 10a | List and define all outcomes for which data were sought. Specify whether all results that were compatible with each outcome domain in each study were sought (e.g. for all measures, time points, analyses), and if not, the methods used to decide which results to collect. | P11 |
|  | 10b | List and define all other variables for which data were sought (e.g. participant and intervention characteristics, funding sources). Describe any assumptions made about any missing or unclear information. | P11 |
| Study risk of bias assessment | 11 | Specify the methods used to assess risk of bias in the included studies, including details of the tool(s) used, how many reviewers assessed each study and whether they worked independently, and if applicable, details of automation tools used in the process. | P11 |
| Effect measures | 12 | Specify for each outcome the effect measure(s) (e.g. risk ratio, mean difference) used in the synthesis or presentation of results. | P12 |
| Synthesis methods | 13a | Describe the processes used to decide which studies were eligible for each synthesis (e.g. tabulating the study intervention characteristics and comparing against the planned groups for each synthesis (item #5)). | P12-13 |
|  | 13b | Describe any methods required to prepare the data for presentation or synthesis, such as handling of missing summary statistics, or data conversions. | P12-13 |
|  | 13c | Describe any methods used to tabulate or visually display results of individual studies and syntheses. | P12-13,16 |
|  | 13d | Describe any methods used to synthesize results and provide a rationale for the choice(s). If meta-analysis was performed, describe the model(s), method(s) to identify the presence and extent of statistical heterogeneity, and software package(s) used. | P12-13 |
|  | 13e | Describe any methods used to explore possible causes of heterogeneity among study results (e.g. subgroup analysis, meta-regression). | P13-16 |
|  | 13f | Describe any sensitivity analyses conducted to assess robustness of the synthesized results. | P16-17 |
| Reporting bias assessment | 14 | Describe any methods used to assess risk of bias due to missing results in a synthesis (arising from reporting biases). | P16-17 |
| Certainty assessment | 15 | Describe any methods used to assess certainty (or confidence) in the body of evidence for an outcome. | P17 |
| **RESULTS** | | |  |
| Study selection | 16a | Describe the results of the search and selection process, from the number of records identified in the search to the number of studies included in the review, ideally using a flow diagram. | P17 |
|  | 16b | Cite studies that might appear to meet the inclusion criteria, but which were excluded, and explain why they were excluded. | P17-18 |
| Study characteristics | 17 | Cite each included study and present its characteristics. | P18-19 |
| Risk of bias in studies | 18 | Present assessments of risk of bias for each included study. | P23-24 |
| Results of individual studies | 19 | For all outcomes, present, for each study: (a) summary statistics for each group (where appropriate) and (b) an effect estimate and its precision (e.g. confidence/credible interval), ideally using structured tables or plots. | P20-23 |
| Results of syntheses | 20a | For each synthesis, briefly summarise the characteristics and risk of bias among contributing studies. | P18-25 |
|  | 20b | Present results of all statistical syntheses conducted. If meta-analysis was done, present for each the summary estimate and its precision (e.g. confidence/credible interval) and measures of statistical heterogeneity. If comparing groups, describe the direction of the effect. | P20-23 |
|  | 20c | Present results of all investigations of possible causes of heterogeneity among study results. | P20-23，25-26 |
|  | 20d | Present results of all sensitivity analyses conducted to assess the robustness of the synthesized results. | P25-26 |
| Reporting biases | 21 | Present assessments of risk of bias due to missing results (arising from reporting biases) for each synthesis assessed. | P23-25 |
| Certainty of evidence | 22 | Present assessments of certainty (or confidence) in the body of evidence for each outcome assessed. | P25 |
| **DISCUSSION** | | |  |
| Discussion | 23a | Provide a general interpretation of the results in the context of other evidence. | P26-34 |
|  | 23b | Discuss any limitations of the evidence included in the review. | P36-37 |
|  | 23c | Discuss any limitations of the review processes used. | P36-37 |
|  | 23d | Discuss implications of the results for practice, policy, and future research. | P34-36 |
| **OTHER INFORMATION** | | |  |
| Registration and protocol | 24a | Provide registration information for the review, including register name and registration number, or state that the review was not registered. | P9 |
|  | 24b | Indicate where the review protocol can be accessed, or state that a protocol was not prepared. | P9 |
|  | 24c | Describe and explain any amendments to information provided at registration or in the protocol. | N/A |
| Support | 25 | Describe sources of financial or non-financial support for the review, and the role of the funders or sponsors in the review. | P2 |
| Competing interests | 26 | Declare any competing interests of review authors. | P2 |
| Availability of data, code and other materials | 27 | Report which of the following are publicly available and where they can be found: template data collection forms; data extracted from included studies; data used for all analyses; analytic code; any other materials used in the review. | N/A |

**Electronic Supplementary Material Appendix S2 (Table. Summary of effect size calculation procedures)**

| Step | Data condition | Calculation Formula | Description |
| --- | --- | --- | --- |
| ① | Pre–post data available | $\Delta CAF= M_{\mathrm{CAF}\mathrm{post}}- M_{\mathrm{CAFpre}}$  $\Delta PLA= M_{\mathrm{PLA}\mathrm{post}}- M_{\mathrm{PLApre}}$  $\mathrm{MD}= \Delta CAF- \Delta PLA$ | Calculate the mean change within each condition (CAF-MR and placebo) and use their difference (MD) as the comparison metric. |
| ② | Only post-intervention data available | $\mathrm{MD}= M_{\mathrm{CAF}}- M_{\mathrm{PLA}}$ | When only post values are reported, the mean difference between conditions is used directly. |
| ③ | SD of change (for pre–post data) | $\begin{aligned} \mathrm{SD}_{\mathrm{change}}=\sqrt{{\mathrm{SD}^{2}}_{\mathrm{pre}}+{\mathrm{SD}^{2}}_{\mathrm{post}}-\left（ 2\times r\times\mathrm{SD}_{\mathrm{pre}}\times\mathrm{SD}_{\mathrm{post}} \right）} \end{aligned}$ | Computes the within-subject SD of change using the correlation (r) between pre- and post-measurements. |
| ④ | Pooled SD (for crossover design) | $\mathrm{SD}_{\mathrm{pooled}}=\sqrt{\frac{{\mathrm{SD}^{2}}_{\mathrm{CAF}}+{\mathrm{SD}^{2}}_{\mathrm{PLA}}}{2}}$ | Estimates the pooled variability across both conditions. |
| ⑤ | Effect size (Hedges’ *g*) | $Hedge’s g= \frac{M_{\mathrm{CAF}}- M_{\mathrm{PLA}}}{\mathrm{SD}_{\mathrm{pooled}}}\times\left( 1-\frac{3}{4\left( N-1 \right)-1} \right)$ | Standardized mean difference corrected for small-sample bias. |
| ⑥ | Standard error (SE) of *g* | $SE=\sqrt{\frac{1}{N}+\frac{g^{2}}{2N}}\times\sqrt{2\left( 1-r \right)}$ | Adjusts SE to account for within-subject correlation between CAF-MR and placebo conditions. |
| ⑦ | Assumed correlation (*r*) | *r* = 0.50 (primary analysis)  *r* = 0.20 and 0.80 (sensitivity) | The same r value was used for both pre–post and between-condition comparisons to ensure consistency and comparability. |

**Electronic Supplementary Material Appendix S3 (Summary forest plot of aggregated study effects: Exercise and Cognition)**


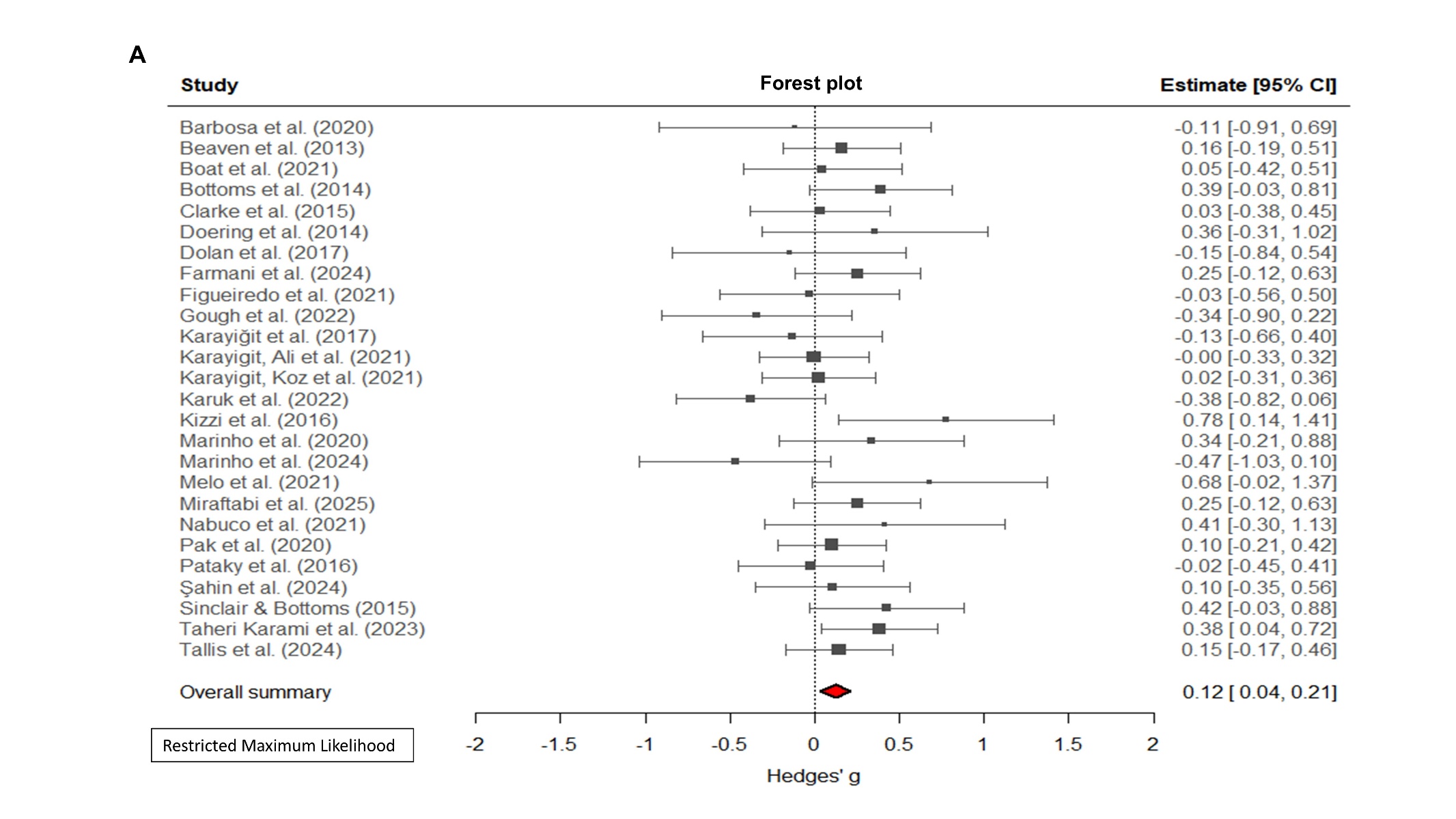

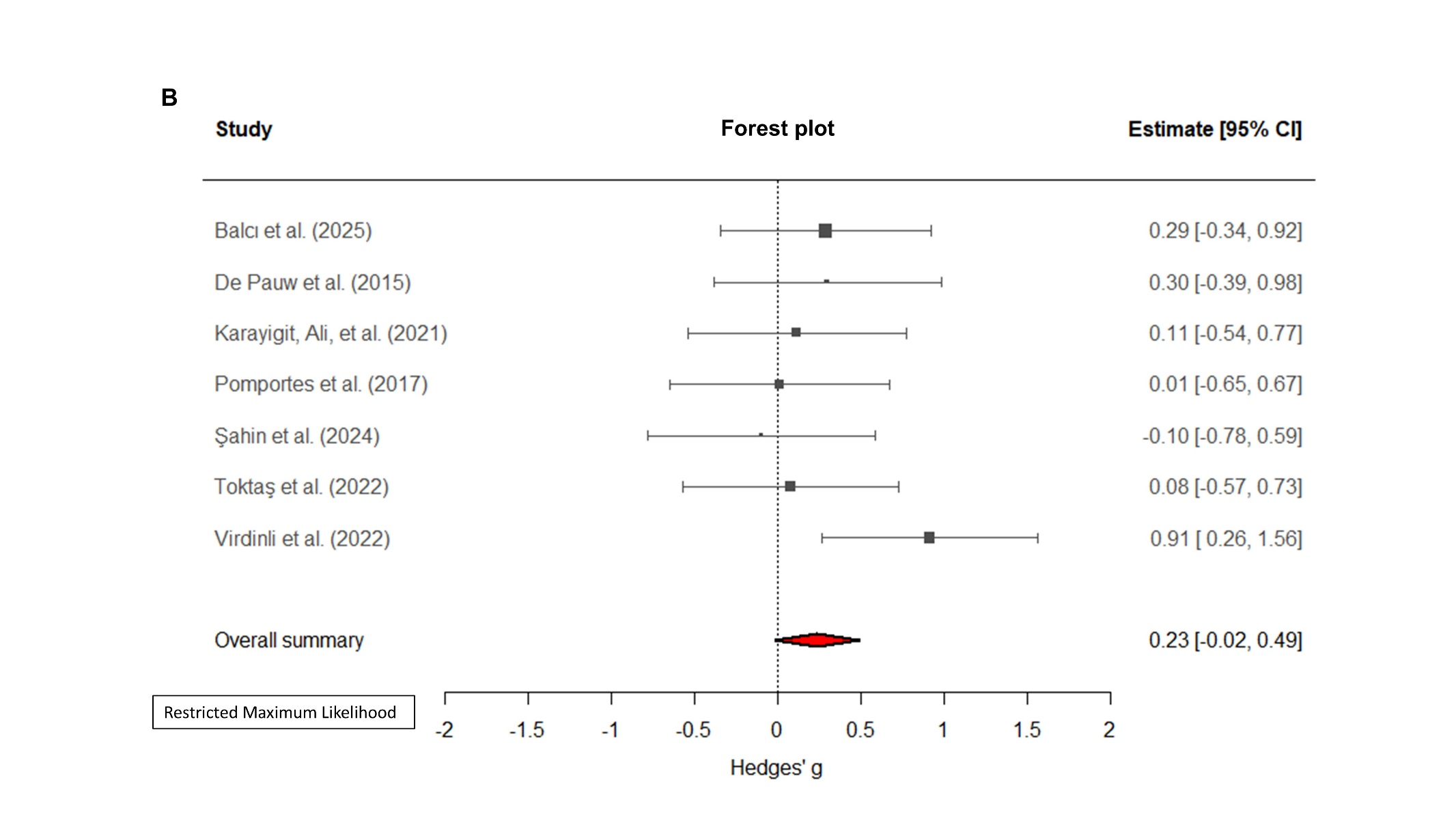


***Notes: Hedge's g***, the effect size indicators used in the pooled; ***95%CI***, 95% confidence interval; ***The size of each black square***, represents the relative weight of the study in the meta-analysis; ***A***, for exercise performance; ***B***, for cognitive performance.

**Electronic Supplementary Material Appendix S4 (Simple linear regression analysis of caffeine mouth rinse total exposure dose)**

**
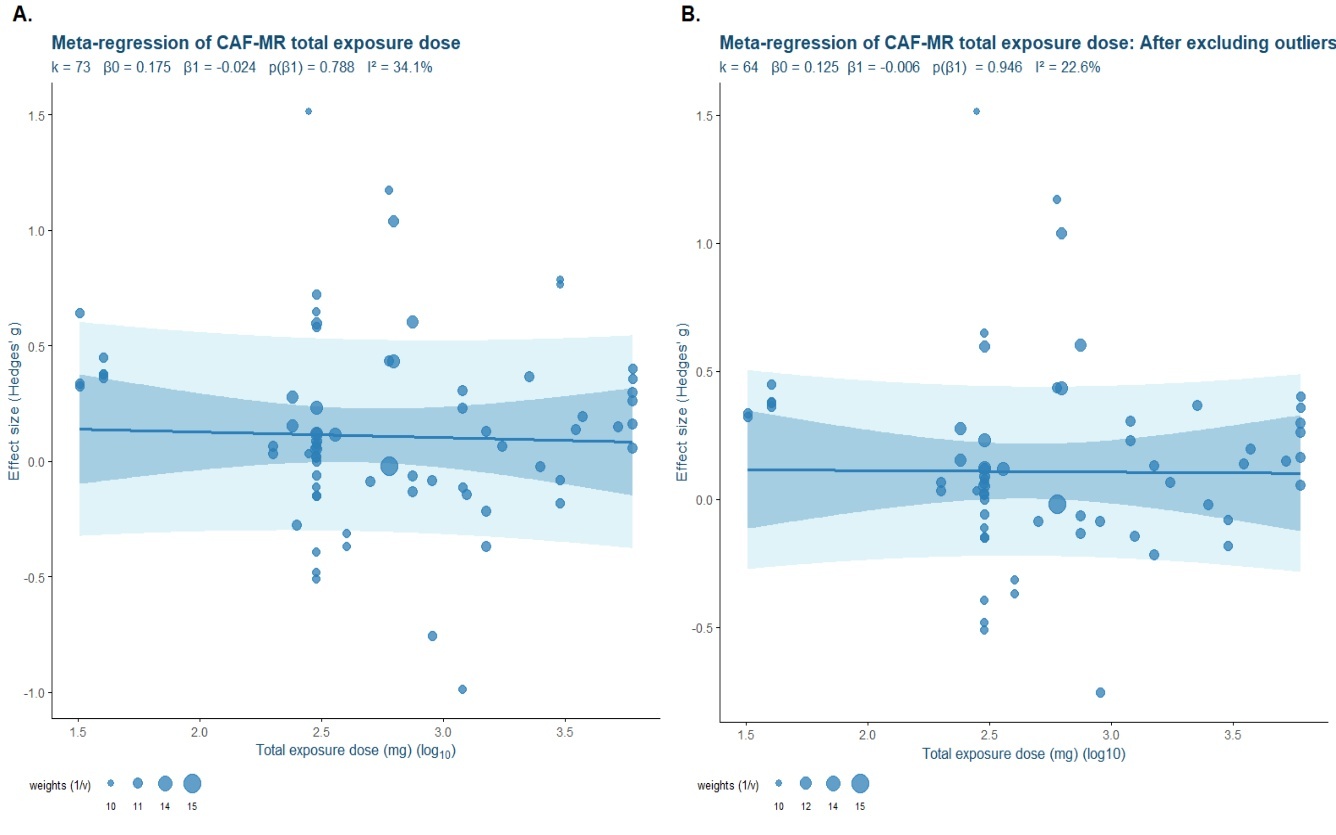
**

**Notes: A**, represents the result of original data; **B**, represents the results after outliers excluding; ***k***, represents effect size numbers; ***β0,*** represents the intercept; ***β1*** represent the slopes; ***I^2^,*** means heterogeneity; ***The blue circles***, represents the effect sizes; ***The blue shaded part,*** represents the 95% confidence interval; ***The outermost blue dotted line***, represents the prediction interval.

**Electronic Supplementary Material Appendix S5 (Quadratic meta regression analysis of caffeine mouth rinse total exposure dose)**

**
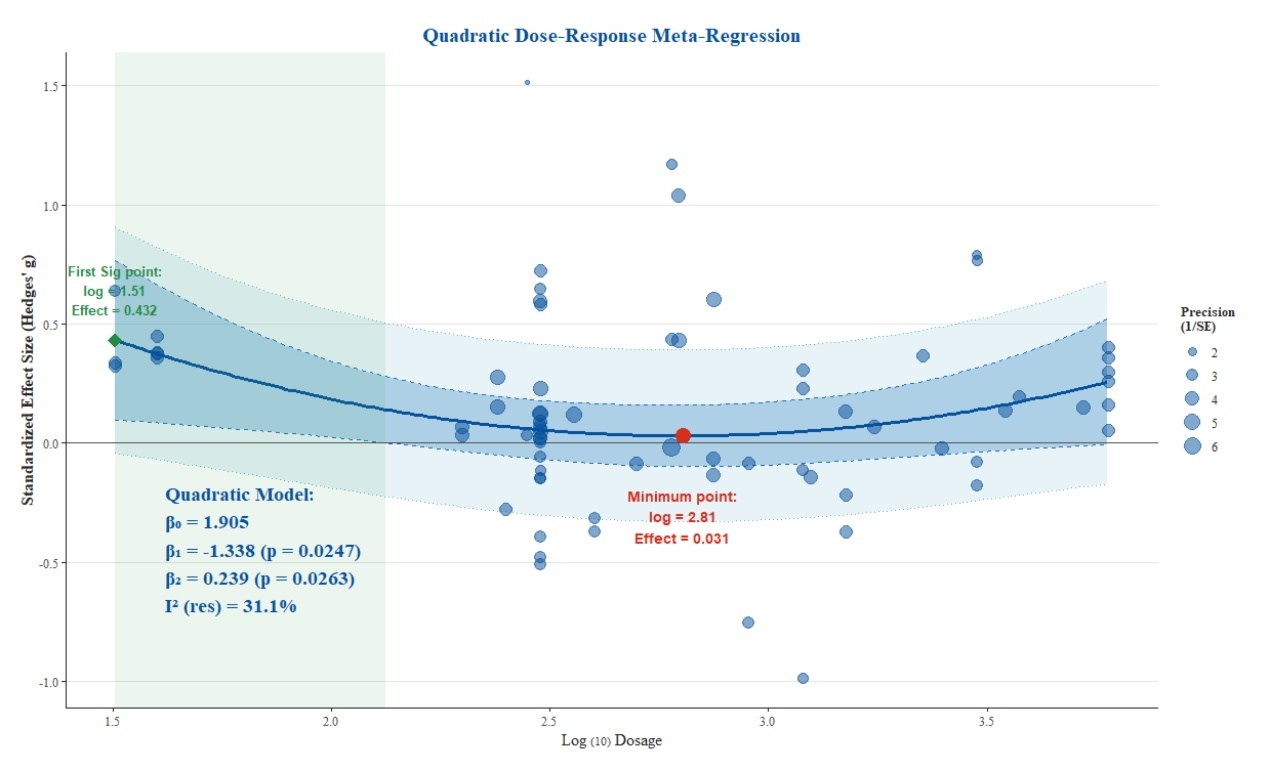
**

**Notes: *k***, represents effect size numbers; ***β0,*** represents the intercept; ***β1*** and ***β2,*** represent the slopes; ***I^2^,*** means heterogeneity; ***The blue shaded part,*** represents the 95% confidence interval; ***The outermost blue dotted line***, represents the prediction interval; ***The green area***, represents the significant dose effect interval (p < 0.05; log_(10)_: 1.505 – 2.125; total exposure dosage (mg): 32 – 133.40).

**Electronic Supplementary Material Appendix S6 (Total Exposure Dose Meta-Regression Analysis: Original Data and After Excluding Outliers)**

| **Original** | | | | | | |  | **After excluding outliers** | | | | | | |
| --- | --- | --- | --- | --- | --- | --- | --- | --- | --- | --- | --- | --- | --- | --- |
| Model;  AIC | β0 | β1 | β2 | β3 | I^2^ | k |  | Model;  AIC | β0 | β1 | β2 | β3 | I^2^ | k |
| Linear  58.55 | g = 0.18  CI [-0.32, 0.67]  p = 0.48 | g = -0.02  CI [-0.20, 0.16]  p = 0.79 | N/A | N/A | 34% | 73 |  | Linear  37.90 | g = 0.12  CI [-0.36, 0.61]  p = 0.61 | g = -0.01  CI [-0.18, 0.17]  p = 0.95 | N/A | N/A | 23% | 64 |
| Quadratic  (TRUE)  53.66 | g = 1.91  CI [0.32, 3.49]  p = 0.02 | g = -1.34  CI [-2.51, -0.17]  p = 0.02 | g = 0.24  CI [0.03, 0.45]  p = 0.03 | N/A | 31% | 73 |  | Quadratic  (TRUE)  28.83 | g = 1.83  CI [-0.05, 3.71]  p = 0.056 | g = -1.26  CI [-2.61, 0.09]  p = 0.07 | g = 0.22  CI [-0.02, 0.46]  p = 0.07 | N/A | 10% | 61 |
| Quadratic  (FALSE)  53.66 | g = 0.12  CI [0.01, 0.22]  p = 0.04 | g = -0.20  CI [-1.04, 0.64]  p = 0.64 | g = 0.94  CI [0.11, 1.77]  p = 0.03 | N/A | 31% | 73 |  | Quadratic  (FALSE)  28.83 | g = 0.10  CI [0.01, 0.20]  p = 0.04 | g = -0.09  CI [-0.85, 0.66]  p = 0.81 | g = 0.70  CI [-0.05, 1.45]  p = 0.07 | N/A | 10% | 61 |
| Cubic  (TRUE)  55.71 | g = 1.40  CI [-4.68, 7.47]  p = 0.65 | g = -0.70  CI [-8.16, 6.77]  p = 0.86 | g = -0.02  CI [-2.96, 2.93]  p = 0.99 | g = 0.03  CI [-0.34, 0.41]  p = 0.86 | 32% | 73 |  | Cubic  (TRUE)  30.51 | g = 2.41  CI [-4.99, 9.80]  p = 0.52 | g = -1.92  CI [-10.83, 6.70]  p = 0.67 | g = 0.45  CI [-3.01, 3.91]  p = 0.80 | g = -0.02  CI [-0.46, 0.41]  p = 0.91 | 6% | 61 |
| Cubic  (FALSE)  55.71 | g = 0.11  CI [0.01, 0.22]  p = 0.04 | g = -0.19  CI [-1.05, 0.67]  p = 0.66 | g = 0.95  CI [0.10, 1.79]  p = 0.03 | g = 0.07  CI [-0.69, 0.82]  p = 0.86 | 32% | 73 |  | Cubic  (FALSE)  30.51 | g = 0.11  CI [0.01, 0.21]  p = 0.02 | g = 0.08  CI [-0.68, 0.85]  p = 0.83 | g = 0.81  CI [0.05, 1.56]  p = 0.04 | g = -0.04  CI [-0.80, 0.71]  p = 0.91 | 6% | 61 |

***Notes: AIC,*** represents akaike information criterion using for model selection; β0 represents the intercept; ***β1***, ***β2***, and ***β3*** represent the slopes; ***TRUE*** is equal to raw = TRUE in the model, which means that the original polynomial is used to fit the nonlinear relationship; ***FALSE*** is equal to raw = FALSE in the model, which means that the orthogonal polynomial is used to fit the nonlinear relationship; ***CI*** means 95% confidence interval; ***I^2^*** means heterogeneity; ***k*** represents the number of effects remaining after removing outliers; ***N/A*** means not applicable.

**Electronic Supplementary Material Appendix S7 (Exploratory Analysis of Rinse Frequency/Times: Original Data and After Excluding Outliers)**

| **Table 1: Rinse Frequency/Times (Exploratory Analysis as Categorical Variables)** | | | | | | | | | | | | | | | | |
| --- | --- | --- | --- | --- | --- | --- | --- | --- | --- | --- | --- | --- | --- | --- | --- | --- |
| **Original** | | | | | | | |  | **After excluding outliers** | | | | | | | |
| Subgroup | Hedges’g | 95% CI | P-Value | PI | Power | I^2^ | k |  | Subgroup | Hedges’g | 95% CI | P-Value | PI | Power | I^2^ | k |
| Single  (1 time) | 0.06 | [-0.06, 0.17] | 0.35 | [-0.23, 0.34] | 5% | 0 | 45 |  | Single  (1 time) | 0.01 | [0.10, 0.12] | 0.87 | [0.22, 0.24] | 5% | 0 | 42 |
| Moderate  (2-9 times) | 0.17 | [0.06, 0.28] | **< 0.01** | [-0.11, 0.45] | 11% | 36% | 51 |  | Moderate  (2-9 times) | 0.25 | [0.13, 0.36] | **< 0.01** | [0.01, 0.48] | 14% | 0 | 39 |
| High  (≥10 times) | -0.01 | [-0.30, 0.29] | 0.99 | [-0.39, 0.39] | 5% | 0 | 16 |  | High  (≥10 times) | 0.03 | [-0.22, 0.28] | 0.81 | [-0.29, 0.35] | 5% | 0 | 15 |

***Notes***: ***K***, the total number of effects included in the pooled effect size; ***Hedges'g***, the effect size indicators used in the pooled; ***95% CI***, 95% confidence interval; ***P-value***, statistically significant P values for pooled results; ***I^2^***, quantitative indicators of heterogeneity; ***Power***, statistical power for pooled effect size.

| **Table 2: Rinse Frequency/Times (Exploratory Analysis as Continuous Variables)** | | | | | | | | | | | | | | |
| --- | --- | --- | --- | --- | --- | --- | --- | --- | --- | --- | --- | --- | --- | --- |
| **Original** | | | | | | |  | **After excluding outliers** | | | | | | |
| Model;  AIC | β0 | β1 | β2 | β3 | I^2^ | k |  | Model;  AIC | β0 | β1 | β2 | β3 | I^2^ | k |
| Linear  46.68 | g = 0.10  CI [-0.02, 0.22]  p = 0.09 | g < 0.01  CI [-0.02, 0.02]  p = 0.10 | N/A | N/A | 20% | 112 |  | Linear  -6.77 | g = 0.13  CI [0.05, 0.20]  p = 0.01 | g < 0.01  CI [-0.01, 0.01]  p = 0.97 | N/A | N/A | 0 | 96 |
| Quadratic  (TRUE)  45.21 | g = 0.04  CI [-0.04, 0.13]  p = 0.33 | g = 0.04  CI [0.01, 0.07]  p = 0.01 | g = -0.01  CI [-0.01, 0.01]  p = 0.01 | N/A | 16% | 112 |  | Quadratic  (TRUE)  20.16 | g = 0.05  CI [-0.08, 0.18]  p = 0.49 | g = 0.03  CI [-0.07, 0.13]  p = 0.54 | g < 0.01  CI [-0.01, 0.13]  p = 0.88 | N/A | 0 | 87 |
| Quadratic  (FALSE)  45.21 | g = 0.12  CI [0.07, 0.17]  p < 0.01 | g = -0.32  CI [-0.84, 0.21]  p = 0.24 | g = -0.76  CI [-1.28, -0.24]  p < 0.01 | N/A | 16% | 112 |  | Quadratic  (FALSE)  20.16 | g = 0.13  CI [0.07, 0.18]  p < 0.01 | g = 0.54  CI [0.01, 1.08]  p = 0.048 | g = -0.04  CI [-0.59, 0.51]  p = 0.88 | N/A | 0 | 87 |
| Cubic  (TRUE)  47.86 | g = 0.06  CI [-0.09, 0.20]  p = 0.45 | g = 0.03  CI [-0.10, 0.15]  p = 0.66 | g < 0.01  CI [-0.02, 0.02]  p = 0.97 | g < -0.01  CI [-0.01, 0.01]  p = 0.83 | 17% | 112 |  | Cubic  (TRUE)  14.70 | g = 0.01  CI [-0.17, 0.18]  p = 0.94 | g = 0.09  CI [-0.08, 0.27]  p = 0.30 | g = -0.01  CI [-0.05, 0.02]  p = 0.46 | g < 0.01  CI [-0.01, 0.01]  p = 0.52 | 0 | 92 |
| Cubic  (FALSE)  47.86 | g = 0.12  CI [0.08, 0.17]  p < 0.01 | g = -0.31  CI [-0.84, 0.21]  p = 0.24 | g = -0.77  CI [-1.29, 0.24]  p = 0.01 | g = -0.06  CI [-0.61, 0.49]  p = 0.83 | 17% | 112 |  | Cubic  (FALSE)  14.70 | g = 0.12  CI [0.06, 0.17]  p < 0.01 | g = -0.25  CI [-0.80, 0.28]  p = 0.35 | g = -0.51  CI [-1.07, 0.05]  p = 0.07 | g = 0.20  CI [-0.40, 0.80]  p = 0.52 | 0 | 92 |

***Notes: AIC,*** represents akaike information criterion using for model selection; β0 represents the intercept; ***β1***, ***β2***, and ***β3*** represent the slopes; ***TRUE*** is equal to raw = TRUE in the model, which means that the original polynomial is used to fit the nonlinear relationship; ***FALSE*** is equal to raw = FALSE in the model, which means that the orthogonal polynomial is used to fit the nonlinear relationship; ***CI*** means 95% confidence interval; ***I^2^*** means heterogeneity; ***k*** represents the number of effects remaining after removing outliers; ***N/A*** means not applicable.

**Electronic Supplementary Material Appendix S8 (Exploratory linear regression analysis of total exposure dose and rinsing time)**


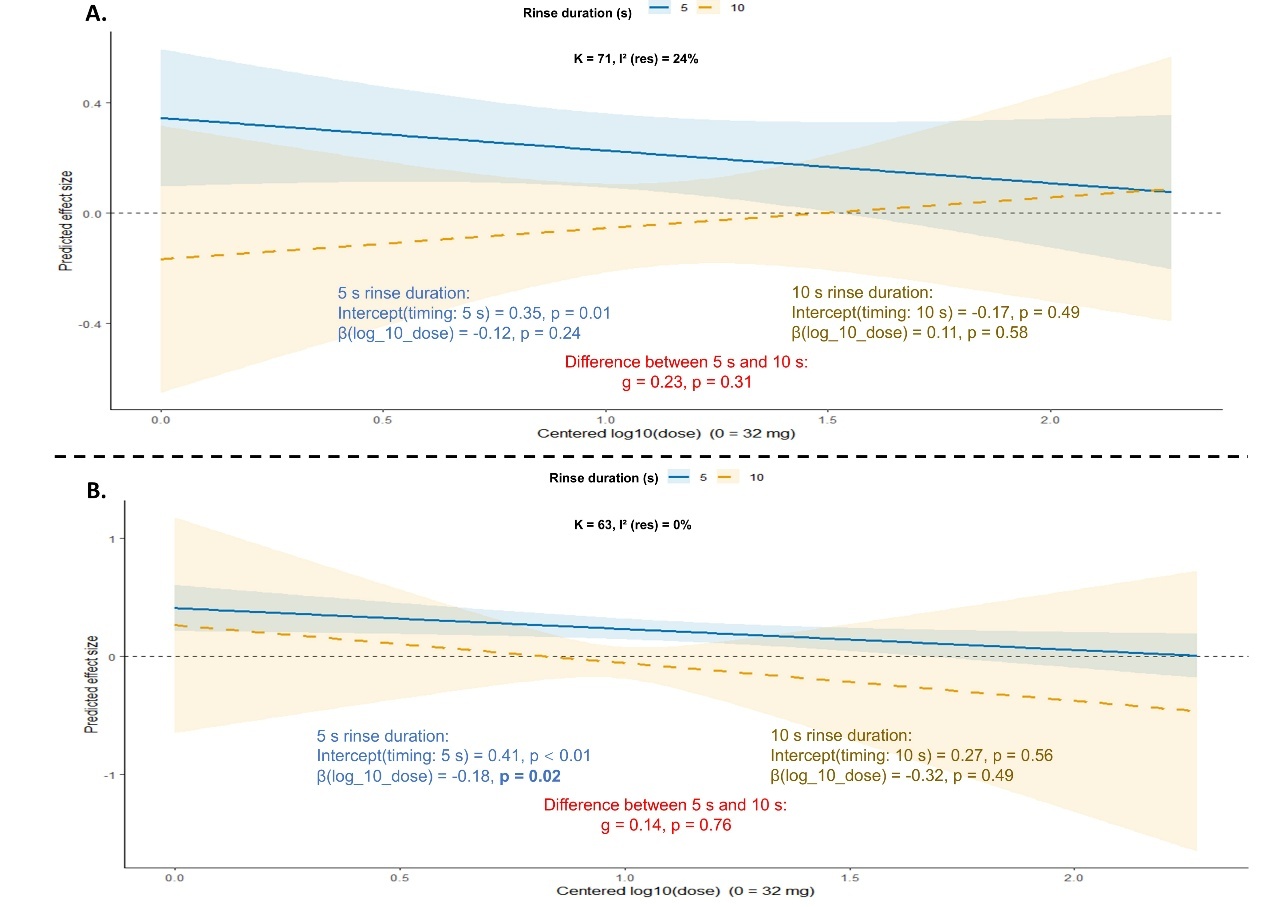


**Notes: A**, represents the result of original data; **B**, represents the results after outliers excluding; ***k***, represents effect size numbers; ***β1***, represent the slopes; ***Hedge's g***, the effect size indicators used in the pooled; ***I^2^,*** means heterogeneity; ***P-value***, statistically significant P values ​​for pooled results; ***The shaded part,*** represents the 95% confidence interval.

**Electronic Supplementary Material Appendix S9 (Moderator analysis for cognitive primary results)**


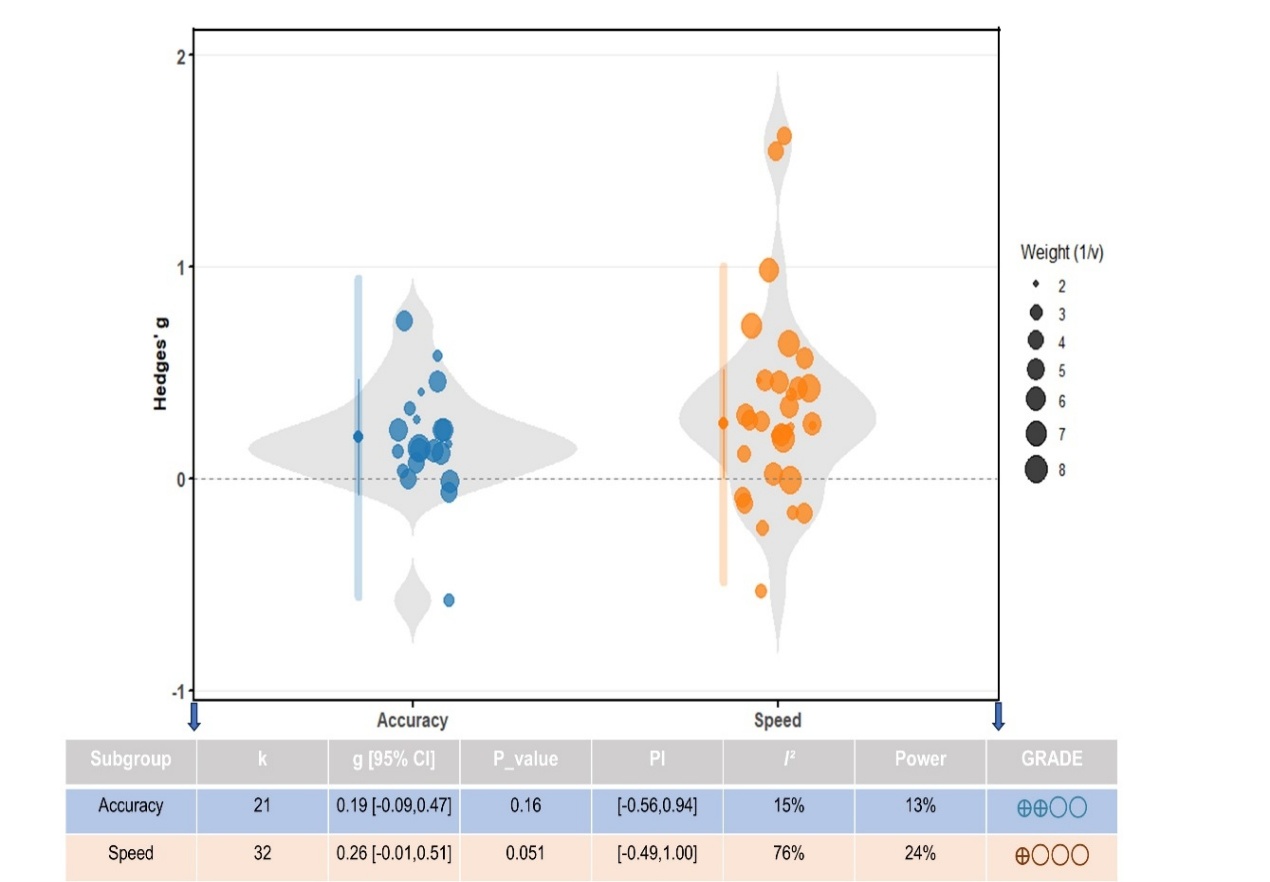


**Notes: Accuracy, represents accuracy-based cognitive performance; Speed, represents speed-based cognitive performance; K, the total number of effects included in the pooled effect size; Hedge's g, the effect size indicators used in the pooled; 95%CI, 95% confidence interval; 95%PI, prediction Interval; P-value, statistically significant P values for pooled results; I2, quantitative indicators of heterogeneity; Power, statistical power for pooled effect size; GRADE, grading of recommendations assessment, development, and evaluation, a system for evaluating the quality of evidence and strength of recommendations.**

**Electronic Supplementary Material Appendix S10 (ROB2 Assessment Tool for Risk of Bias: Exercise and Cognition)**


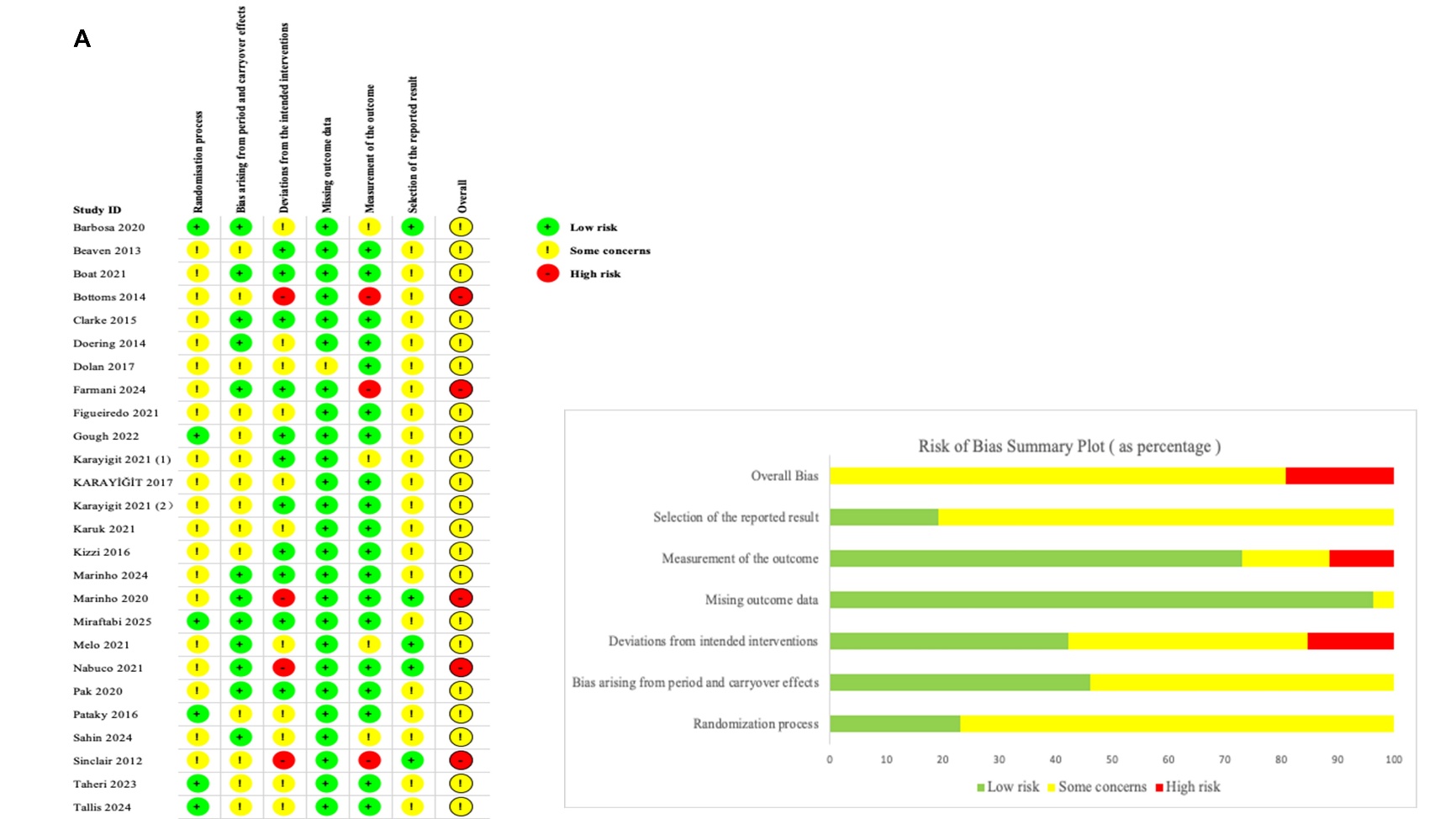


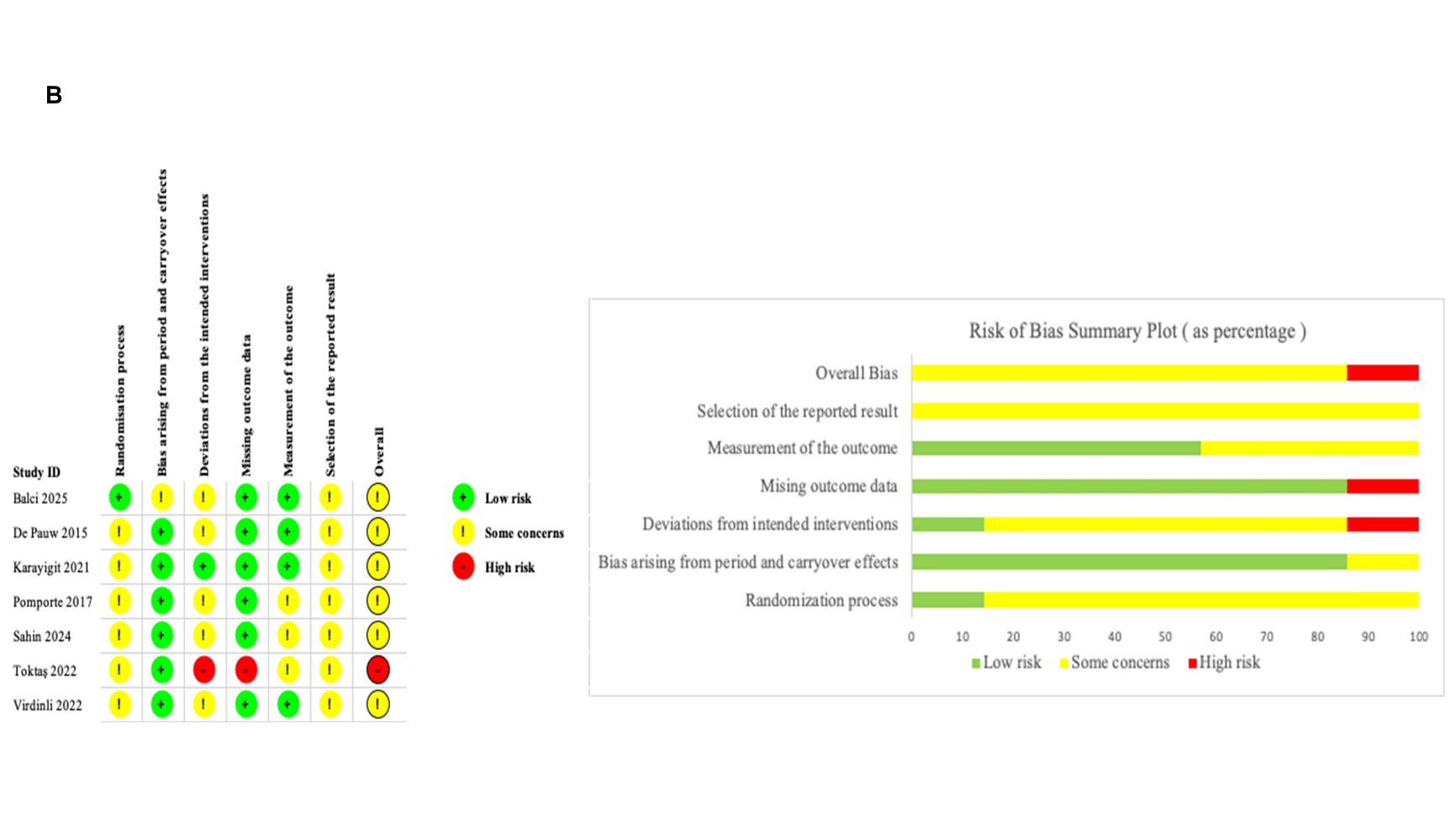


***Notes:*** ***A***, for exercise performance; ***B***, for cognitive performance.

**Electronic Supplementary Material Appendix S11 (Funnel Plot: Exercise and Cognition)**


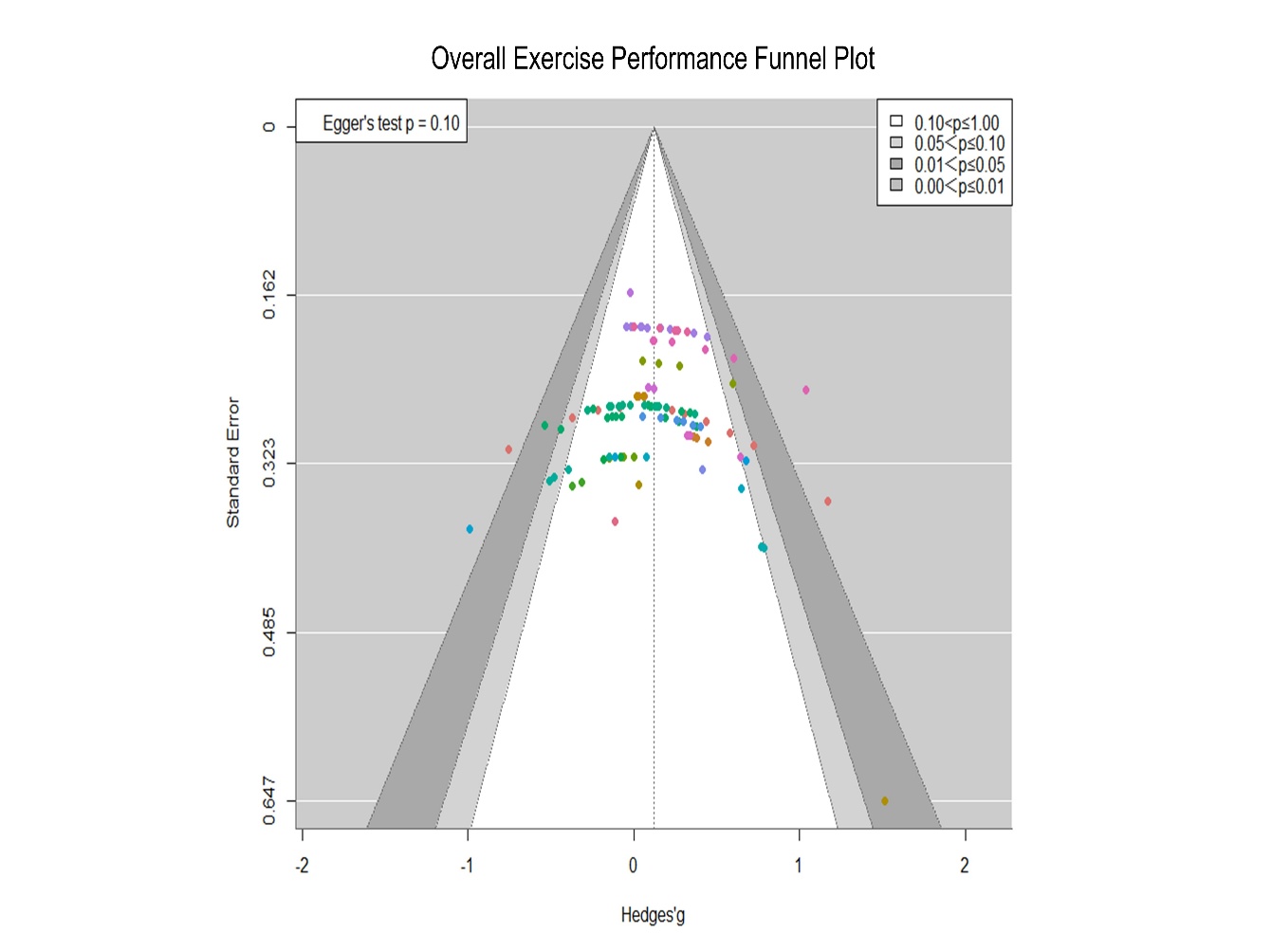
**Exercise:**


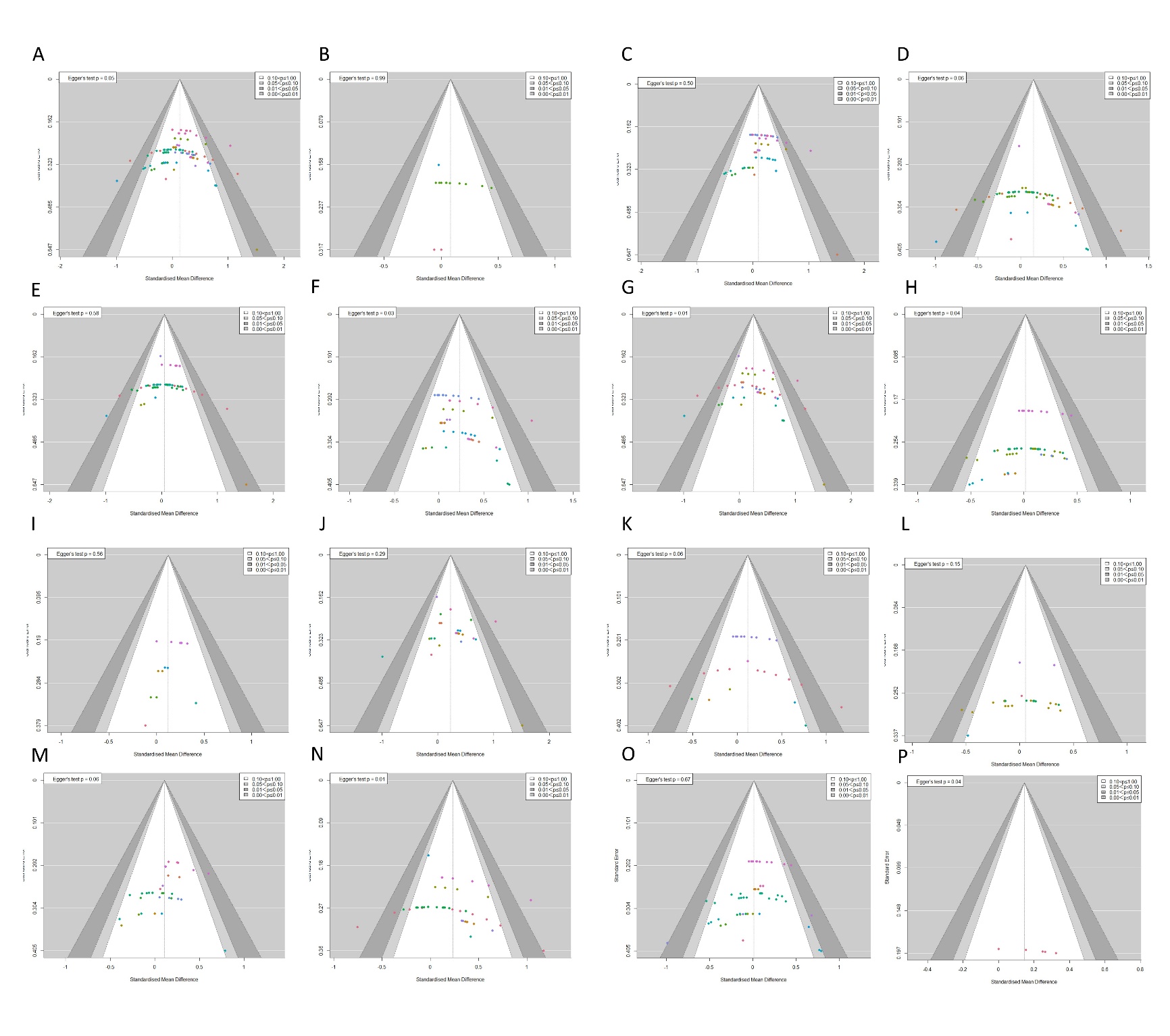


***Notes: Colorful circles***, represent different effect size points. From ***A*** to ***O***, they are male, mixed, trained, untrained, low caffeine intake level, unclear caffeine intake level, fed, fast, unspecified, aerobic endurance, anaerobic performance, muscular endurance, strength/power, 5 s, 10 s, 30 s.


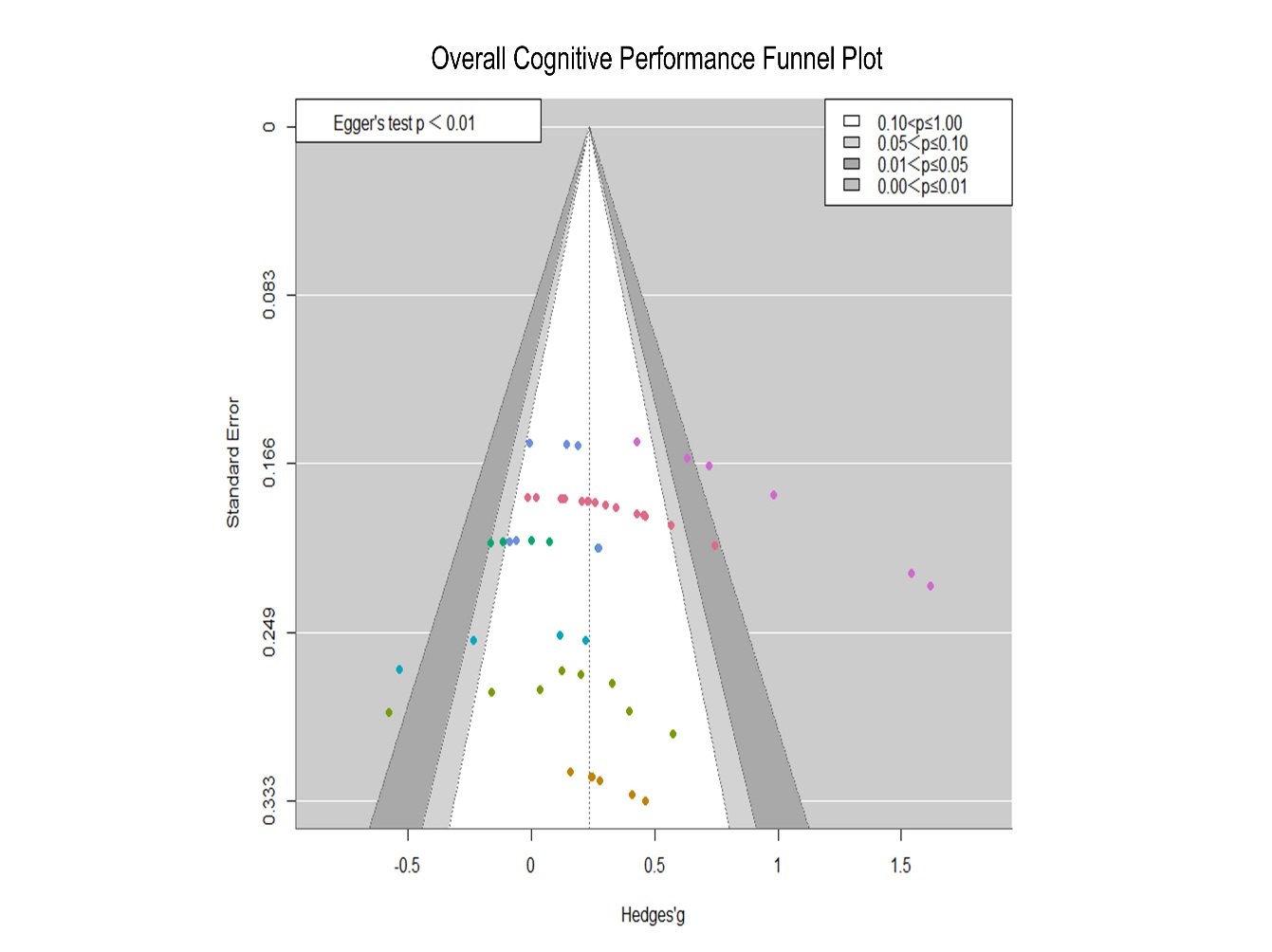
**Cognition:**


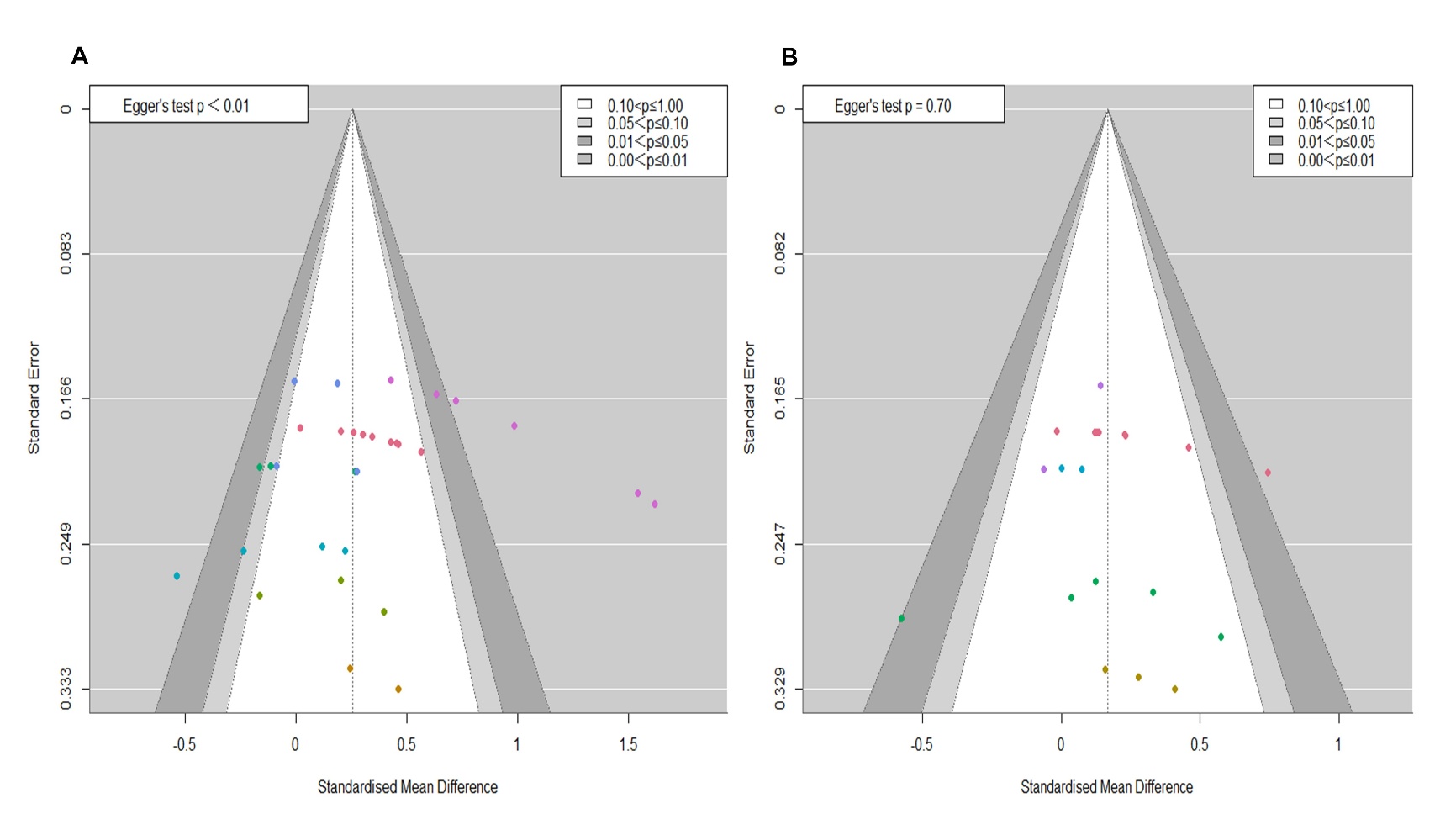


***Notes: Colorful circles***, represent different effect size points. ***A***, represents speed-based performance, ***B***, represents accuracy-based performance.

**Electronic Supplementary Material Appendix S12 (Power Visualization: Exercise and Cognition)**

**
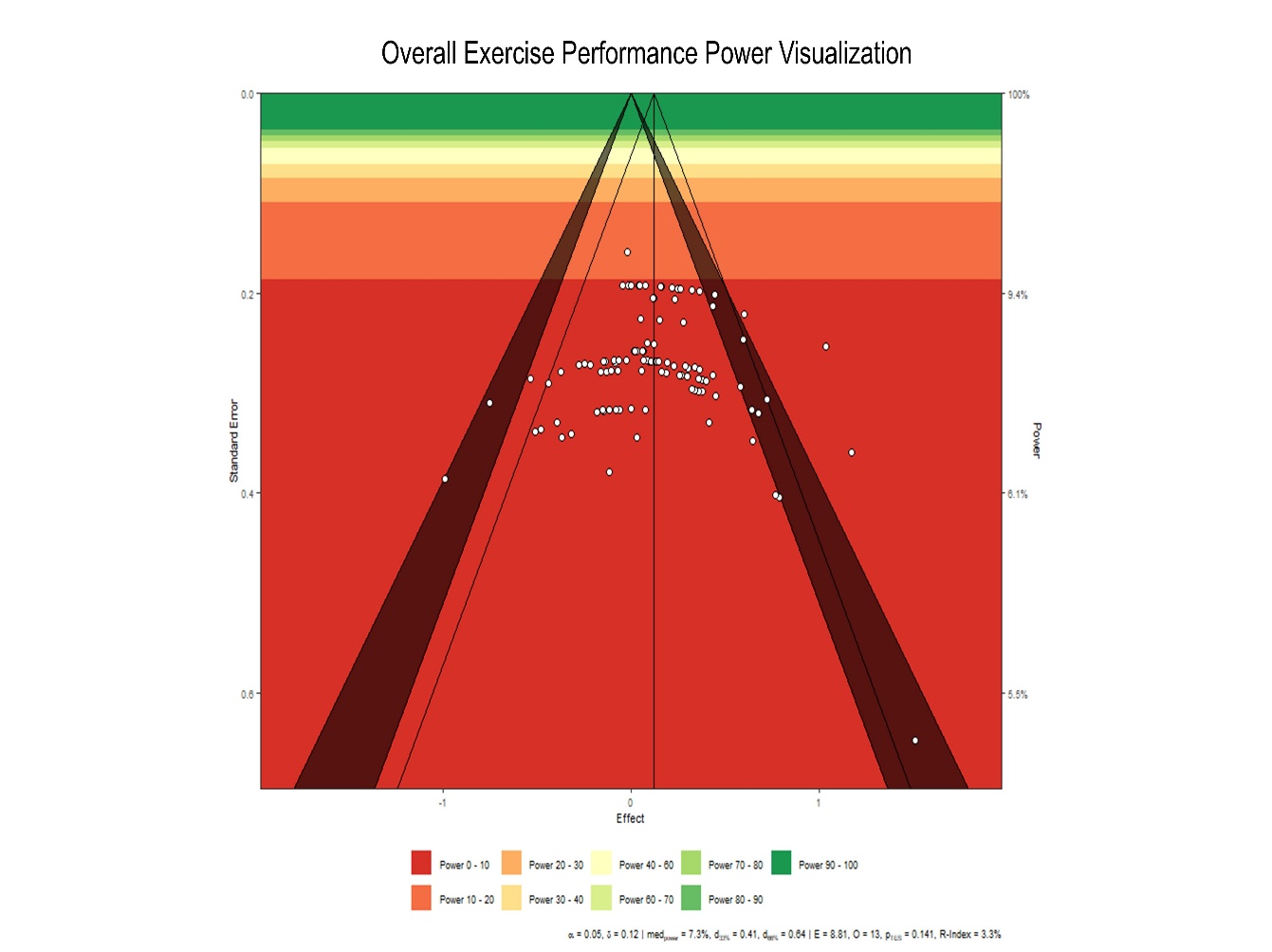
Exercise:**


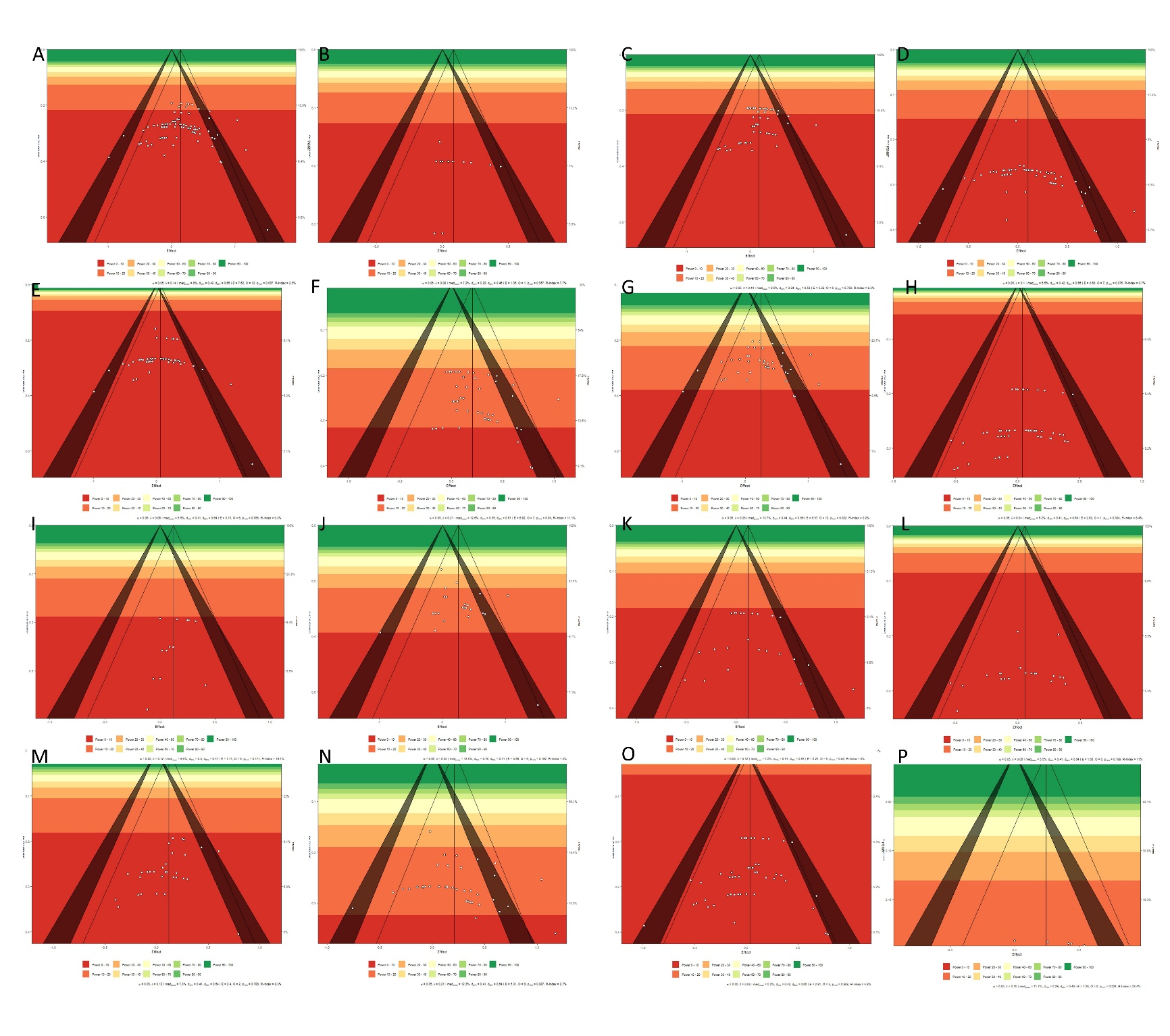


***Notes*:** The vertical solid line represents the pooled effect size, and the vertical dash line represents the adjusted pooled effect size. Significance contours at .05 and .01 levels are noted by the shaded area. ***manpower*** indicates the median power of all included effect sizes. ***d33%*** and ***d66%*** indicate the true effect sizes necessary for achieving 33% and 66% levels of median power. ***E, O, and PTES*** show the results of a test of excess significance. ***R-index*** denotes the expected replicability of findings. From ***A*** to ***O***, they are male, mixed, trained, untrained, low caffeine intake level, unclear caffeine intake level, fed, fast, unspecified, aerobic endurance, anaerobic performance, muscular endurance, strength/power, 5 s, 10 s, 30 s.

**
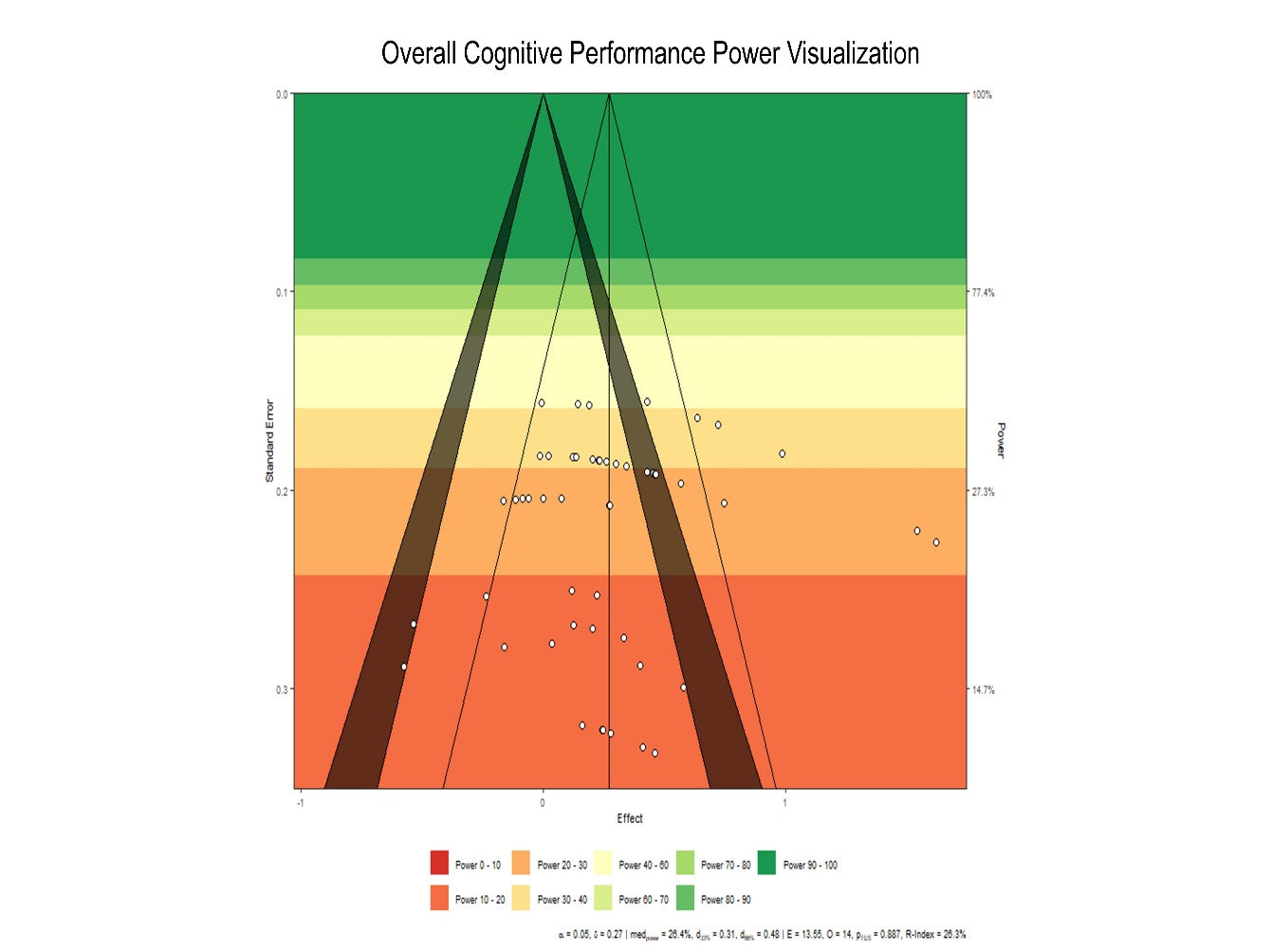
Cognition:**


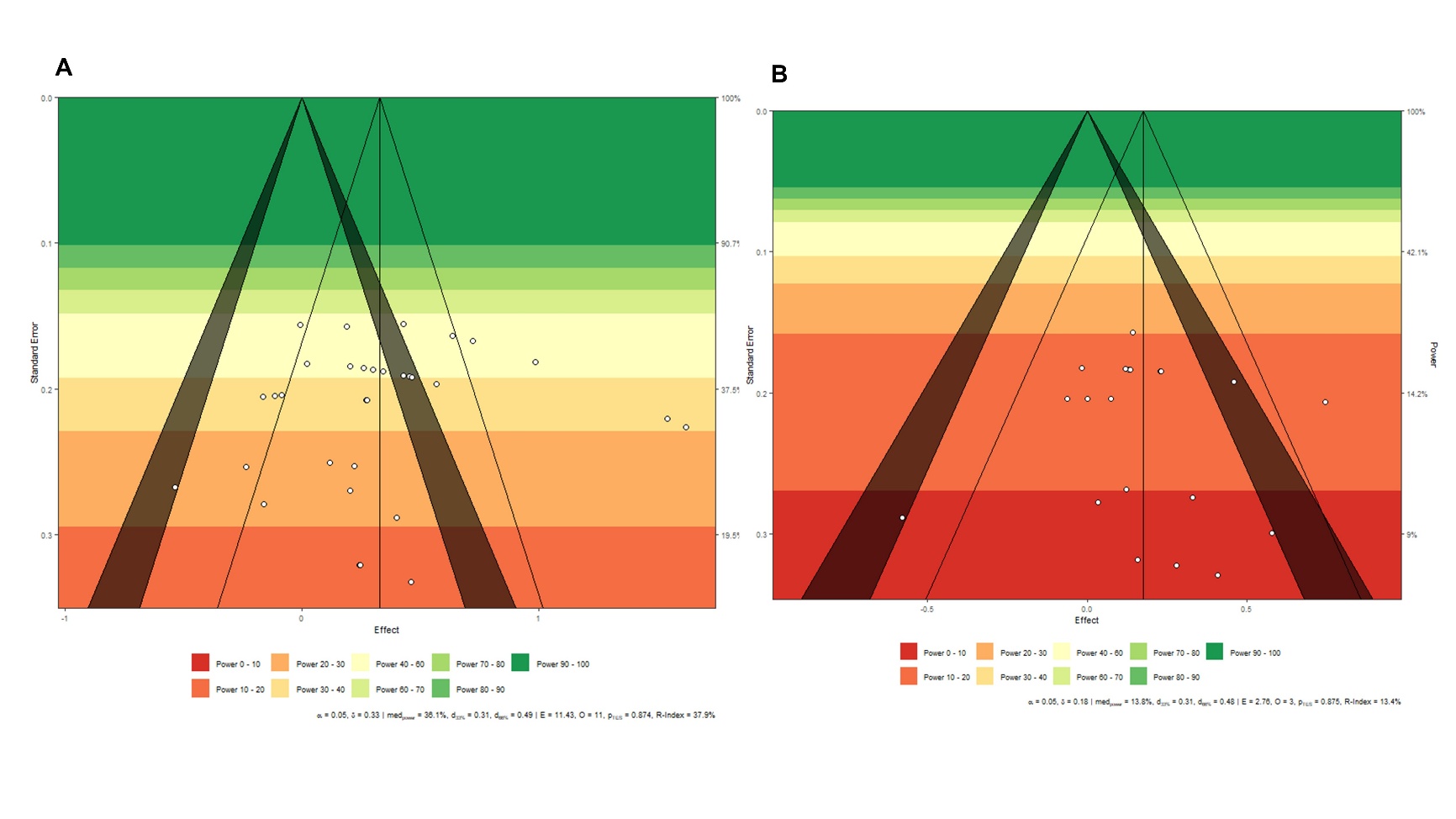


***Notes*:** The vertical solid line represents the pooled effect size, and the vertical dash line represents the adjusted pooled effect size. Significance contours at .05 and .01 levels are noted by the shaded area. ***manpower*** indicates the median power of all included effect sizes. ***d33%*** and ***d66%*** indicate the true effect sizes necessary for achieving 33% and 66% levels of median power. ***E, O, and PTES*** show the results of a test of excess significance. ***R-index*** denotes the expected replicability of findings. ***A***, represents speed-based performance, ***B***, represents accuracy-based performance.

**Electronic Supplementary Material Appendix S13 (PEDro Assessment: Exercise and Cognition)**

| Table A. PEDro assessment results of exercise performance researches | | | | | | | | | | | | | |
| --- | --- | --- | --- | --- | --- | --- | --- | --- | --- | --- | --- | --- | --- |
| Study | Item 1 | Item 2 | Item 3 | Item 4 | Item 5 | Item 6 | Item 7 | Item 8 | Item 9 | Item 10 | Item 11 | Item 12 | Total |
|  |  |  |  |  |  |  |  |  |  |  |  |  |  |
| Barbosa et al. (2020) | Yes | Yes | No | Yes | No | No | No | No | Yes | Yes | Yes | Yes | 6 |
| Beaven et al. (2013) | Yes | No | No | Yes | Yes | Yes | Yes | No | Yes | Yes | Yes | Yes | 8 |
| Boat et al. (2021) | Yes | No | No | Yes | Yes | Yes | No | Yes | Yes | Yes | Yes | No | 7 |
| Bottoms et al. (2014) | Yes | No | No | Yes | No | No | No | Yes | Yes | Yes | Yes | No | 5 |
| Clarke et al. (2015) | Yes | Yes | No | Yes | Yes | No | No | Yes | Yes | Yes | Yes | No | 7 |
| Doering et al. (2014) | Yes | Yes | No | Yes | Yes | No | Yes | Yes | Yes | Yes | Yes | No | 8 |
| Dolan et al. (2017) | Yes | Yes | Yes | Yes | No | Yes | Yes | No | No | Yes | Yes | No | 7 |
| Farmani et al. (2024) | Yes | Yes | Yes | Yes | No | No | No | Yes | Yes | Yes | Yes | No | 7 |
| Figueiredo et al. (2021) | Yes | Yes | No | Yes | No | Yes | Yes | Yes | Yes | Yes | Yes | No | 8 |
| Gough et al. (2022) | Yes | Yes | No | Yes | Yes | Yes | Yes | Yes | Yes | Yes | Yes | No | 9 |
| Karayiğit et al. (2017) | Yes | Yes | No | Yes | Yes | No | No | Yes | Yes | Yes | Yes | No | 8 |
| Karayigit, Ali et al. (2021) | Yes | Yes | No | Yes | Yes | No | Yes | Yes | Yes | Yes | Yes | No | 8 |
| Karayigit, Koz et al. (2021) | Yes | No | Yes | Yes | No | Yes | Yes | Yes | Yes | Yes | Yes | Yes | 9 |
| Karuk et al. (2022) | Yes | Yes | Yes | Yes | Yes | No | Yes | No | No | Yes | Yes | No | 7 |
| Kizzi et al. (2016) | Yes | Yes | Yes | No | Yes | No | No | Yes | Yes | Yes | Yes | No | 7 |
| Marinho et al. (2020) | Yes | Yes | No | Yes | Yes | No | No | Yes | Yes | Yes | Yes | No | 7 |
| Marinho et al. (2024) | Yes | Yes | No | Yes | Yes | No | No | Yes | Yes | Yes | Yes | Yes | 8 |
| Melo et al. (2021) | Yes | Yes | No | Yes | No | No | No | Yes | Yes | Yes | Yes | No | 6 |
| Miraftabi et al. (2025) | Yes | Yes | No | Yes | Yes | No | No | Yes | Yes | Yes | Yes | No | 7 |
| Nabuco et al. (2021) | Yes | Yes | Yes | Yes | Yes | No | No | Yes | Yes | Yes | Yes | No | 8 |
| Pak et al. (2020) | Yes | Yes | No | Yes | Yes | No | No | Yes | Yes | Yes | Yes | No | 7 |
| Pataky et al. (2016) | Yes | Yes | No | Yes | Yes | Yes | Yes | Yes | No | Yes | Yes | No | 8 |
| Şahin et al. (2024) | Yes | No | No | Yes | No | No | No | Yes | Yes | Yes | Yes | No | 5 |
| Sinclair & Bottoms (2015) | Yes | Yes | No | Yes | Yes | No | No | Yes | Yes | Yes | Yes | No | 7 |
| Taheri Karami et al. (2023) | Yes | Yes | No | Yes | Yes | No | Yes | Yes | Yes | Yes | Yes | No | 8 |
| Tallis et al. (2024) | Yes | Yes | No | Yes | Yes | Yes | Yes | Yes | No | Yes | Yes | No | 8 |

| Table B. PEDro assessment results of cognitive performance researches | | | | | | | | | | | | | |
| --- | --- | --- | --- | --- | --- | --- | --- | --- | --- | --- | --- | --- | --- |
| Study | Item 1 | Item 2 | Item 3 | Item 4 | Item 5 | Item 6 | Item 7 | Item 8 | Item 9 | Item 10 | Item 11 | Item 12 | Total |
|  |  |  |  |  |  |  |  |  |  |  |  |  |  |
| Balcı et al. (2025) | Yes | Yes | Yes | Yes | Yes | No | Yes | Yes | No | Yes | Yes | No | 8 |
| De Pauw et al. (2015) | Yes | Yes | No | Yes | No | No | No | Yes | Yes | Yes | No | No | 5 |
| Karayigit, Ali, et al. (2021) | Yes | Yes | Yes | Yes | Yes | No | No | Yes | Yes | Yes | Yes | No | 8 |
| Pomportes et al. (2017) | Yes | No | No | Yes | No | No | No | Yes | Yes | Yes | Yes | Yes | 6 |
| Şahin et al. (2024) | Yes | No | No | Yes | No | No | No | Yes | Yes | Yes | Yes | No | 5 |
| Toktaş et al. (2022) | Yes | Yes | No | Yes | No | No | No | Yes | No | Yes | Yes | Yes | 6 |
| Virdinli et al. (2022) | Yes | Yes | Yes | Yes | No | Yes | Yes | Yes | No | Yes | Yes | No | 8 |

**Electronic Supplementary Material Appendix S14 (GRADE Assessment: Exercise and Cognition)**

| **Outcome** | **K** | **Certainty of Evidence Assessment** | | | | | **Hedge’s g [95% CI] *** | **GRADE†** |
| --- | --- | --- | --- | --- | --- | --- | --- | --- |
|  |  | **Risk of Bias** | **Inconsistency** | **Indirectness** | **Imprecision** | **Others** |  |  |
| Primary Outcome | | | | | | | | |
| Exercise performance | K = 114 | Serious | Not serious | Not serious | Not serious | None | 0.12 [0.04, 0.21] ***** | ⨁⨁⨁◯ Moderate |
| Cognitive  performance | K = 53 | Serious | Serious | Not serious | Not serious | Publication bias | 0.23 [-0.02, 0.49] | ⨁◯◯◯ Very low |
| Sex | | | | | | | | |
| Male | K = 91 | Serious | Not serious | Not serious | Not serious | None | 0.14 [0.04,0.23] ***** | ⨁⨁⨁◯ Moderate |
| Female | K = 8 | Not serious | Not serious | Not serious | Extremely serious | None | 0.11 [-0.15,0.37] | ⨁◯◯◯ Very low |
| Mixed | K = 15 | Not serious | Not serious | Serious | Serious | None | 0.04 [-0.20,0.28] | ⨁⨁◯◯ Low |
| Training Level | | | | | | | | |
| Trained | K = 57 | Serious | Serious | Not serious | Not serious | None | 0.10 [-0.02,0.23] | ⨁⨁◯◯ Low |
| Untrained | K = 57 | Not serious | Not serious | Not serious | Not serious | None | 0.15 [0.02,0.28] ***** | ⨁⨁⨁⨁ High |
| Habitual caffeine intake | | | | | | | | |
| Low (0–150 mg/day) | K = 55 | Not serious | Not serious | Very serious | Not serious | None | 0.04 [-0.07,0.15] | ⨁⨁◯◯ Low |
| Moderate (150–300 mg/day) | K = 8 | Not serious | Serious | Serious | Very serious | None | -0.14 [-0.39,0.12] | ⨁◯◯◯ Very low |
| High (>300 mg/day) | K = 1 | Not serious | Serious | Not serious | Extremely serious | None | -0.01 [-0.71,0.71] | ⨁◯◯◯ Very low |
| Unclear | K = 50 | Serious | Not serious | Serious | Not serious | Publication bias | 0.23 [0.13,0.32] ***** | ⨁◯◯◯ Very low |
| Pre-exercise nutritional status | | | | | | | | |
| Fed (≤4 h) | K = 44 | Serious | Not serious | Not serious | Not serious | Publication bias | 0.22 [0.12,0.32] ***** | ⨁⨁◯◯ Low |
| Fasted (>4 h) | K = 50 | Not serious | Not serious | Not serious | Serious | Publication bias | 0.01 [-0.10,0.13] | ⨁⨁◯◯ Low |
| Unspecified | K = 20 | Not serious | Not serious | Very serious | Not serious | None | 0.10 [-0.05,0.26] | ⨁⨁◯◯ Low |
| Exercise Type | | | | | | | | |
| Aerobic endurance | K = 25 | Serious | Not serious | Not serious | Not serious | None | 0.21 [0.07,0.35] ***** | ⨁⨁⨁◯ Moderate |
| Anaerobic performance | K = 28 | Not serious | Serious | Not serious | Serious | None | 0.12 [-0.06,0.30] | ⨁⨁◯◯ Low |
| Muscular endurance | K = 29 | Not serious | Not serious | Not serious | Not serious | None | 0.04 [-0.11,0.20] | ⨁⨁⨁◯ Moderate |
| Strength/  Power | K = 32 | Not serious | Serious | Not serious | Serious | None | 0.07 [-0.05,0.20] | ⨁⨁◯◯ Low |
| Rinse Duration | | | | | | | | |
| 5 s | K = 41 | Serious | Not serious | Not serious | Not serious | Publication bias | 0.23 [0.09,0.36] ***** | ⨁⨁◯◯ Low |
| 10 s | K = 54 | Not serious | Not serious | Serious | Serious | None | 0.01 [-0.11,0.13] | ⨁⨁◯◯ Low |
| 15 s | K = 6 | Not serious | Serious | Not serious | Very serious | None | 0.25 [-0.10,0.61] | ⨁◯◯◯ Very low |
| 30 s | K = 11 | Not serious | Not serious | Not serious | Very serious | Publication bias | 0.14 [-0.15,0.44] | ⨁◯◯◯ Very low |
| Cognitive Task Type | | | | | | | | |
| Speed | K = 21 | Serious | Serious | Not serious | Not serious | Publication bias | 0.26 [-0.01,0.51] | ⨁◯◯◯ Very low |
| Accuracy | K = 32 | Serious | Not serious | Not serious | Serious | None | 0.19 [-0.09,0.47] | ⨁⨁◯◯ Low |
| ***Notes: K*:** the total number of effects included in the pooled effect size; ***Publication bias***, represented by Egger test p < 0.05.  ***:** The effect size (*Hedges’g*) was significant (p < 0.05).  **†** **GRADE Criteria for Certainty of Evidence**：  **High**: Very confident in the estimated effect.  **Moderate**: Moderately confident in the estimated effect.  **Low**: Limited confidence in the estimated effect.  **Very low**: Very limited confidence in the estimated effect. | | | | | | | | |

**Electronic Supplementary Material Appendix S15 (Sensitivity Analysis for Primary results: Exercise and Cognition)**

**Exercise:
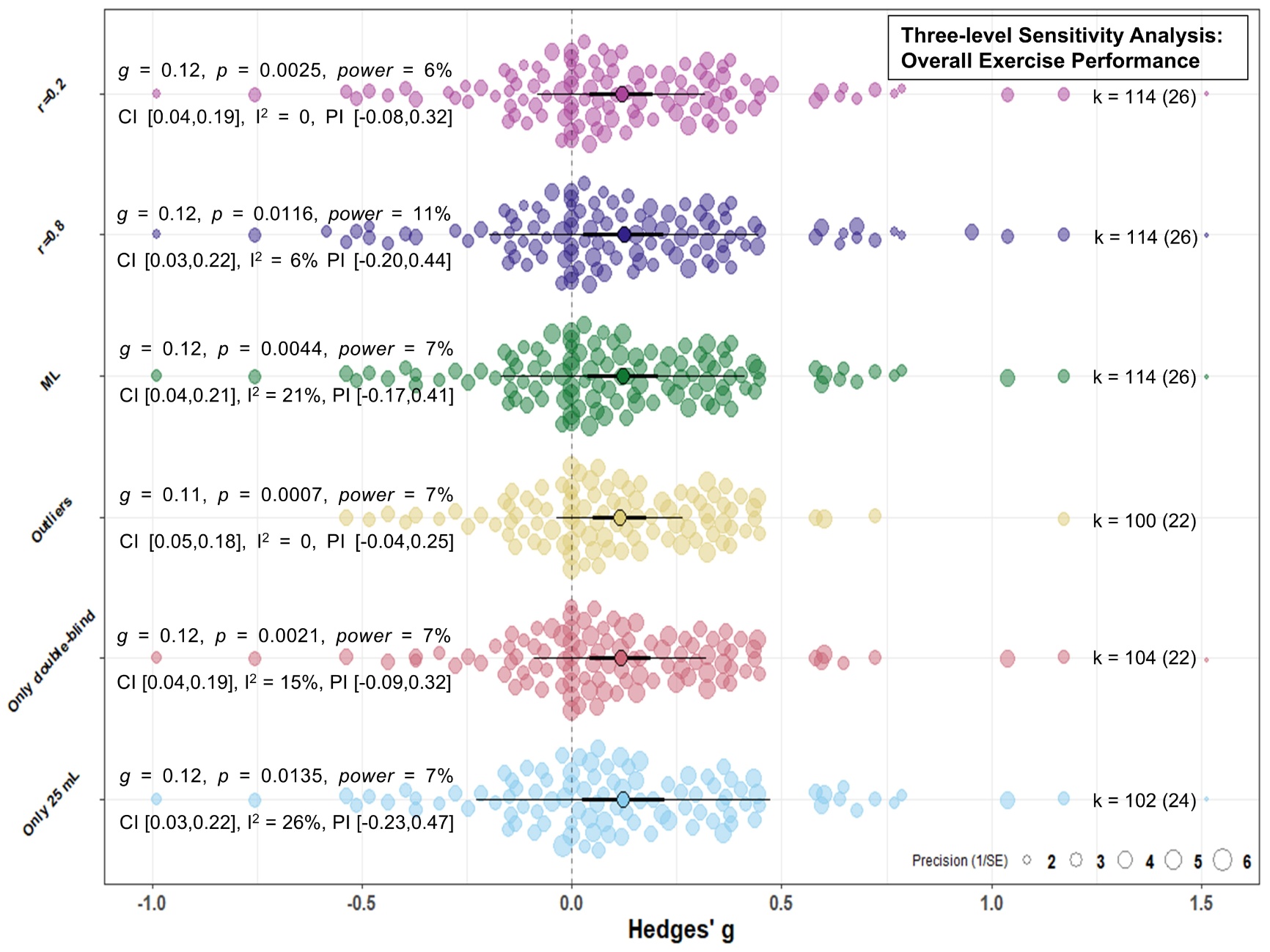
**

**Cognition:**

**
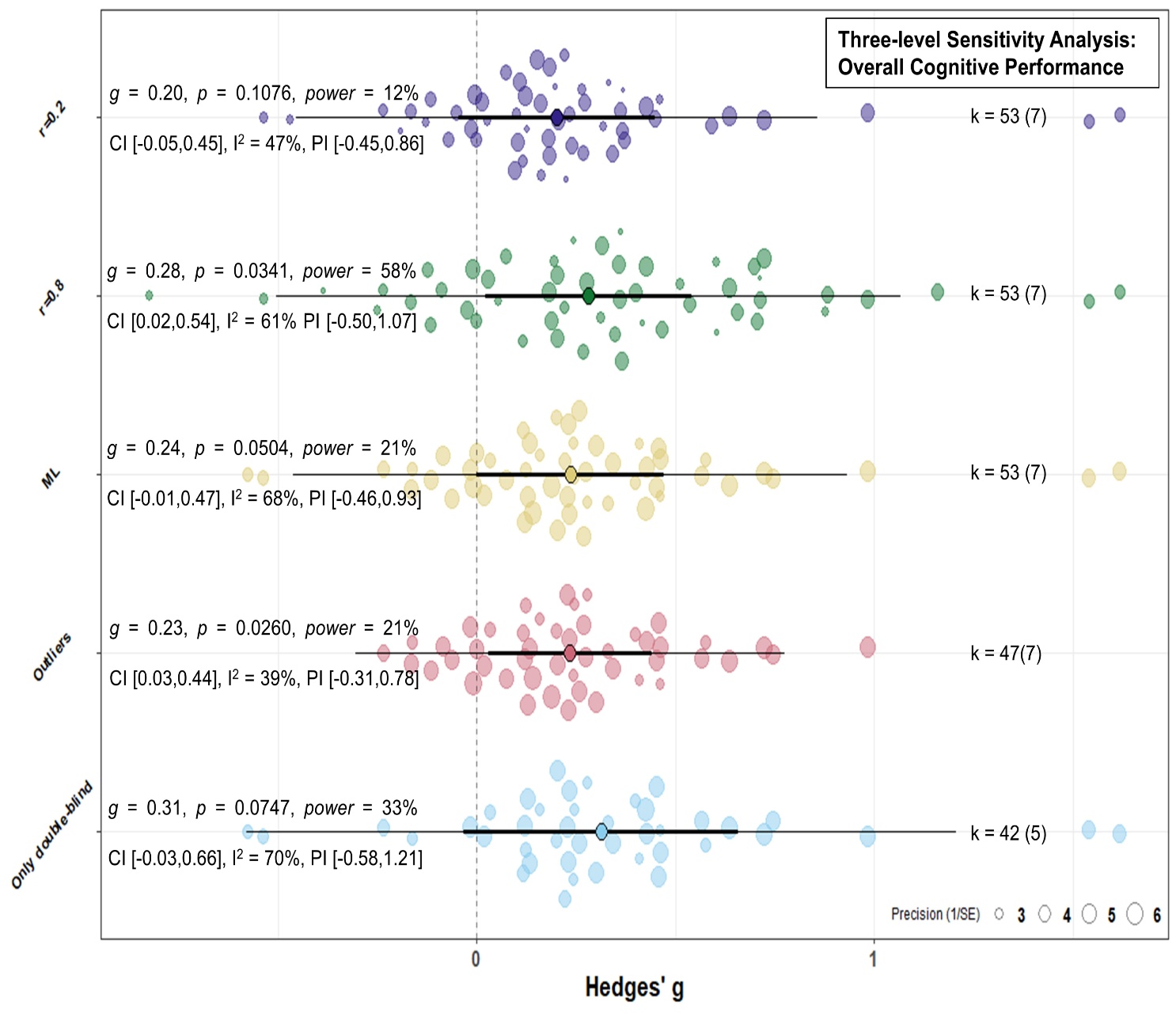
Notes: *K*,** the total number of effects included in the pooled effect size; ***Hedge's g***, the effect size indicators used in the pooled; ***CI***, 95% confidence interval; ***PI***, prediction Interval; ***P-value***, statistically significant P values ​​for pooled results; ***r***, correlation coefficient between taurine group and placebo group; ***ML***, maximum likelihood model; ***I*^2^**, quantitative indicators of heterogeneity.

**Electronic Supplementary Material Appendix S16 (A Sensitivity Analysis based on level 2 and level 3 Leave-one-out: Exercise and Cognition)**

**Exercise:**


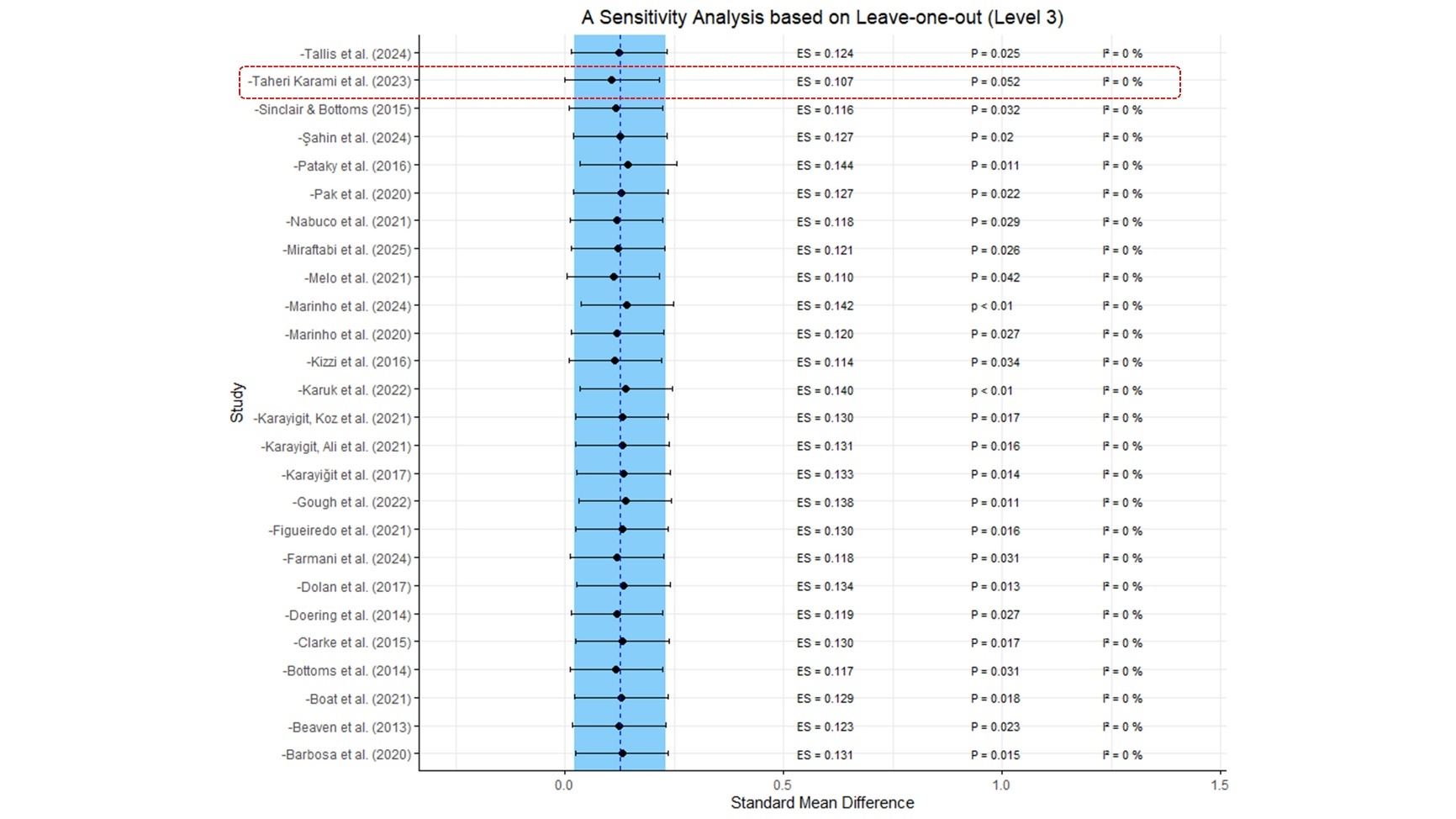


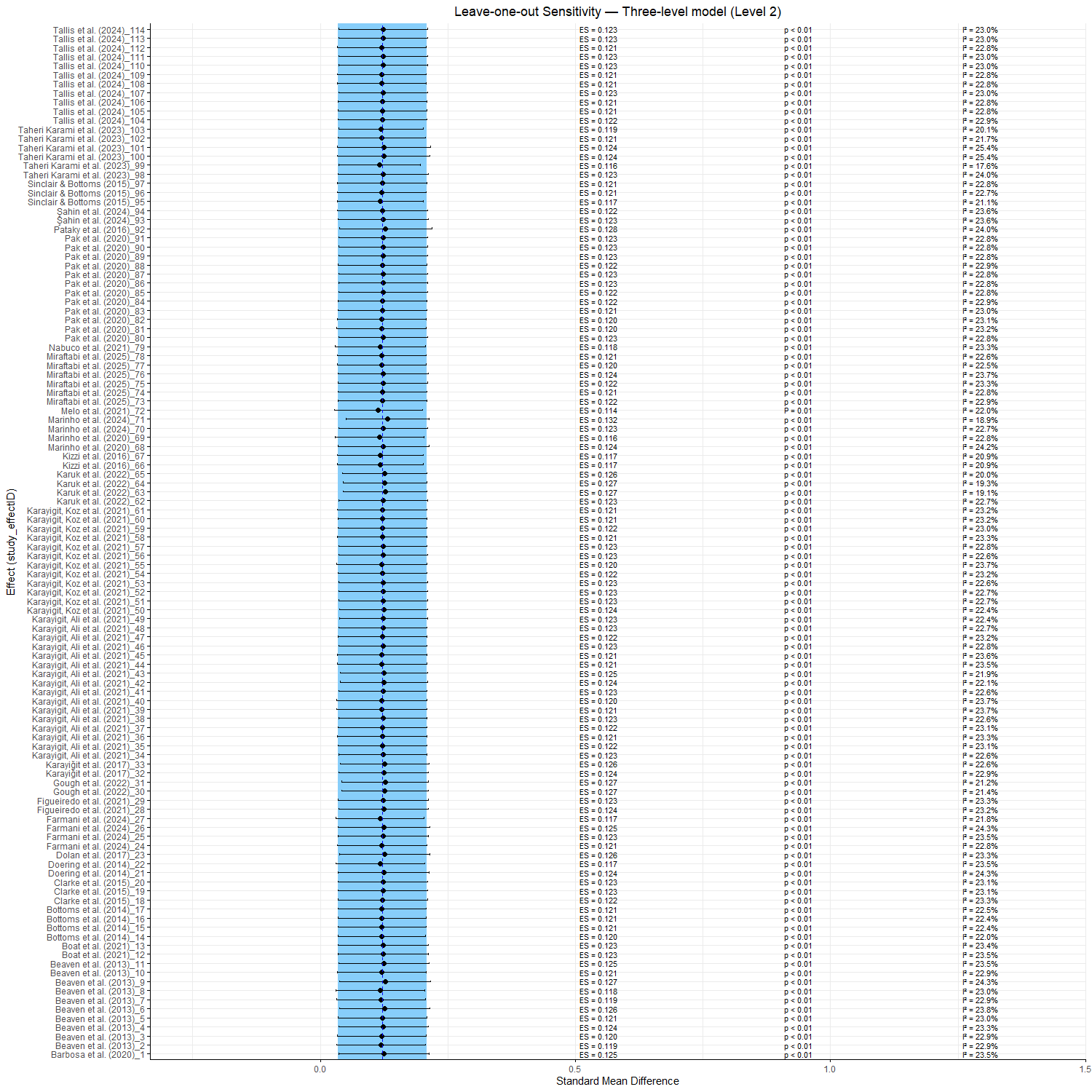


**Cognition:**


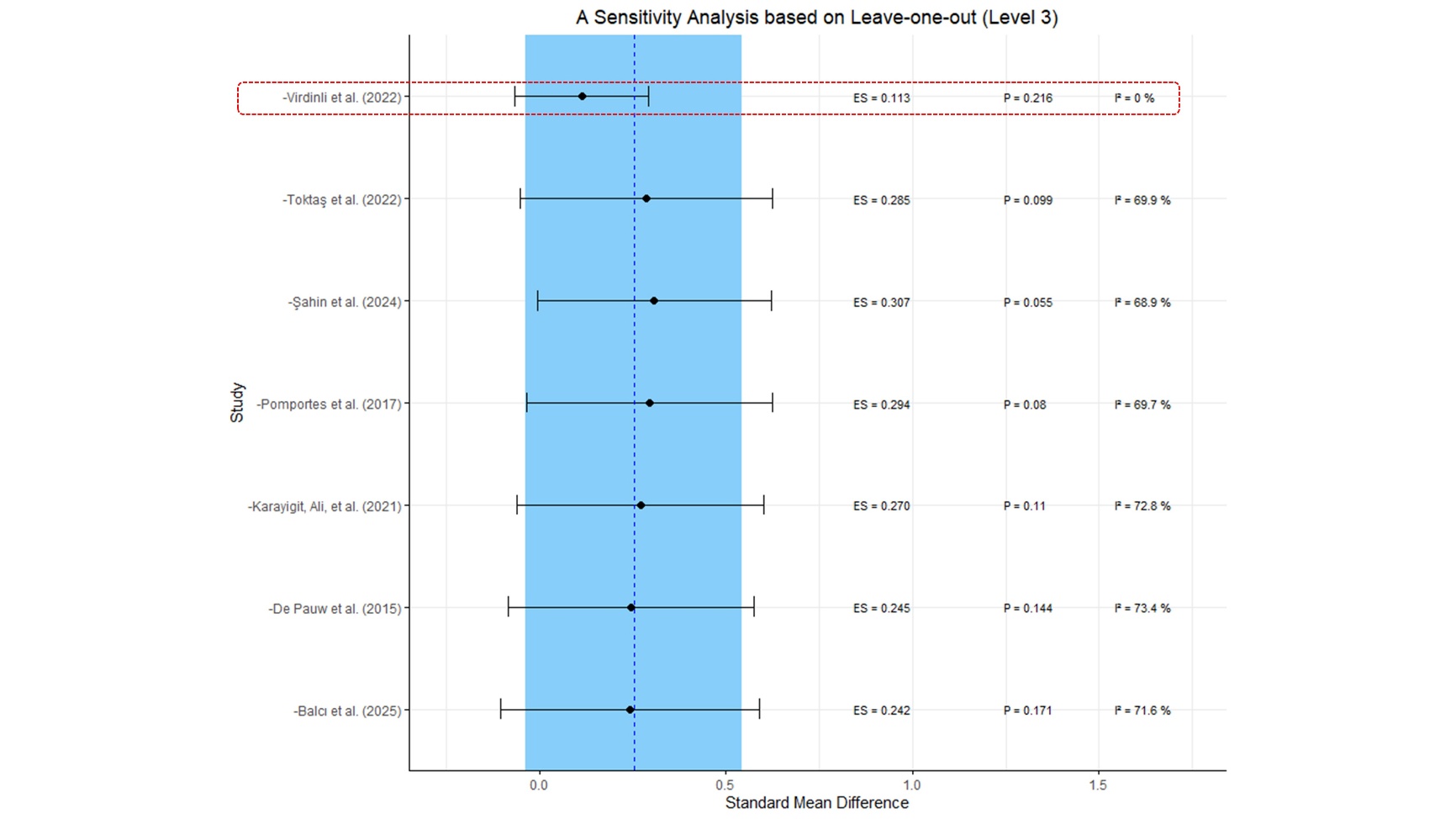


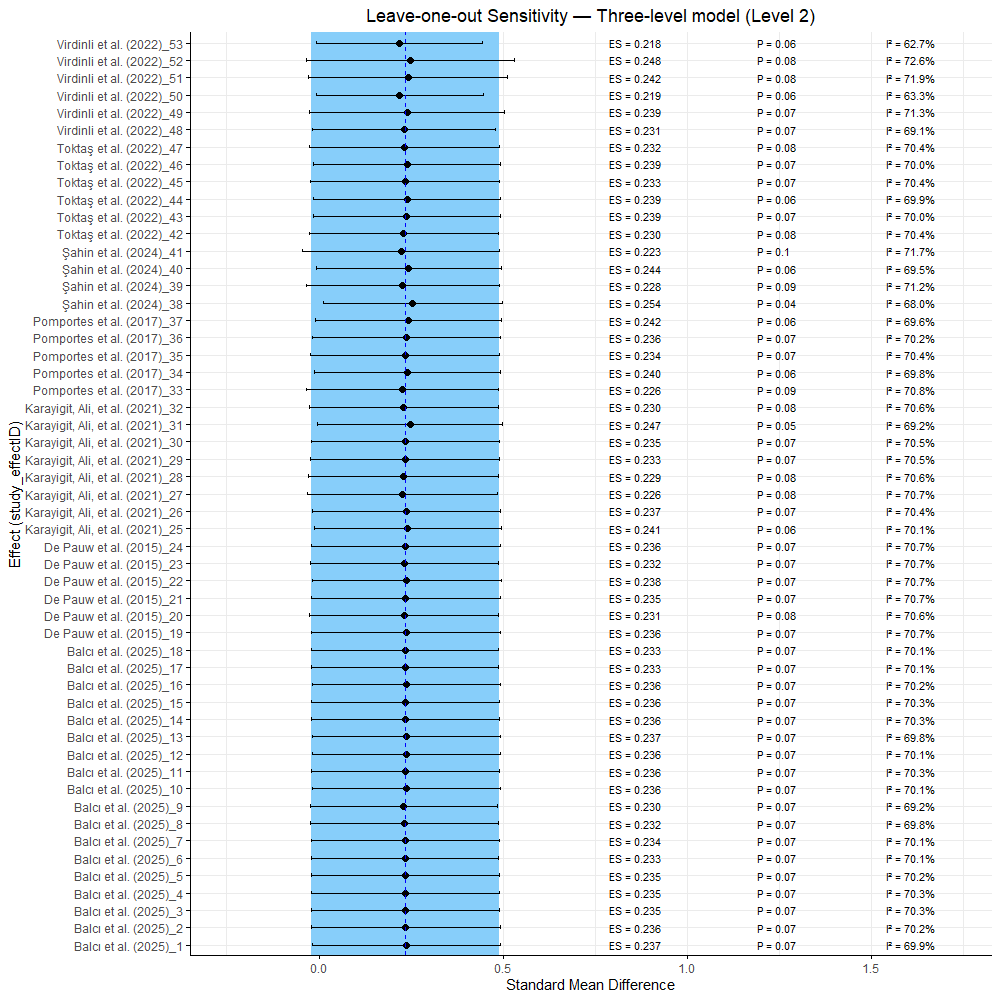


**Electronic Supplementary Material Appendix S17 (Moderator Analysis After Excluding Outliers: Exercise and Cognitive performance)**

**Exercise:**

**
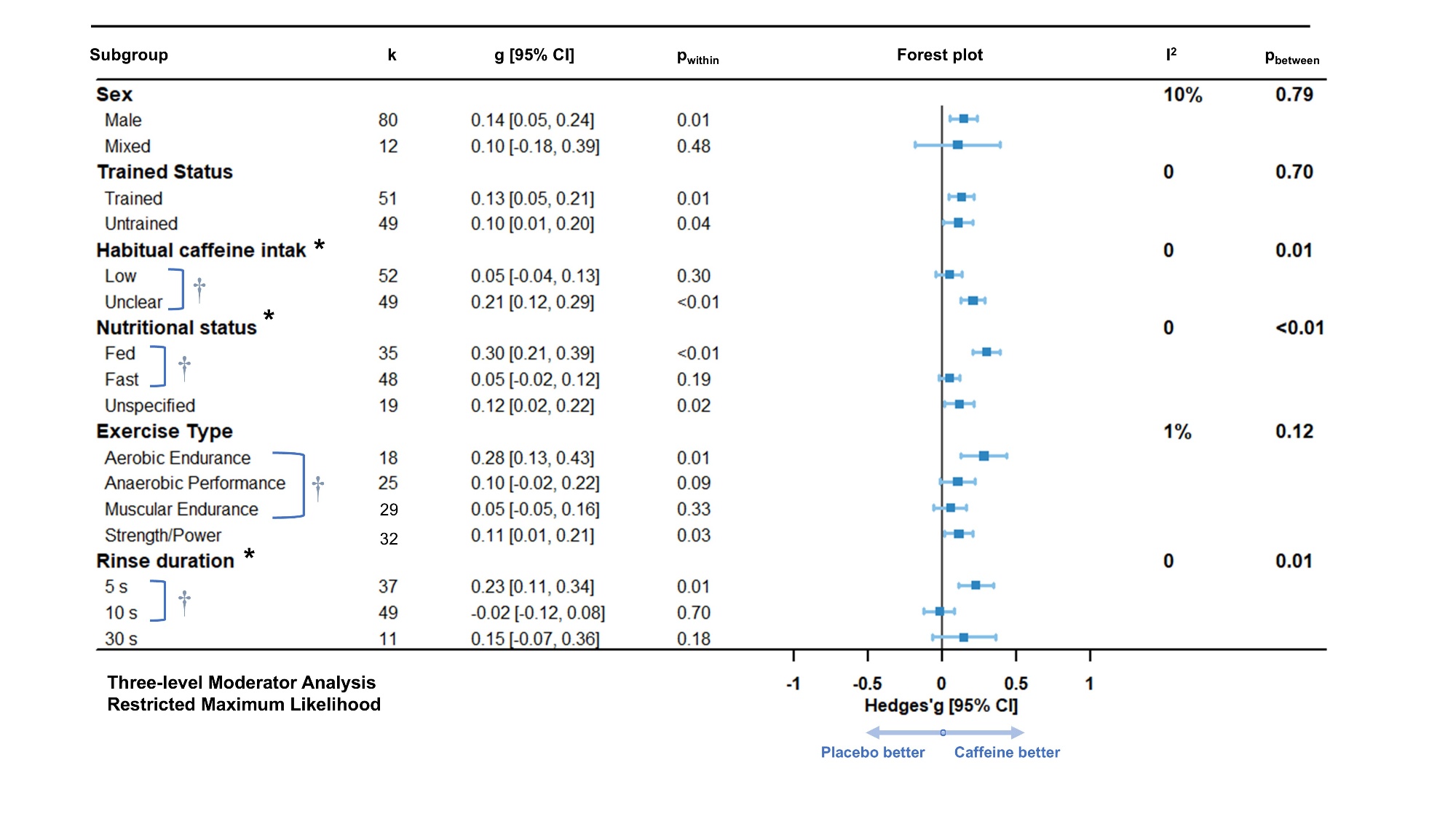
**

**Cogniton:
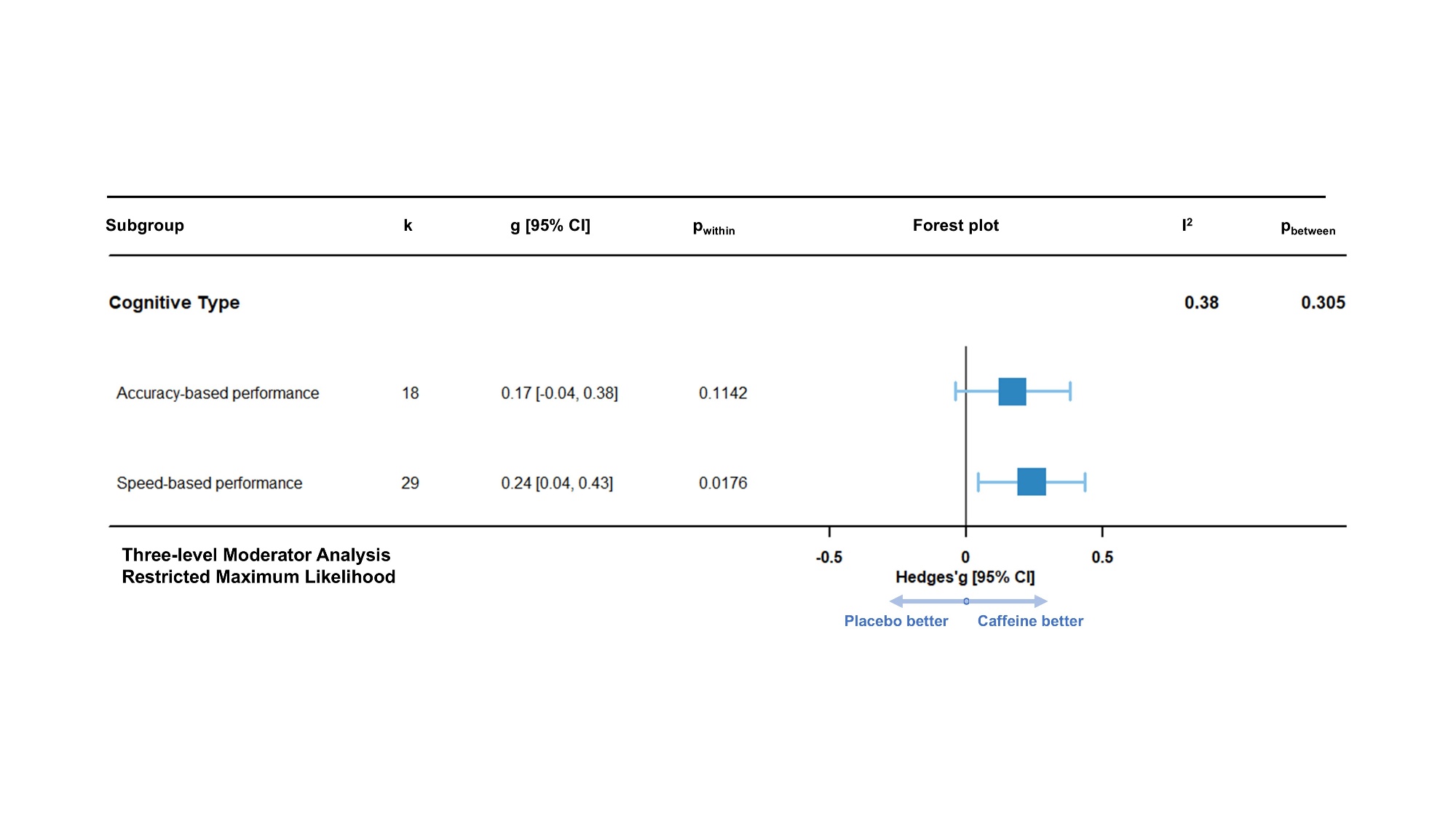
**

***Notes: K*,** the total number of effects included in the pooled effect size; ***Hedge's g***, the effect size indicators used in the pooled; ***95%CI***, 95% confidence interval; ***P_between_***, statistically significant P values ​​for pooled effect between moderator; ***P_within_***, statistically significant P values ​​for specific pooled effect of moderator; ***I*^2^**, quantitative indicators of heterogeneity; *******, represents significant differences between groups; ***†***, represents significant difference between the two categories within the group.
